# Model-Based Approach for Designing an Efficient Bioequivalence Study for Highly Variable Drugs

**DOI:** 10.3390/ph14111101

**Published:** 2021-10-28

**Authors:** Eunjung Song, Woojoo Lee, Bo-Hyung Kim

**Affiliations:** 1 Department of Clinical Pharmacology and Therapeutics, Kyung Hee University Medical Center, Seoul 02447, Korea; yue0327@khu.ac.kr; 2 Department of Public Health Sciences, Graduate School of Public Health, Seoul National University, Seoul 08826, Korea; 3 East-West Medical Research Institute, Kyung Hee University, Seoul 02447, Korea

**Keywords:** highly variable drugs, reference-scaled average bioequivalence, pharmacokinetic model

## Abstract

The statistical procedures as outlined by the European Medicines Agency (EMA) and United States Food and Drug Administration (FDA) guidelines for bioequivalence testing of highly variable drugs (HVDs) are complex. Additionally, the sample size is affected by clinical study designs or practical real-world problems, such as dropout rate or study budget. To overcome these difficulties, we propose a model-based approach for the selection of a study design with a sample size that satisfies the bioequivalence criteria using simulation studies based on a pharmacokinetic (PK) model. The designed approach was implemented using a simulation procedure considering some conventionally measured factors, such as geometric mean ratio and within-subject coefficient of variation, with various PK information important in determining bioequivalence. All simulation results were assessed according to the EMA and FDA guidelines. Furthermore, power calculations from simulation results were interpreted with regard to PK characteristics and compared among 2 × 2, 3 × 3, and 2 × 4 crossover designs to determine the efficient design considering appropriate sample size and duration of the clinical study. The proposed approach can be applied to bioequivalence studies of all drugs. However, the current study was targeted at HVDs, which are highly likely to require detailed decision making for sample size and study design.

## 1. Introduction

A bioequivalence study is performed to evaluate the therapeutic equivalence between the reference (branded) formulation and test formulation by showing that a test formulation is absorbed into the body at the same rate and extent as a reference formulation. The average bioequivalence (ABE) approach is a method for bioequivalence assessment between two formulations and is suggested by regulatory agencies such as the European Medicines Agency (EMA) and the United States Food and Drug Administration (FDA) [1,2]. The 2 × 2 clinical trial crossover design is generally used for determining the ABE through the use of two formulations, two sequences, and two periods. Pharmacokinetic (PK) parameters for the assessment of bioequivalence are determined from drug plasma concentration, which is the area under the concentration versus time curve (AUC), and the peak drug concentration (C_max_). To evaluate bioequivalence, a 90% confidence interval (CI) for the geometric least square mean ratio (GMR) between a test and a reference formulation is calculated for the C_max_ and AUC. The two formulations are considered to be bioequivalent when both confidence intervals fall within the bioequivalence acceptance range of 80–125%. This procedure is called the two one-sided test procedure (TOST) [3,4].

A within-subject variability of PK parameters is a major determinant of bioequivalence because a high within-subject variability increases the 90% CI of the GMR. If the resultant 90% CI falls outside the aforementioned bioequivalence acceptance range, bioequivalence cannot be inferred for the two drugs, even when the GMR is close to 100%. In particular, a drug is called a highly variable drug (HVD) when its within-subject variability of the C_max_ or AUC ≥ 0.294. In bioequivalence studies for HVDs, a larger sample size may be needed to satisfy the bioequivalence acceptance criteria owing to their large within -subject variabilities. However, a high variability in C_max_ or AUC is related to the dispositional characteristics of drugs, which is less connected to formulation performance assessment [5]. The EMA and FDA have raised concerns regarding conducting bioequivalence studies using large sample sizes for reasons not related to the safety and efficacy of the drug [5,6]. They suggested that the reference-scaled average bioequivalence (RSABE) approach with a GMR restriction of 80–125% be applied for the bioequivalence tests of HVDs [2,5,6,7,8]. The RSABE approach is a method in which the acceptance range of bioequivalence is expanded from 80–125% as the within-subject variability of the reference product (σ_WR_) increases [9].

The RSABE approach is applied when the S_WR_, which is the estimated σ_WR_, is >0.294, or the within--subject coefficient of variation (within-subject CV or intra-subject CV) is >30%. Otherwise, bioequivalence is tested using the unscaled average bioequivalence method, that is, considering the conventional acceptance range. In other words, even for drugs known as HVDs, the RSABE approach is not applied when S_WR_ < 0.294. Additionally, there are various components that complicate the statistical procedures for bioequivalence tests of HVDs, viz., GMR constraints and the implementation of RSABE [10]. These components are also closely related to each other in bioequivalence test procedures, which could make it difficult to calculate theoretical sample sizes. Because of these difficulties, Tothfalusi and Endrenyi [10] suggested a sample size calculation method for bioequivalence assessment of HVDs through simulation studies using the linear mixed model (LMM). This simulation study reported the minimum sample size required at the predefined conditions of statistical power, type I error, within-subject CV, and GMR.

A previous study that directly employed the LMM had limitations in implementing an efficient design with an appropriate sample size. First, the investigators had difficulties reflecting physiologic characteristics for test and reference drugs to the LMM. For example, it can be difficult for the LMM to express the correlation between individual PK parameters in a simulation study. Second, the study approach only considered the calculation of sample sizes for HVDs; therefore, it could not facilitate the selection of an efficient design. However, bioequivalence studies for HVDs are highly likely to have a problem of design selection because the S_WR_ for HVDs cannot be calculated in a 2 × 2 design and can only be obtained using 3-period or 4-period replicated designs. Even for drugs reported as HVDs, there could be cases where the S_WR_ is estimated to be <0.294 [11]. Researchers should consider the possibility that a 2 × 2 design with a sufficiently large sample size is more efficient than a 2 × 4 design or a 3 × 3 design. Additionally, the budget for clinical trials should be considered, as the cost is determined based on the trial design. Furthermore, there is a realistic problem related to study completion, that is, the dropout rate or the number of hospitalizations could increase during the study period. These factors should be considered during the planning phase of clinical trials to optimize cost-effectiveness and efficiency in drug development. To address these considerations, approaches integrating PK principles and simulations into drug development have been widely discussed as useful tools for decision-making [12,13,14,15,16,17]. Kim et al. [15] studied BE results under scenario 1 (multiple-dose, 2-period, crossover design, BE studies with ABE) and scenario 2 (single-dose,4-period, fully replicated design, BE studies with RSABE) based on a PK-model based simulation. Karalis and Macheras [16] performed a simulation study with PK parameters to compare the method proposed by EMA with existing methods in two-stage BE designs. Cai et al. [17] performed a PK model-based simulation study to evaluate the operating characteristics of a partial-block randomized crossover design for a drug with a long half-life. However, the results of these studies are not directly applicable to considering sample size and clinical trial design simultaneously in bioequivalence assessment of HVDs.

We propose a novel simulation approach based on PK models to compensate for the limitations of the LMM-based method. The proposed PK-model based simulation approach presents a computational tool for simultaneously determining an efficient study design and appropriate sample size that satisfy bioequivalence criteria. Because studies are based on PK models, the relationship between PK parameters (e.g., clearance and volume of distribution) can directly be applied to the simulations. Researchers can also investigate various within-subject variabilities of PK parameters using these simulations. Furthermore, the proposed simulation procedure can incorporate realistic factors, such as the dropout rate, into the studies. The integration of these various information can provide a more useful decision-making basis than the LMM-based method in terms of selecting clinical trial designs and sample sizes. Because the results from the simulations could be interpreted in terms of PK properties or other considerations. The reported PK model-based approach can be applied to all drugs, making it easy to provide useful information for clinical trial planning. However, drugs with within-subject CV values < 30% employ the ABE approach with a 2 × 2 crossover design and therefore have fewer considerations than HVDs. For this reason, we focused on HVDs, which are highly likely to require more sophisticated decisions with regards to selecting a clinical design because of evaluating bioequivalence using a complex procedure.

## 2. Results

### 2.1. Comparison between an Existing Study and the Current Study

**Figure 1:**
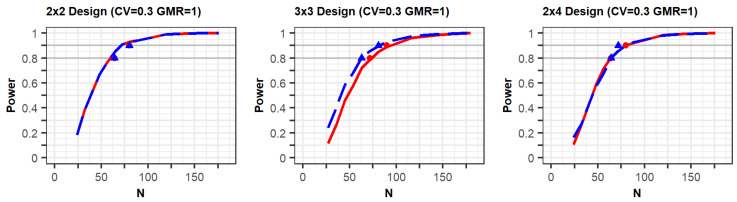
Comparison between results of the proposed method and a previous study: The power curves as per the EMA and FDA guidelines are represented as the red solid lines and blue dashed lines, respectively. The red circle points (EMA) and blue triangle points (FDA) indicate the total number of observations (N) of 80% and 90% power in the previous study [10], respectively.

The PK parameters in “variability for comparisons with a previous study” in Table 1 were selected to generate conditions similar to those of a previous study [10] in terms of individual effects and within-reference CVs. The simulation results for the conditions are shown in Figure 1 (Supplementary File S1). The figures show that the statistical power increased according to the total number of trials (N). In the three figures with a CV of 0.3, the shapes of the power curves based on the EMA and FDA guidelines were similar. The statistical powers observed from the previous simulation study, indicated as points in the figure, were slightly below or similar to the power curves of the present simulation, except in one case. One of the previous results generated as per the FDA (90% power) showed slightly higher power than the current results; however, the difference between them was not large. These similar results indicate that the approach based on the PK model was reliable in that the power reported in the previous study can similarly be obtained when using the proposed approach under the conditions outlined in Table 1.

### 2.2. Comparison of Designs

To compare the statistical power in all the study designs with various values of within-subject CVs, including the CV ≥ 0.3 for HVDs, a study design with a CV of 0.2, which is <0.3, was also simulated. When the CV was set to 0.3 for simulation, CV values > or <0.3 were generated (sampled) in simulation cases. These cases were reported as “borderline HVDs” in a previous study [11]. In other words, a borderline HVD is a compound whose CV can be estimated to be > or <0.3. Meanwhile, some drugs have been reported to have a CV > 0.3 in all bioequivalence studies, and these drugs are termed “consistent HVDs” [11]. These consistent HVDs were determined using a simulation in which the CV was set to 0.6. This simulation was confirmed by a CV > 0.3 for all datasets.

**Figure 2:**
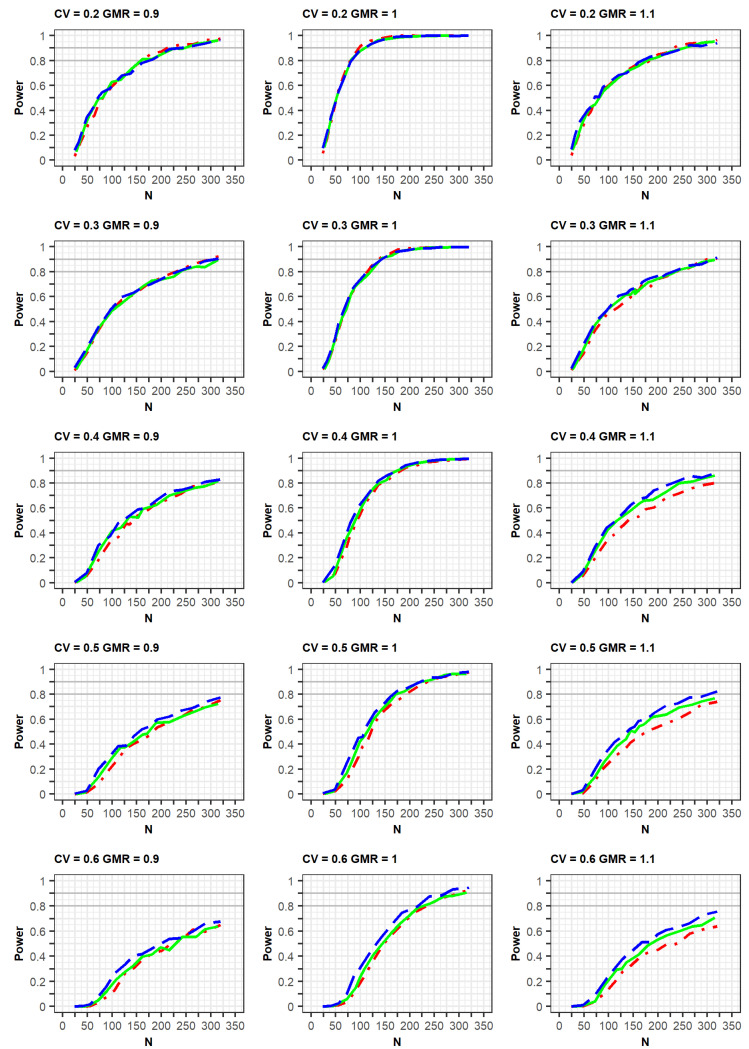
Comparison of power calculated as per the EMA guidelines among clinical trial designs: 2 × 2 design (red dot–dashed line), 3 × 3 design (green solid line), and 2 × 4 design (blue long-dashed line). The x-axis and y-axis represent the total number of trials (N) and statistical power, respectively.

**Figure 3:**
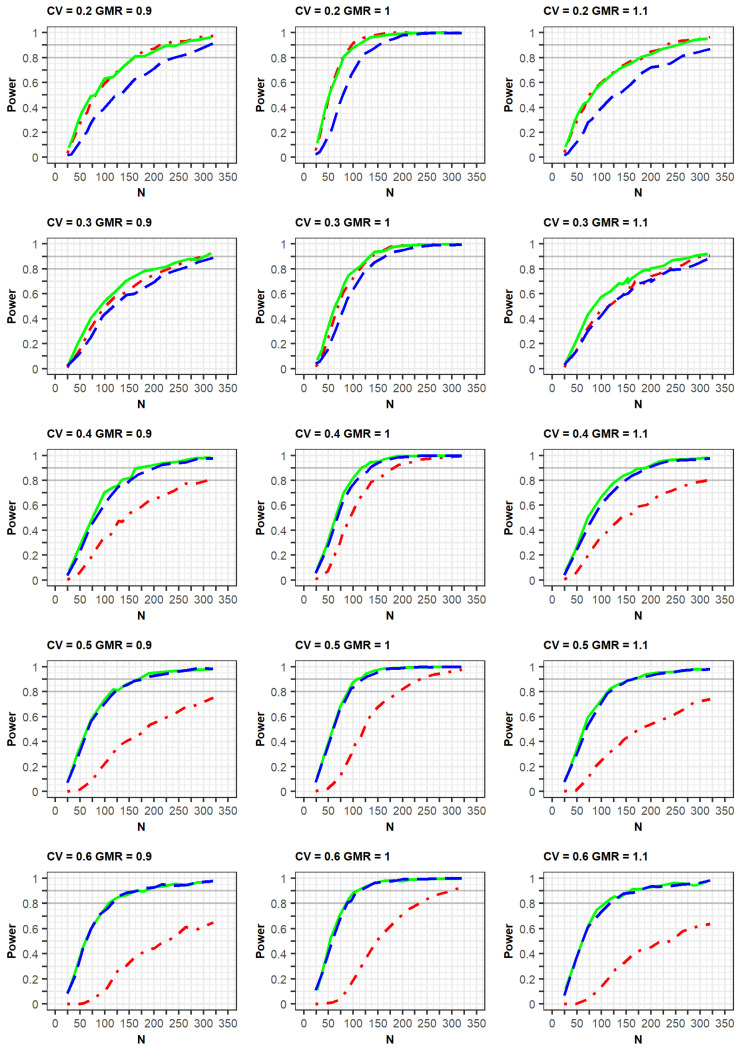
Comparison of power calculated as per the FDA guidelines among clinical trial designs: 2 × 2 design (red dot–dashed line), 3 × 3 design (green solid line), and 2 × 4 design (blue long–dashed line). The x-axis and y-axis represent the total number of trials (N) and statistical power, respectively.

If the estimated CV of the reference drug in the simulated data was <0.3, the ABE method was applied. If the estimated CV was ≥0.3, the RSABE method was applied. All simulation results indicated that the powers rapidly increased at a GMR of 1.0, compared with GMRs of 0.9 or 1.1 (Figure 2 and Figure 3, Supplementary File S2).

In the simulation results obtained as per the EMA guidelines, a similar increasing trend of power curves was observed in all the designs (2 × 2, 3 × 3, and 2 × 4) of the crossover studies. All designs were analyzed using a GLM according to the EMA guidelines. In detail, the statistical powers increased with a similar trend in the three crossover designs when the within-subject CV was 0.2 or 0.3. However, when the CV was 0.5 or 0.6, the powers were different among the three designs, with the power of the 2 × 2 design being the lowest. As the CV increased, more total number of observations (N) were needed to obtain the same power for the 3 × 3 and 2 × 4 designs. Specifically, large total number of observations (N) were required to attain 80% or 90% power at CV values of 0.5 or 0.6.

Meanwhile, in the simulation results obtained as per the FDA guidelines, a similar trend of power curves at within-subject CV of 0.2 were observed for the 2 × 2 and 3 × 3 crossover designs. However, the powers of the 2 × 4 design were relatively lower than those of the 2 × 2 and 3 × 3 crossover designs. These findings can be explained by the standard error of the mean difference in μ_T_-μ_R_. When the LMM was fitted using the SAS code proposed by the FDA, the standard error estimated in the simulation data for the 2 × 4 design was greater than the estimated standard error from the simulation data obtained using the 2 × 2 and 3 × 3 designs [7].

In the case of a CV of 0.3, the power of the 3 × 3 design was slightly higher than that of the other designs; however, the difference in the power curves between the 2 × 4 design and the other two designs diminished at a CV of 0.3, compared with that of the CV of 0.2 as mentioned above. This increase in power could be explained by the fact that the mixed models were less used when the CV was 0.3, compared with when it was 0.2. With regard to the CV ≥ 0.4, the powers of the 2 × 2 design were much lower than those of the other designs. Additionally, the powers of the 3 × 3 design were similar to those of the 2 × 4 designs, in which the mixed model was rarely used. With the exception of a CV of 0.3, the increasing rates of power for the 3 × 3 and 2 × 4 designs were similar for the same GMR. This is because of the difference in statistical analysis, ABE or RSABE, according to the CV size. Alternatively, when the CV was 0.3, the increase in power was reduced.

### 2.3. Comparison of Power by Simulation Conditions

**Figure 4:**
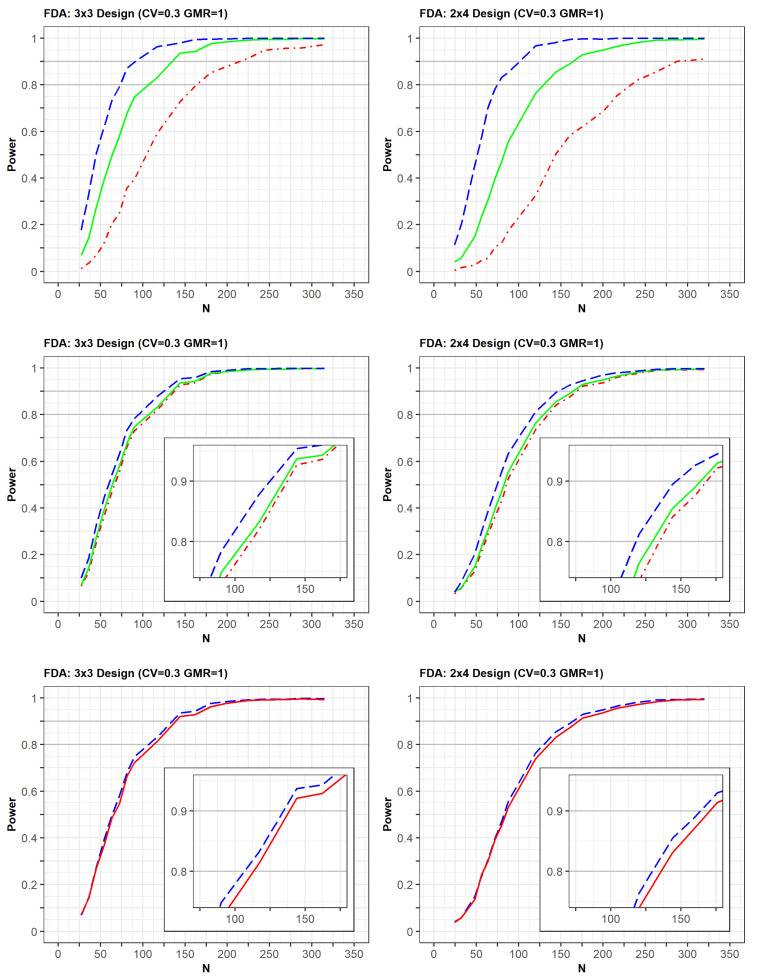
Comparison of power among clinical trial designs by (1) correlation coefficient of bioavailability between reference and test drugs [upper panel: 0.7 (red), 0.9 (green), 1.0 (blue)]; (2) correlation coefficient of absorption rate constant (Ka) between reference and test drugs [middle panel: 0.7 (red), 0.8 (green), 1.0 (blue)], (3) typical value of Ka [below panel: 1.34 (blue), 1.38 (red)]. The x-axis and y-axis represent the total number of trials (N) and statistical power, respectively.

The differences in PK parameters or variabilities affected the statistical power of the designs (Figure 4). The power was observed to increase faster when there was a strong positive correlation of bioavailability between the reference and test products in the same subject. Similarly, the power increased slightly faster as the correlation coefficient between two random effects of Ka for the reference and test drugs was closer to 1.0. Alternatively, the increase in power slightly decreased as the differences in typical values of Ka between the test and reference drugs were relatively large. At a CV of 0.3, the trends of the power curves were similar between those generated as per the EMA and FDA guidelines using identical simulation conditions, that is, changes in the correlation of bioavailability and Ka, as well as differences in Ka parameters (Supplementary File S3).

The presence of dropouts and the absence of errors in concentrations were considered to be factors that could affect power (Supplementary File S4). First, a simulation study was conducted to assess the changes in the power curve with regards to dropout. The dropout rate increased proportionally to the increment in the study duration because more subjects withdrew as the period of the clinical study increased. Therefore, the dropout rates were 3%, 4%, and 6% in the second period of the 2 × 2, 3 × 3, and 2 × 4 designs, the third period of the 3 × 3 and 2 × 4 designs, and the fourth period of the 2 × 4 design, respectively.

The increasing rate of power slightly decreased with dropout compared with non-dropout designs. Additionally, the trends of power curves among designs were very similar between the simulation results with and without dropouts. Second, when the EMA guideline was applied, the exclusion of errors (proportional and additive errors) in concentrations slightly increased the power for all designs. The power for the 2 × 2 design improved following the use of the FDA approach which allows for the exclusion of errors in concentrations. However, there were little power changes in the 3 × 3 and 2 × 4 crossover designs after using the FDA approach. These small changes could be explained by the analytical method of the FDA for 3 × 3 and 2 × 4 designs, in which the errors of C_max_ or AUC could be eliminated in the calculation of response variables ([AUC_test_–AUC_ref_], [AUC_ref1_–AUC_ref2_]).

### 2.4. Comparison of Power Using Clopidogrel Data

In the post-hoc power analysis of clopidogrel data, the within-reference CVs for C_max_ and AUC were estimated to be over 37% and 47%, respectively (Table 2). Although the CVs of both PK measurements were considerably >30%, the post-hoc powers in the 2 × 2 and 3 × 3 crossover designs were over 90%. This implies that the use of 2 × 2 design in bioequivalence assessment for clopidogrel is likely to be justified if the sample size is sufficient to satisfy the desired power within an acceptable number of subjects. However, as the sample size (or total number of observations) decreased, the post-hoc power of the 2 × 2 design decreased faster than that of the 3 × 3 design. In particular, in the 2 × 2 design, when the sample size was decreased from 64 to 42 (from 128 to 84 in terms of N), the power decreased from 93.4% to 56.5%. As the sample size changed from 43 to 28 in the 3 × 3 design, the power decreased from 99.8% to 78.2% and from 100% to 95.5% for EMA- and FDA-generated simulations, respectively. Therefore, when it is difficult to recruit a sufficient sample size, it may be advantageous to employ the RSABE approach using a 3 × 3 or 2 × 4 design to evaluate the bioequivalence of HVDs.

## 3. Discussion

The current study showed that a simulation study is important to determine the appropriate sample size and to select an efficient design for bioequivalence studies. This study also showed that the simulation results were reliable by comparing the results of two methods, viz., the PK model-based approach and the LMM-based approach [10]. Our study was designed to simulate PK parameters with various random effects and compare the bioequivalence results from 2 × 2, 3 × 3, and 2 × 4 crossover designs. This approach provides a computational tool for the selection of an efficient design beyond the simple calculation of the sample size. Using the real-world PK data of clopidogrel, we showed that a 2 × 2 study design could acquire enough power in a bioequivalence test for HVDs with an acceptable and sufficient sample size. The proposed approach is generally applicable to HVDs other than clopidogrel, as long as the researcher’s PK model fits actual PK data well.

Bioavailability should be the decisive factor in the calculation of sample size and selection of the study design for bioequivalence studies. The simulation study based on the PK model was designed to include different values of GMRs, which can be explained by the bioavailability ratio between the test and reference drugs. We also used the random effect of bioavailability related to the within-subject variability of C_max_ and AUC for the implementation of characteristics of HVDs. Additionally, PK parameters with various within-subject variabilities and GMRs, borderline HVDs (e.g., within-subject CV = 0.3), and consistent HVDs (e.g., within-subject CV ≥ 0.4) showed differing trends in the increment of statistical power according to the total number of observations (N) for 2-period, 3-period, or 4-period designs. In the case of a borderline HVD at the same N, the powers of the 2-period design were similar to the powers of the 3-period and 4-period designs. We argue that the 2-period design could be preferentially considered over the other designs in the evaluation of bioequivalence of borderline HVDs. The actual study duration of a 2-period design is relatively shorter than that of a 3- or 4-period design, and the dropout rate of a 2-period design is also lower than that of 3- or 4-period designs. However, in the case of consistent HVDs with sufficiently large within-variability (e.g., within-subject CV ≥ 0.5), statistical powers of 3-period and 4-period designs were higher than that observed for 2-period designs, and the trend was even more prominent in the results generated following FDA guidance. Therefore, 3-period and 4-period designs should be considered as priority designs when following either the FDA or EMA guidelines.

Some factors should be considered in simulation procedures for bioequivalence assessments. Ka could influence the determination of time points of maximal concentration, which should therefore be assessed using various Ka values in such studies. We speculated that the increase in statistical power would be retarded with an increase in the differences in Ka parameters between the test and reference drugs. Additionally, the error in concentrations should be considered in simulation procedures. If the error is relatively large with regard to C_max_, the variability in C_max_ can also be large. This variability in error could be important especially in relation to the bioequivalence power of HVDs. Therefore, in the current study, we evaluated the differences in bioequivalence power according to the inclusion or exclusion of errors. We also assessed the changes in bioequivalence power under conditions of typical values of Ka being largely different between a test and a reference drug, and the results showed that the correlation coefficient of bioavailability or Ka between a test and a reference drug was small. We thought that a simulation study could be achieved using already reported PK information. For example, if Ka values of both drugs are determined to be very different based on the results of the in vitro study, this difference should be included in the simulation study. Additionally, the error of concentration could be predicted by previous PK reports for the reference drug, and the impact of this error could be assessed depending on the magnitude of the predicted C_max_ in the bioequivalence study. Therefore, a simulation study should be executed following the consideration of significant PK parameters or various PK variabilities in the selection of an efficient design of bioequivalence studies for HVDs.

Under the condition of within-subject CV of 0.2, the power for the 2 × 4 design was lower than that of the 2 × 2 and 3 × 3 designs. This decrease in power can be explained by the statistical model used in the simulation study. The mixed model was used for the 2 × 4 design, and the GLM was used for the other designs according to the FDA guideline [7]. According to the FDA draft guidance and the recommendations of the EMA, the mixed model for a 2 × 4 design assumes that the random effect of each subject can be different between the test and reference drugs [7,8]. The mixed model for log-transformed C_max_ or AUC values consisted of the following five variance terms: (1) within-subject variance for a test drug, (2) within-subject variance for a reference drug, (3) between-subject variance for a test drug, (4) between-subject variance for a reference drug, and (5) between-subject covariance for the test and reference drugs. The standard error of the estimator for μ_T_-μ_R_ in the mixed model tended to be largely estimated when compared with that of the GLM, because of the last three terms of variances related to “between-subject” in the current simulation parameter conditions. The large standard error increased the 90% CI of μ_T_-μ_R_, which resulted in a decrease in power for the 2 × 4 design.

The study power can also be affected by the withdrawal of subjects, which results in missing C_max_ or AUC values. The number of withdrawal subjects was calculated as the dropout rate, which is associated with the duration of the clinical study. For example, the dropout rate proportionally increased according to the duration of the clinical study, from a 2 × 2 to a 2 × 4 design. Additionally, if the half-life of a drug is long, the duration of the clinical study could be prolonged owing to the extended washout period of the drug. Meanwhile, withdrawal of subjects results in missing C_max_ or AUC values, which should be analyzed differently according to the guidelines of the regulatory agencies (EMA or FDA). The EMA guidelines suggest that subjects should be included at least once when C_max_ or AUC is calculated for both the test and reference formulations. The FDA guidelines describe the application of the RSABE approach with two different values for C_max_ or AUC: “difference of observations (C_max_ or AUC) for each subject between two repetitive reference drugs” and “difference of observations for each subject between a test and a reference drug”. These difference values are missing values when at least one additional observation is missed. Missing values will affect the bioequivalence evaluation of HVDs. In the current study, this “missing problem” was analyzed using both the EMA and FDA guidelines, and the differences in power between missing and non-missing conditions were described for bioequivalence result changes in the case of subject withdrawals. These results might be helpful in the selection of an efficient design of a bioequivalence study involving HVDs that require a long washout period because of their long half-lives.

This simulation study was set up to calculate power according to the total number of observations (N), which was useful for comparing the power between trial designs while considering the cost. However, the increase in hospitalization days for each subject due to the change from a 2 × 2 design to a 2 × 4 design should also be considered as a time cost. If this cost function related to conducting a clinical trial is considered using the current simulation study, the result might be expected to be an excellent reference for selecting efficient designs in the resource-constrained real world.

## 4. Materials and Methods

### 4.1. Methods

Simulations were conducted to assess the bioequivalence of HVDs in various clinical trial designs. The clinical trial designs consisted of the 2 × 2, 3 × 3, and 2 × 4 crossover designs, which have various extents of within-subject variability. The various within-subject variabilities were adjusted by modifying the size of the random effect of bioavailability in the PK model. Bioequivalence assessment approaches for HVDs differ between regulatory authorities. Therefore, bioequivalence was assessed based on the EMA and FDA guidelines and both results were compared. The PK model used for the current study and the bioequivalence test methods proposed by the FDA and EMA are outlined below.

### 4.2. PK Model

Most HVDs belong to the Biopharmaceutics Classification System class II or IV and have low aqueous solubility, which can affect the rate and extent of drug absorption. Additionally, HVDs are known to have low bioavailability, resulting from extensive pre-systemic metabolism in the intestinal mucosa and extensive hepatic first-pass metabolism. These characteristics could be explained using PK parameters, namely, absolute bioavailability, apparent clearance, and apparent volume of distribution. Therefore, these PK characteristics in relation to HVDs should be implemented in a PK model to reflect these mechanistic factors, such as low bioavailability and high apparent volume of distribution. We selected clopidogrel, a widely known HVD, as a study drug for modeling and simulation [18,19]. Additionally, the clopidogrel concentration dataset for the original formulation could be used for the development of the PK model because a bioavailability study had already been conducted in the clinical trial center of Kyung Hee University Hospital. The PK parameters of clopidogrel were estimated using NONMEM^®^ (ICON Development Solutions, Ellicott, MD, USA). The final PK model was a two-compartment model showing first-and zero-order absorption kinetics; however, the model was simplified into a two-compartment model with first-order absorption kinetics for the efficient modification of PK parameters. The bioavailability parameters of the reference and test drugs were applied to the final PK model, a two-compartment model with first-order absorption kinetics, to obtain the simulated concentrations of the reference and test formulations.

### 4.3. Bioequivalence Assessment

The EMA and FDA guidelines suggest using the RSABE approach for bioequivalence testing of HVDs. The RSABE approach widens the bioequivalence acceptance range to 80–125%, based on the within-subject variability of the reference drug. To use the RSABE approach, a reference drug is administered at least twice in 3-period and 4-period replicated designs. However, the implementation of the RSABE approach as outlined in the EMA and FDA guidelines is different.

In the EMA guideline, a generalized linear model (GLM) uses log-transformed C_max_ values of the reference drug as response variables and sequence, period, and subject within sequence as explanatory variables, which are modeled as fixed effects [20]. If the S_WR_ from the GLM is ≥0.294, the RSABE approach is applied to the bioequivalence test using the C_max_; however, this approach is not used for AUC regardless of S_WR_. The treatment variable (reference or test) was included as an explanatory variable in the aforementioned GLM to calculate the GMR and its 90% CI. To justify bioequivalence, the following should be satisfied for C_max_: the estimated GMR should lie within the range of 80–125%, and the 90% CI should fall within the expanded acceptance range [lower limit (L), upper limit (U)]. The expanded acceptance range is calculated as [−exp(k × S_WR_), exp(k × S_WR_)] using the S_WR_ and the regulatory constant, k = 0.760 [2]. The limit of [L, U] can be expanded to a maximum range of [69.84–143.19%], which is the acceptable range corresponding to a within-subject CV ≥ 50%. In addition to the bioequivalence results obtained using the C_max_, the 90% CI of the GMR for AUC should be located within the range of 80–125% to declare bioequivalence between the two HVDs.

The FDA guidelines suggest the application of the RSABE approach for the bioequivalence test of HVDs. In particular, when each S_WR_ calculated from the C_max_ or AUC for a reference drug is ≥0.294, the RSABE approach can be applied [7]. However, if the S_WR_ < 0.294, the unscaled average bioequivalence approach, that is, the TOST method, should be applied for bioequivalence assessments using the C_max_ or AUC. The RSABE approach requires two response variables, viz., the difference in C_max_ or AUC between the test and reference drugs for each subject (i.e., [C_max,test_–C_max,ref_], [AUC_test_–AUC_ref_]) and the difference in C_max_ or AUC between two reference drugs for each subject (i.e., [C_max,ref1_–C_max,ref2_], [AUC_ref1_–AUC_ref2_], ref1: reference administered for the first time, ref2: reference administered for the second time) [5,7].

The two response variables should be analyzed using a GLM or LMM that has a sequence as an explanatory variable. From the results, an upper limit with a 95% CI for (μ_T_-μ_R_)^2^ − [(log(1.25)/σ_W0_)^2^] × σ_WR_^2^ should be calculated to implement RSABE, where μ_T_ and μ_R_ are the population means of the log-transformed C_max_ or AUC for the test (T) and the reference (R) products and σ_W0_ is 0.25 [5,21,22]. Bioequivalence should be declared if the following conditions are satisfied: (1) the calculated upper limit should be less than 0 with the GMR being within the range of 80–125% when the S_WR_ of the C_max_ or AUC is ≥0.294; (2) the 90% CI of the GMR for the C_max_ or AUC should be within 80–125% when the S_WR_ of the C_max_ or AUC < 0.294. This bioequivalence test procedure for HVDs is called the “mixed scaling approach”.

### 4.4. Simulation Design for Bioequivalence Assessment

**Figure 5:**
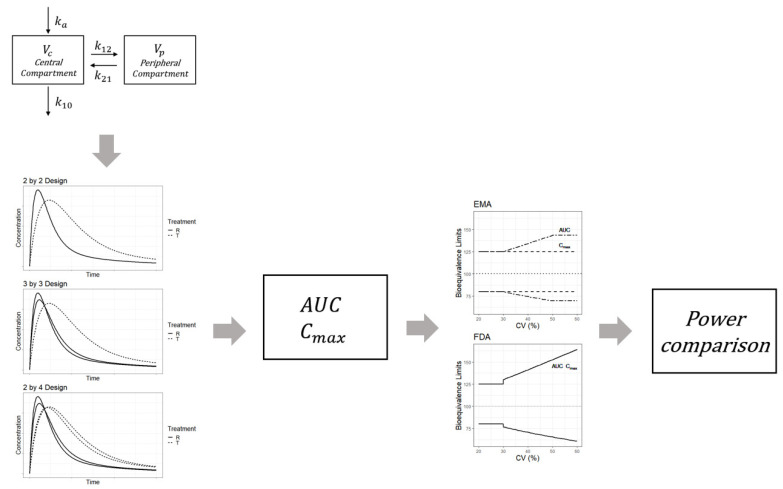
Simulation procedure for the bioequivalence assessment of highly variable drugs based on the pharmacokinetic model.

The simulations were designed to perform a bioequivalence test and compare the power between three crossover designs, viz., the 2 × 2, 3 × 3, and 2 × 4 designs. Figure 5 shows the overall simulation process. For each subject, the drug concentrations for the reference and test products were generated from the two-compartment model incorporating individual effects. PK parameters with random effects were as follows: clearance (CL), inter-compartmental clearance (Q), volume of distribution of the central compartment (Vc), volume of distribution of the peripheral compartment (Vp), absorption rate constant (Ka), and bioavailability (F). The lognormal distribution was assumed for random effects of the parameters, and the mean of the individual effects for each parameter was set to 0. Random effects were generated for all parameters. The between-subject variabilities and correlations for the random effects are summarized in Table 1. The typical values and standard deviations used in the simulation were estimated from clopidogrel data (Table 1). Because typical values of bioavailability are different between a reference and test drug, simulations were planned to reflect these differences. These differences between the two drugs are closely related to the GMR of the C_max_ and AUC of both products. The within-subject variability of bioavailability can be explained by the within-subject CV for the bioavailability parameter. The within-subject CV for bioavailability of the reference drug should be set as ≥0.3 in order to generate a concentration result for HVDs. 

Based on the PK model with typical values and assumptions, individual values for Vc, Vp, CL, Q, Ka, and F were generated for each subject. The individual PK parameters were used to generate a time-concentration curve based on Equation (1). In Equation (1), D denotes the dose, *k*_10_ denotes the elimination rate constant from the central compartment, and *k*_12_ and *k*_21_ represent the transfer rate constant from the central compartment to the peripheral compartment and the transfer rate constant from the peripheral to the central compartment, respectively. Each simulation concentration was obtained according to the corresponding design of three clinical trial designs (2 × 2, 3 × 3, and 2 × 4). After determining the C_max_ or AUC from simulated concentrations, bioequivalence between the two drugs was investigated by applying the bioequivalence test methods outlined by the EMA and FDA. Power was calculated as the rate of bioequivalence success and was compared between the trial designs.

(1) $$C\left(t\right)=\left\{\frac{K_{a}FD\;}{V_{c}}\right\}\left\{\frac{\left(k_{21}-\lambda_{1}\right)e^{-\lambda_{1}t}}{\left(\lambda_{2}-\lambda_{1}\right)\left(k_{a}-\lambda_{1}\right)}+\frac{\left(k_{21}-\lambda_{2}\right)e^{-\lambda_{2}t}}{\left(k_{a}-\lambda_{2}\right)\left(\lambda_{1}-\lambda_{2}\right)}+\frac{\left(k_{21}-k_{a}\right)e^{-k_{a}t}}{\left(\lambda_{1}-k_{a}\right)\left(\lambda_{2}-k_{a}\right)}\right\}\;\lambda_{1}=\frac{\left\{\left(k_{12}+k_{21}+k_{10}\right)-\sqrt{\left({\left(k_{21}+k_{12}+k_{10}\right)}^{2}-4k_{10}k_{21}\right)}\right\}}{2}\;\lambda_{2}=\frac{\left\{\left(k_{12}+k_{21}+k_{10}\right)+\sqrt{\left({\left(k_{21}+k_{12}+k_{10}\right)}^{2}-4k_{10}k_{21}\right)}\right\}}{2}\;k_{21}=\frac{Q}{V_{2}}k_{10}=\frac{CL}{V_{c}}k_{12}=\frac{Q}{V_{c}}$$

*k*_12_, *k*_21_: transfer rate between the central and peripheral compartments; *k*_10_: elimination rate constant.

### 4.5. Simulated Data and Visual Display

To assess bioequivalence using data, simulations were repeatedly conducted 1000 times for each condition. The statistical power was calculated to assess the 90% CI of the GMR for C_max_ or AUC within a predefined acceptance range. The power curve was displayed using the Y-axis of power and X-axis scale of “total number of observations (N)”, which was obtained from the “number of subjects (n)” × “number of period (2, 3, or 4)”. The total number of observations (N) indicates the total number of hospitalizations for all subjects. The total number of observations (N), instead of the number of subjects (n), for each trial design (2 × 2, 3 × 3, 2 × 4 crossover designs) was compared to select the most efficient design among them because the total number of observations could be a major factor in determining study cost.

### 4.6. Validation of Proposed Method

To confirm the validity of the proposed methodology, simulations were performed using a PK model with parameters that were set to obtain similar results to previously reported study results [10]. The PK parameters of the simulation were set to satisfy the following: (1) PK parameters between test and reference drugs for each subject were the same and (2) within-reference CV can be explained by within-reference subject variability of bioavailability. Briefly, within-subject variability of PK parameters, except bioavailability, was fixed at 0. The detailed PK parameters with random effects, “typical values with variability for comparisons with the previous study” are shown in Table 1.

### 4.7. Post-Hoc Power Analysis Using Real-World Data

Post-hoc power analysis was conducted to compare powers between designs with the same total number of observations (N) using real-world data from a bioequivalence study of clopidogrel [23]. The clopidogrel data were obtained from 64 subjects who were enrolled in a 3 × 3 design study. The sub-samples of the real data were extracted to construct virtual data, such as those generated in the 2 × 2 and 3 × 3 crossover designs. While making the number of observations for the designs similar, each sub-sample was constructed using cases that were randomly extracted from the available data. To organize sub-samples corresponding to a 2 × 2 cross-over design, one period was randomly sampled between two periods in which the reference was administered to each individual. For sub-sampling of the 3 × 3 design, subjects were randomly selected so that the total number of observations (N) were similar to those of the 2 × 2 design. The sub-samples were extracted for 1000 iterations to calculate the post-hoc power, defined as the rate of passing the bioequivalence test.

## 5. Conclusions

The proposed approach has many advantages; however, it may be difficult to calculate the sample size quickly and simply. The simulation procedures can be time-consuming and costly for the collection of information, the calculation of concentrations from PK models for specific drugs, and the assessment of bioequivalence. However, the previously reported simulation study contained inaccuracies because the sample size was calculated using the fixed within-subject CV and GMR without a PK model. This may limit the accurate assessment for the selection of the study design, including sample size, as it rarely reflects various complex real-world conditions. Therefore, the proposed approaches in the current study can be a good toolkit for careful study planning in situations where major decision-making is required, such as when the budget for bioequivalence research is limited.

## Figures and Tables

**Table 1:** Simulation parameters for the two-compartment pharmacokinetic model.

Parameter	Typical Value	Variability for Simulation Study			Variability for Comparisons with the Previous Study		
		BSV	WSV	Correlation	BSV	WSV	Correlation
CL (×10^3^ L/h)	29.4	0.91	-	0.5	0.91	-	0.5
Vc (×10^3^ L)	14.6	1.17	-		1.17	-	
Q (×10^3^ L/h)	1.04	1.33	-	0.5	1.33	-	0.5
Vp (×10^3^ L)	57.3	1.20	-		1.20	-	
Ka (1/h)							
Test formulation	1.34	0.42	0.1	0.8	0.42	0	1.0
Reference formulation	1.31	0.42	0.1		0.42	0	
F							
Test formulation	0.90–1.1	0.7	0.20–0.55	0.9	0.7	0.294	1.0
Reference formulation	1.0	0.7	0.20–0.55		0.7	0.294	
Concentration							
Additive error (pg/mL)	-	-	20	-	-	0	-
Proportional error	-	-	0.15	-	-	0	-

Clearance (CL), inter–compartmental clearance (Q), volume of distribution of the central compartment (Vc), volume of distribution of the peripheral compartment (Vp), absorption rate constant (Ka), bioavailability (F), between-subject variability (BSV), and within-subject variability (WSV), both of which are expressed as standard deviations. “-” indicates a value that is not set in simulation.

**Table 2:** Results of post-hoc power analysis of clopidogrel data.

Parameter	Cross-Over Design					
	2 × 2 Design		3 × 3 Design			
			EMA		FDA	
Number of subjects (n)	64	42	43	28	43	28
Total number of observations (N)	128	84	129	84	129	84
Power (%)	93.4	56.5	99.8	78.2	100	95.5
Cmax						
Within-CV (%) ^1^	44.74766	44.64610	47.98	47.746	48.42	48.21
GMR (%)	103.96	103.97	103.92	104.10	103.95	104.14
AUC						
Within-CV (%) ^1^	36.16212	36.09053	39.13	38.86	38.31	38.06
GMR (%)	100.54	100.55	100.69	100.65	100.77	100.74

^1^ Within-CV indicates within-total CV in case of a 2 × 2 design and within-reference CV in case of a 3 × 3 design.

## Data Availability

Data available in a publicly accessible repository that does not issue DOIs. Publicly available datasets were analyzed in this study. This data can be found here: https://www.sciencedirect.com/science/article/pii/S1347436718304646?via%3Dihub.

## Supplementary Materials

The following are available online at https://www.mdpi.com/article/10.3390/ph14111101/s1, Supplementary File S1: The results for Figure 1, Supplementary File S2: The results for Figure 2 and Figure 3, Supplementary File S3: The results for Figure 4 and Supplementary File S4: Dropout and Error.
