# Supplementary material for: Model-Based Approach for Designing an Efficient Bioequivalence Study for Highly Variable Drugs
Source: Pharmaceuticals (Basel). 2021 Oct 28;14(11):1101. doi: 10.3390/ph14111101 (PMC8624447; doi:10.3390/ph14111101)
Supplement: Supplementary file 1 [file pharmaceuticals-14-01101-s001.zip › [Supplementary File S1] The results for Figure 2.pdf]

Table S1. Comparison between results of the proposed method and a previous study: The simulation results are summarized in Figure 2.

Agency	Design	CV	GMR	Total number of subject	Total number of hospitalization (N)	Power
EMA	2x2	0.3	1	12	24	0.185
EMA	2x2	0.3	1	16	32	0.382
EMA	2x2	0.3	1	24	48	0.667
EMA	2x2	0.3	1	28	56	0.763
EMA	2x2	0.3	1	32	64	0.848
EMA	2x2	0.3	1	36	72	0.908
EMA	2x2	0.3	1	40	80	0.929
EMA	2x2	0.3	1	44	88	0.94
EMA	2x2	0.3	1	60	120	0.992
EMA	2x2	0.3	1	72	144	0.996
EMA	2x2	0.3	1	80	160	1
EMA	2x2	0.3	1	88	176	0.999
EMA	3x3	0.3	1	9	27	0.117
EMA	3x3	0.3	1	12	36	0.267
EMA	3x3	0.3	1	15	45	0.458
EMA	3x3	0.3	1	18	54	0.58
EMA	3x3	0.3	1	21	63	0.72
EMA	3x3	0.3	1	24	72	0.786
EMA	3x3	0.3	1	27	81	0.85
EMA	3x3	0.3	1	30	90	0.89
EMA	3x3	0.3	1	39	117	0.961
EMA	3x3	0.3	1	48	144	0.985
EMA	3x3	0.3	1	54	162	0.994
EMA	3x3	0.3	1	60	180	1
EMA	2x4	0.3	1	6	24	0.108
EMA	2x4	0.3	1	8	32	0.258
EMA	2x4	0.3	1	12	48	0.585
EMA	2x4	0.3	1	14	56	0.717
EMA	2x4	0.3	1	16	64	0.806
EMA	2x4	0.3	1	18	72	0.856
EMA	2x4	0.3	1	20	80	0.9
EMA	2x4	0.3	1	22	88	0.919
EMA	2x4	0.3	1	30	120	0.983
EMA	2x4	0.3	1	36	144	0.995
EMA	2x4	0.3	1	40	160	0.998
EMA	2x4	0.3	1	44	176	0.999
FDA	2x2	0.3	1	12	24	0.185

FDA	2x2	0.3	1	16	32	0.382
FDA	2x2	0.3	1	24	48	0.667
FDA	2x2	0.3	1	28	56	0.763
FDA	2x2	0.3	1	32	64	0.848
FDA	2x2	0.3	1	36	72	0.908
FDA	2x2	0.3	1	40	80	0.929
FDA	2x2	0.3	1	44	88	0.94
FDA	2x2	0.3	1	60	120	0.992
FDA	2x2	0.3	1	72	144	0.996
FDA	2x2	0.3	1	80	160	1
FDA	2x2	0.3	1	88	176	0.999
FDA	3x3	0.3	1	9	27	0.241
FDA	3x3	0.3	1	12	36	0.407
FDA	3x3	0.3	1	15	45	0.585
FDA	3x3	0.3	1	18	54	0.684
FDA	3x3	0.3	1	21	63	0.805
FDA	3x3	0.3	1	24	72	0.857
FDA	3x3	0.3	1	27	81	0.896
FDA	3x3	0.3	1	30	90	0.93
FDA	3x3	0.3	1	39	117	0.975
FDA	3x3	0.3	1	48	144	0.991
FDA	3x3	0.3	1	54	162	0.998
FDA	3x3	0.3	1	60	180	1
FDA	2x4	0.3	1	6	24	0.164
FDA	2x4	0.3	1	8	32	0.266
FDA	2x4	0.3	1	12	48	0.571
FDA	2x4	0.3	1	14	56	0.677
FDA	2x4	0.3	1	16	64	0.798
FDA	2x4	0.3	1	18	72	0.856
FDA	2x4	0.3	1	20	80	0.902
FDA	2x4	0.3	1	22	88	0.926
FDA	2x4	0.3	1	30	120	0.981
FDA	2x4	0.3	1	36	144	0.995
FDA	2x4	0.3	1	40	160	0.998
FDA	2x4	0.3	1	44	176	0.999
