# Supplementary material for: Model-Based Approach for Designing an Efficient Bioequivalence Study for Highly Variable Drugs
Source: Pharmaceuticals (Basel). 2021 Oct 28;14(11):1101. doi: 10.3390/ph14111101 (PMC8624447; doi:10.3390/ph14111101)
Supplement: Supplementary file 1 [file pharmaceuticals-14-01101-s001.zip › [Supplementary File S2] The results for Figure 3 and Figure 4.pdf]

Table S2. Comparison of power calculated as per the EMA and FDA guidelines among clinical trial designs: 2×2 design, 3×3 design, and 2×4 design. The simulation results are summarized in Figure 3 and Figure 4.

Agency	Design	CV	GMR	Total number of subject	Total number of hospitalization (N)	Power
EMA	2x2	0.2	0.9	12	24	0.035
EMA	2x2	0.2	0.9	16	32	0.109
EMA	2x2	0.2	0.9	24	48	0.254
EMA	2x2	0.2	0.9	28	56	0.335
EMA	2x2	0.2	0.9	32	64	0.358
EMA	2x2	0.2	0.9	36	72	0.442
EMA	2x2	0.2	0.9	38	76	0.473
EMA	2x2	0.2	0.9	50	100	0.597
EMA	2x2	0.2	0.9	60	120	0.669
EMA	2x2	0.2	0.9	70	140	0.734
EMA	2x2	0.2	0.9	80	160	0.801
EMA	2x2	0.2	0.9	90	180	0.856
EMA	2x2	0.2	0.9	100	200	0.872
EMA	2x2	0.2	0.9	108	216	0.91
EMA	2x2	0.2	0.9	120	240	0.928
EMA	2x2	0.2	0.9	132	264	0.931
EMA	2x2	0.2	0.9	144	288	0.962
EMA	2x2	0.2	0.9	160	320	0.975
EMA	2x2	0.2	0.95	12	24	0.051
EMA	2x2	0.2	0.95	16	32	0.14
EMA	2x2	0.2	0.95	20	40	0.28
EMA	2x2	0.2	0.95	26	52	0.465
EMA	2x2	0.2	0.95	32	64	0.638
EMA	2x2	0.2	0.95	36	72	0.686
EMA	2x2	0.2	0.95	40	80	0.755
EMA	2x2	0.2	0.95	50	100	0.836
EMA	2x2	0.2	0.95	60	120	0.907
EMA	2x2	0.2	0.95	70	140	0.949
EMA	2x2	0.2	0.95	80	160	0.972
EMA	2x2	0.2	0.95	90	180	0.987
EMA	2x2	0.2	0.95	100	200	0.991
EMA	2x2	0.2	0.95	108	216	0.995
EMA	2x2	0.2	0.95	120	240	0.994
EMA	2x2	0.2	0.95	132	264	0.999
EMA	2x2	0.2	0.95	144	288	0.998
EMA	2x2	0.2	0.95	160	320	1
EMA	2x2	0.2	1	12	24	0.056

EMA	2x2	0.2	1	16	32	0.165
EMA	2x2	0.2	1	20	40	0.303
EMA	2x2	0.2	1	28	56	0.578
EMA	2x2	0.2	1	36	72	0.743
EMA	2x2	0.2	1	40	80	0.801
EMA	2x2	0.2	1	50	100	0.917
EMA	2x2	0.2	1	60	120	0.96
EMA	2x2	0.2	1	70	140	0.977
EMA	2x2	0.2	1	80	160	0.99
EMA	2x2	0.2	1	90	180	1
EMA	2x2	0.2	1	100	200	1
EMA	2x2	0.2	1	108	216	0.999
EMA	2x2	0.2	1	120	240	1
EMA	2x2	0.2	1	132	264	1
EMA	2x2	0.2	1	144	288	1
EMA	2x2	0.2	1	160	320	1
EMA	2x2	0.2	1.05	12	24	0.058
EMA	2x2	0.2	1.05	18	36	0.201
EMA	2x2	0.2	1.05	24	48	0.387
EMA	2x2	0.2	1.05	28	56	0.487
EMA	2x2	0.2	1.05	32	64	0.585
EMA	2x2	0.2	1.05	36	72	0.632
EMA	2x2	0.2	1.05	40	80	0.708
EMA	2x2	0.2	1.05	50	100	0.812
EMA	2x2	0.2	1.05	60	120	0.883
EMA	2x2	0.2	1.05	70	140	0.918
EMA	2x2	0.2	1.05	80	160	0.937
EMA	2x2	0.2	1.05	90	180	0.96
EMA	2x2	0.2	1.05	100	200	0.979
EMA	2x2	0.2	1.05	108	216	0.981
EMA	2x2	0.2	1.05	120	240	0.994
EMA	2x2	0.2	1.05	132	264	0.992
EMA	2x2	0.2	1.05	144	288	0.999
EMA	2x2	0.2	1.05	160	320	1
EMA	2x2	0.2	1.1	12	24	0.039
EMA	2x2	0.2	1.1	16	32	0.119
EMA	2x2	0.2	1.1	20	40	0.202
EMA	2x2	0.2	1.1	28	56	0.346
EMA	2x2	0.2	1.1	32	64	0.393
EMA	2x2	0.2	1.1	36	72	0.489
EMA	2x2	0.2	1.1	40	80	0.523

EMA	2x2	0.2	1.1	44	88	0.559
EMA	2x2	0.2	1.1	60	120	0.671
EMA	2x2	0.2	1.1	70	140	0.729
EMA	2x2	0.2	1.1	80	160	0.775
EMA	2x2	0.2	1.1	90	180	0.789
EMA	2x2	0.2	1.1	100	200	0.851
EMA	2x2	0.2	1.1	108	216	0.863
EMA	2x2	0.2	1.1	120	240	0.922
EMA	2x2	0.2	1.1	132	264	0.937
EMA	2x2	0.2	1.1	144	288	0.94
EMA	2x2	0.2	1.1	160	320	0.964
EMA	2x2	0.3	0.9	12	24	0.01
EMA	2x2	0.3	0.9	24	48	0.145
EMA	2x2	0.3	0.9	36	72	0.329
EMA	2x2	0.3	0.9	48	96	0.479
EMA	2x2	0.3	0.9	60	120	0.573
EMA	2x2	0.3	0.9	72	144	0.613
EMA	2x2	0.3	0.9	80	160	0.664
EMA	2x2	0.3	0.9	88	176	0.71
EMA	2x2	0.3	0.9	100	200	0.753
EMA	2x2	0.3	0.9	108	216	0.78
EMA	2x2	0.3	0.9	120	240	0.819
EMA	2x2	0.3	0.9	132	264	0.861
EMA	2x2	0.3	0.9	144	288	0.889
EMA	2x2	0.3	0.9	160	320	0.932
EMA	2x2	0.3	0.95	12	24	0.023
EMA	2x2	0.3	0.95	16	32	0.047
EMA	2x2	0.3	0.95	24	48	0.204
EMA	2x2	0.3	0.95	28	56	0.28
EMA	2x2	0.3	0.95	32	64	0.388
EMA	2x2	0.3	0.95	36	72	0.489
EMA	2x2	0.3	0.95	40	80	0.568
EMA	2x2	0.3	0.95	52	104	0.703
EMA	2x2	0.3	0.95	60	120	0.811
EMA	2x2	0.3	0.95	72	144	0.852
EMA	2x2	0.3	0.95	80	160	0.913
EMA	2x2	0.3	0.95	88	176	0.929
EMA	2x2	0.3	0.95	100	200	0.955
EMA	2x2	0.3	0.95	108	216	0.968
EMA	2x2	0.3	0.95	120	240	0.978
EMA	2x2	0.3	0.95	132	264	0.984

EMA	2x2	0.3	0.95	144	288	0.995
EMA	2x2	0.3	0.95	160	320	0.993
EMA	2x2	0.3	1	12	24	0.018
EMA	2x2	0.3	1	16	32	0.044
EMA	2x2	0.3	1	24	48	0.228
EMA	2x2	0.3	1	28	56	0.338
EMA	2x2	0.3	1	32	64	0.445
EMA	2x2	0.3	1	36	72	0.53
EMA	2x2	0.3	1	40	80	0.61
EMA	2x2	0.3	1	44	88	0.662
EMA	2x2	0.3	1	60	120	0.834
EMA	2x2	0.3	1	72	144	0.921
EMA	2x2	0.3	1	80	160	0.948
EMA	2x2	0.3	1	88	176	0.978
EMA	2x2	0.3	1	100	200	0.987
EMA	2x2	0.3	1	108	216	0.992
EMA	2x2	0.3	1	120	240	0.995
EMA	2x2	0.3	1	132	264	0.999
EMA	2x2	0.3	1	144	288	0.999
EMA	2x2	0.3	1	160	320	0.999
EMA	2x2	0.3	1.05	12	24	0.018
EMA	2x2	0.3	1.05	16	32	0.036
EMA	2x2	0.3	1.05	24	48	0.188
EMA	2x2	0.3	1.05	28	56	0.299
EMA	2x2	0.3	1.05	32	64	0.367
EMA	2x2	0.3	1.05	38	76	0.496
EMA	2x2	0.3	1.05	40	80	0.517
EMA	2x2	0.3	1.05	48	96	0.636
EMA	2x2	0.3	1.05	52	104	0.684
EMA	2x2	0.3	1.05	60	120	0.719
EMA	2x2	0.3	1.05	72	144	0.845
EMA	2x2	0.3	1.05	80	160	0.86
EMA	2x2	0.3	1.05	88	176	0.885
EMA	2x2	0.3	1.05	100	200	0.936
EMA	2x2	0.3	1.05	108	216	0.952
EMA	2x2	0.3	1.05	120	240	0.974
EMA	2x2	0.3	1.05	132	264	0.978
EMA	2x2	0.3	1.05	144	288	0.978
EMA	2x2	0.3	1.05	160	320	0.988
EMA	2x2	0.3	1.1	12	24	0.012
EMA	2x2	0.3	1.1	24	48	0.138

EMA	2x2	0.3	1.1	36	72	0.329
EMA	2x2	0.3	1.1	48	96	0.458
EMA	2x2	0.3	1.1	60	120	0.519
EMA	2x2	0.3	1.1	68	136	0.569
EMA	2x2	0.3	1.1	76	152	0.658
EMA	2x2	0.3	1.1	84	168	0.699
EMA	2x2	0.3	1.1	92	184	0.679
EMA	2x2	0.3	1.1	100	200	0.74
EMA	2x2	0.3	1.1	72	144	0.594
EMA	2x2	0.3	1.1	80	160	0.645
EMA	2x2	0.3	1.1	88	176	0.701
EMA	2x2	0.3	1.1	100	200	0.743
EMA	2x2	0.3	1.1	108	216	0.761
EMA	2x2	0.3	1.1	120	240	0.805
EMA	2x2	0.3	1.1	132	264	0.83
EMA	2x2	0.3	1.1	144	288	0.893
EMA	2x2	0.3	1.1	160	320	0.904
EMA	2x2	0.4	0.9	12	24	0.004
EMA	2x2	0.4	0.9	24	48	0.058
EMA	2x2	0.4	0.9	36	72	0.183
EMA	2x2	0.4	0.9	48	96	0.334
EMA	2x2	0.4	0.9	56	112	0.369
EMA	2x2	0.4	0.9	64	128	0.473
EMA	2x2	0.4	0.9	68	136	0.469
EMA	2x2	0.4	0.9	76	152	0.54
EMA	2x2	0.4	0.9	84	168	0.572
EMA	2x2	0.4	0.9	96	192	0.643
EMA	2x2	0.4	0.9	108	216	0.671
EMA	2x2	0.4	0.9	120	240	0.716
EMA	2x2	0.4	0.9	132	264	0.776
EMA	2x2	0.4	0.9	144	288	0.777
EMA	2x2	0.4	0.9	160	320	0.813
EMA	2x2	0.4	0.95	12	24	0.009
EMA	2x2	0.4	0.95	24	48	0.077
EMA	2x2	0.4	0.95	36	72	0.266
EMA	2x2	0.4	0.95	40	80	0.338
EMA	2x2	0.4	0.95	44	88	0.42
EMA	2x2	0.4	0.95	52	104	0.535
EMA	2x2	0.4	0.95	56	112	0.584
EMA	2x2	0.4	0.95	64	128	0.665
EMA	2x2	0.4	0.95	68	136	0.663

EMA	2x2	0.4	0.95	76	152	0.749
EMA	2x2	0.4	0.95	84	168	0.818
EMA	2x2	0.4	0.95	96	192	0.859
EMA	2x2	0.4	0.95	108	216	0.908
EMA	2x2	0.4	0.95	120	240	0.932
EMA	2x2	0.4	0.95	132	264	0.95
EMA	2x2	0.4	0.95	144	288	0.97
EMA	2x2	0.4	0.95	160	320	0.981
EMA	2x2	0.4	1	12	24	0.007
EMA	2x2	0.4	1	24	48	0.07
EMA	2x2	0.4	1	36	72	0.29
EMA	2x2	0.4	1	40	80	0.385
EMA	2x2	0.4	1	48	96	0.521
EMA	2x2	0.4	1	56	112	0.65
EMA	2x2	0.4	1	64	128	0.737
EMA	2x2	0.4	1	68	136	0.787
EMA	2x2	0.4	1	76	152	0.809
EMA	2x2	0.4	1	84	168	0.866
EMA	2x2	0.4	1	96	192	0.924
EMA	2x2	0.4	1	108	216	0.944
EMA	2x2	0.4	1	120	240	0.971
EMA	2x2	0.4	1	132	264	0.978
EMA	2x2	0.4	1	144	288	0.985
EMA	2x2	0.4	1	160	320	0.994
EMA	2x2	0.4	1.05	12	24	0.005
EMA	2x2	0.4	1.05	24	48	0.053
EMA	2x2	0.4	1.05	36	72	0.268
EMA	2x2	0.4	1.05	40	80	0.336
EMA	2x2	0.4	1.05	44	88	0.397
EMA	2x2	0.4	1.05	52	104	0.51
EMA	2x2	0.4	1.05	60	120	0.574
EMA	2x2	0.4	1.05	64	128	0.661
EMA	2x2	0.4	1.05	68	136	0.66
EMA	2x2	0.4	1.05	76	152	0.735
EMA	2x2	0.4	1.05	84	168	0.774
EMA	2x2	0.4	1.05	96	192	0.84
EMA	2x2	0.4	1.05	108	216	0.882
EMA	2x2	0.4	1.05	120	240	0.891
EMA	2x2	0.4	1.05	132	264	0.934
EMA	2x2	0.4	1.05	144	288	0.945
EMA	2x2	0.4	1.05	160	320	0.962

EMA	2x2	0.4	1.1	12	24	0.005
EMA	2x2	0.4	1.1	24	48	0.056
EMA	2x2	0.4	1.1	36	72	0.197
EMA	2x2	0.4	1.1	48	96	0.336
EMA	2x2	0.4	1.1	56	112	0.389
EMA	2x2	0.4	1.1	64	128	0.457
EMA	2x2	0.4	1.1	72	144	0.519
EMA	2x2	0.4	1.1	80	160	0.531
EMA	2x2	0.4	1.1	88	176	0.593
EMA	2x2	0.4	1.1	96	192	0.606
EMA	2x2	0.4	1.1	108	216	0.683
EMA	2x2	0.4	1.1	120	240	0.713
EMA	2x2	0.4	1.1	132	264	0.756
EMA	2x2	0.4	1.1	144	288	0.782
EMA	2x2	0.4	1.1	160	320	0.804
EMA	2x2	0.5	0.9	12	24	0
EMA	2x2	0.5	0.9	24	48	0.012
EMA	2x2	0.5	0.9	36	72	0.08
EMA	2x2	0.5	0.9	48	96	0.204
EMA	2x2	0.5	0.9	56	112	0.284
EMA	2x2	0.5	0.9	64	128	0.364
EMA	2x2	0.5	0.9	72	144	0.403
EMA	2x2	0.5	0.9	80	160	0.437
EMA	2x2	0.5	0.9	88	176	0.472
EMA	2x2	0.5	0.9	96	192	0.535
EMA	2x2	0.5	0.9	108	216	0.581
EMA	2x2	0.5	0.9	120	240	0.619
EMA	2x2	0.5	0.9	132	264	0.679
EMA	2x2	0.5	0.9	144	288	0.694
EMA	2x2	0.5	0.9	160	320	0.752
EMA	2x2	0.5	0.95	12	24	0
EMA	2x2	0.5	0.95	24	48	0.013
EMA	2x2	0.5	0.95	36	72	0.13
EMA	2x2	0.5	0.95	44	88	0.252
EMA	2x2	0.5	0.95	52	104	0.364
EMA	2x2	0.5	0.95	56	112	0.408
EMA	2x2	0.5	0.95	64	128	0.49
EMA	2x2	0.5	0.95	72	144	0.581
EMA	2x2	0.5	0.95	80	160	0.65
EMA	2x2	0.5	0.95	88	176	0.737
EMA	2x2	0.5	0.95	96	192	0.772

EMA	2x2	0.5	0.95	108	216	0.821
EMA	2x2	0.5	0.95	120	240	0.862
EMA	2x2	0.5	0.95	132	264	0.889
EMA	2x2	0.5	0.95	144	288	0.924
EMA	2x2	0.5	0.95	160	320	0.946
EMA	2x2	0.5	1	12	24	0.004
EMA	2x2	0.5	1	24	48	0.022
EMA	2x2	0.5	1	36	72	0.11
EMA	2x2	0.5	1	40	80	0.174
EMA	2x2	0.5	1	48	96	0.298
EMA	2x2	0.5	1	52	104	0.378
EMA	2x2	0.5	1	56	112	0.41
EMA	2x2	0.5	1	64	128	0.566
EMA	2x2	0.5	1	72	144	0.654
EMA	2x2	0.5	1	80	160	0.712
EMA	2x2	0.5	1	88	176	0.752
EMA	2x2	0.5	1	96	192	0.8
EMA	2x2	0.5	1	108	216	0.862
EMA	2x2	0.5	1	120	240	0.911
EMA	2x2	0.5	1	132	264	0.937
EMA	2x2	0.5	1	144	288	0.954
EMA	2x2	0.5	1	160	320	0.976
EMA	2x2	0.5	1.05	12	24	0.002
EMA	2x2	0.5	1.05	24	48	0.011
EMA	2x2	0.5	1.05	36	72	0.121
EMA	2x2	0.5	1.05	44	88	0.241
EMA	2x2	0.5	1.05	48	96	0.291
EMA	2x2	0.5	1.05	56	112	0.387
EMA	2x2	0.5	1.05	64	128	0.463
EMA	2x2	0.5	1.05	72	144	0.56
EMA	2x2	0.5	1.05	80	160	0.631
EMA	2x2	0.5	1.05	88	176	0.684
EMA	2x2	0.5	1.05	96	192	0.72
EMA	2x2	0.5	1.05	108	216	0.759
EMA	2x2	0.5	1.05	120	240	0.835
EMA	2x2	0.5	1.05	132	264	0.859
EMA	2x2	0.5	1.05	144	288	0.865
EMA	2x2	0.5	1.05	160	320	0.911
EMA	2x2	0.5	1.1	12	24	0.002
EMA	2x2	0.5	1.1	24	48	0.008
EMA	2x2	0.5	1.1	36	72	0.11

EMA	2x2	0.5	1.1	44	88	0.202
EMA	2x2	0.5	1.1	56	112	0.298
EMA	2x2	0.5	1.1	68	136	0.376
EMA	2x2	0.5	1.1	72	144	0.414
EMA	2x2	0.5	1.1	76	152	0.437
EMA	2x2	0.5	1.1	80	160	0.45
EMA	2x2	0.5	1.1	88	176	0.505
EMA	2x2	0.5	1.1	96	192	0.525
EMA	2x2	0.5	1.1	108	216	0.57
EMA	2x2	0.5	1.1	120	240	0.598
EMA	2x2	0.5	1.1	132	264	0.653
EMA	2x2	0.5	1.1	144	288	0.718
EMA	2x2	0.5	1.1	160	320	0.739
EMA	2x2	0.6	0.9	12	24	0
EMA	2x2	0.6	0.9	20	40	0.001
EMA	2x2	0.6	0.9	28	56	0.004
EMA	2x2	0.6	0.9	36	72	0.034
EMA	2x2	0.6	0.9	44	88	0.076
EMA	2x2	0.6	0.9	52	104	0.126
EMA	2x2	0.6	0.9	64	128	0.275
EMA	2x2	0.6	0.9	72	144	0.306
EMA	2x2	0.6	0.9	80	160	0.37
EMA	2x2	0.6	0.9	92	184	0.429
EMA	2x2	0.6	0.9	100	200	0.443
EMA	2x2	0.6	0.9	108	216	0.489
EMA	2x2	0.6	0.9	120	240	0.528
EMA	2x2	0.6	0.9	132	264	0.611
EMA	2x2	0.6	0.9	144	288	0.597
EMA	2x2	0.6	0.9	160	320	0.652
EMA	2x2	0.6	0.95	12	24	0
EMA	2x2	0.6	0.95	20	40	0.004
EMA	2x2	0.6	0.95	28	56	0.012
EMA	2x2	0.6	0.95	36	72	0.045
EMA	2x2	0.6	0.95	44	88	0.094
EMA	2x2	0.6	0.95	52	104	0.197
EMA	2x2	0.6	0.95	60	120	0.283
EMA	2x2	0.6	0.95	64	128	0.322
EMA	2x2	0.6	0.95	72	144	0.439
EMA	2x2	0.6	0.95	80	160	0.495
EMA	2x2	0.6	0.95	92	184	0.578
EMA	2x2	0.6	0.95	100	200	0.64

EMA	2x2	0.6	0.95	108	216	0.695
EMA	2x2	0.6	0.95	120	240	0.774
EMA	2x2	0.6	0.95	132	264	0.806
EMA	2x2	0.6	0.95	144	288	0.824
EMA	2x2	0.6	0.95	160	320	0.845
EMA	2x2	0.6	1	12	24	0
EMA	2x2	0.6	1	20	40	0.004
EMA	2x2	0.6	1	28	56	0.009
EMA	2x2	0.6	1	36	72	0.034
EMA	2x2	0.6	1	44	88	0.114
EMA	2x2	0.6	1	48	96	0.165
EMA	2x2	0.6	1	52	104	0.214
EMA	2x2	0.6	1	60	120	0.329
EMA	2x2	0.6	1	72	144	0.482
EMA	2x2	0.6	1	80	160	0.559
EMA	2x2	0.6	1	92	184	0.658
EMA	2x2	0.6	1	100	200	0.714
EMA	2x2	0.6	1	108	216	0.763
EMA	2x2	0.6	1	120	240	0.815
EMA	2x2	0.6	1	132	264	0.864
EMA	2x2	0.6	1	144	288	0.889
EMA	2x2	0.6	1	160	320	0.93
EMA	2x2	0.6	1.05	12	24	0
EMA	2x2	0.6	1.05	20	40	0.004
EMA	2x2	0.6	1.05	28	56	0.015
EMA	2x2	0.6	1.05	36	72	0.038
EMA	2x2	0.6	1.05	44	88	0.105
EMA	2x2	0.6	1.05	48	96	0.14
EMA	2x2	0.6	1.05	52	104	0.207
EMA	2x2	0.6	1.05	64	128	0.334
EMA	2x2	0.6	1.05	72	144	0.445
EMA	2x2	0.6	1.05	80	160	0.501
EMA	2x2	0.6	1.05	92	184	0.579
EMA	2x2	0.6	1.05	100	200	0.633
EMA	2x2	0.6	1.05	108	216	0.682
EMA	2x2	0.6	1.05	120	240	0.743
EMA	2x2	0.6	1.05	132	264	0.761
EMA	2x2	0.6	1.05	144	288	0.805
EMA	2x2	0.6	1.05	160	320	0.847
EMA	2x2	0.6	1.1	12	24	0
EMA	2x2	0.6	1.1	24	48	0.002

EMA	2x2	0.6	1.1	36	72	0.037
EMA	2x2	0.6	1.1	48	96	0.119
EMA	2x2	0.6	1.1	60	120	0.233
EMA	2x2	0.6	1.1	64	128	0.277
EMA	2x2	0.6	1.1	72	144	0.325
EMA	2x2	0.6	1.1	84	168	0.407
EMA	2x2	0.6	1.1	92	184	0.436
EMA	2x2	0.6	1.1	100	200	0.451
EMA	2x2	0.6	1.1	108	216	0.494
EMA	2x2	0.6	1.1	120	240	0.5
EMA	2x2	0.6	1.1	132	264	0.58
EMA	2x2	0.6	1.1	144	288	0.614
EMA	2x2	0.6	1.1	160	320	0.637
EMA	3x3	0.2	0.9	9	27	0.07
EMA	3x3	0.2	0.9	12	36	0.168
EMA	3x3	0.2	0.9	15	45	0.279
EMA	3x3	0.2	0.9	18	54	0.363
EMA	3x3	0.2	0.9	21	63	0.428
EMA	3x3	0.2	0.9	24	72	0.49
EMA	3x3	0.2	0.9	27	81	0.5
EMA	3x3	0.2	0.9	33	99	0.63
EMA	3x3	0.2	0.9	39	117	0.644
EMA	3x3	0.2	0.9	45	135	0.72
EMA	3x3	0.2	0.9	54	162	0.811
EMA	3x3	0.2	0.9	60	180	0.811
EMA	3x3	0.2	0.9	66	198	0.848
EMA	3x3	0.2	0.9	75	225	0.897
EMA	3x3	0.2	0.9	81	243	0.896
EMA	3x3	0.2	0.9	90	270	0.932
EMA	3x3	0.2	0.9	96	288	0.943
EMA	3x3	0.2	0.9	105	315	0.963
EMA	3x3	0.2	0.95	9	27	0.093
EMA	3x3	0.2	0.95	12	36	0.225
EMA	3x3	0.2	0.95	15	45	0.363
EMA	3x3	0.2	0.95	18	54	0.475
EMA	3x3	0.2	0.95	21	63	0.592
EMA	3x3	0.2	0.95	24	72	0.665
EMA	3x3	0.2	0.95	27	81	0.705
EMA	3x3	0.2	0.95	33	99	0.824
EMA	3x3	0.2	0.95	39	117	0.893
EMA	3x3	0.2	0.95	45	135	0.921

EMA	3x3	0.2	0.95	54	162	0.962
EMA	3x3	0.2	0.95	60	180	0.97
EMA	3x3	0.2	0.95	66	198	0.986
EMA	3x3	0.2	0.95	75	225	0.984
EMA	3x3	0.2	0.95	81	243	0.995
EMA	3x3	0.2	0.95	90	270	0.996
EMA	3x3	0.2	0.95	96	288	0.997
EMA	3x3	0.2	0.95	105	315	0.997
EMA	3x3	0.2	1	9	27	0.105
EMA	3x3	0.2	1	12	36	0.254
EMA	3x3	0.2	1	15	45	0.413
EMA	3x3	0.2	1	18	54	0.529
EMA	3x3	0.2	1	24	72	0.716
EMA	3x3	0.2	1	27	81	0.81
EMA	3x3	0.2	1	33	99	0.875
EMA	3x3	0.2	1	39	117	0.92
EMA	3x3	0.2	1	45	135	0.961
EMA	3x3	0.2	1	54	162	0.979
EMA	3x3	0.2	1	60	180	0.988
EMA	3x3	0.2	1	66	198	0.992
EMA	3x3	0.2	1	75	225	0.997
EMA	3x3	0.2	1	81	243	0.997
EMA	3x3	0.2	1	90	270	1
EMA	3x3	0.2	1	96	288	0.999
EMA	3x3	0.2	1	105	315	1
EMA	3x3	0.2	1.05	9	27	0.102
EMA	3x3	0.2	1.05	12	36	0.231
EMA	3x3	0.2	1.05	15	45	0.355
EMA	3x3	0.2	1.05	18	54	0.466
EMA	3x3	0.2	1.05	21	63	0.558
EMA	3x3	0.2	1.05	24	72	0.624
EMA	3x3	0.2	1.05	27	81	0.681
EMA	3x3	0.2	1.05	33	99	0.766
EMA	3x3	0.2	1.05	39	117	0.864
EMA	3x3	0.2	1.05	45	135	0.893
EMA	3x3	0.2	1.05	54	162	0.931
EMA	3x3	0.2	1.05	60	180	0.946
EMA	3x3	0.2	1.05	66	198	0.971
EMA	3x3	0.2	1.05	75	225	0.971
EMA	3x3	0.2	1.05	81	243	0.985
EMA	3x3	0.2	1.05	90	270	0.991

EMA	3x3	0.2	1.05	96	288	0.995
EMA	3x3	0.2	1.05	105	315	0.996
EMA	3x3	0.2	1.1	9	27	0.081
EMA	3x3	0.2	1.1	12	36	0.171
EMA	3x3	0.2	1.1	15	45	0.289
EMA	3x3	0.2	1.1	18	54	0.365
EMA	3x3	0.2	1.1	21	63	0.427
EMA	3x3	0.2	1.1	24	72	0.448
EMA	3x3	0.2	1.1	27	81	0.502
EMA	3x3	0.2	1.1	30	90	0.558
EMA	3x3	0.2	1.1	39	117	0.65
EMA	3x3	0.2	1.1	45	135	0.708
EMA	3x3	0.2	1.1	54	162	0.76
EMA	3x3	0.2	1.1	60	180	0.804
EMA	3x3	0.2	1.1	66	198	0.826
EMA	3x3	0.2	1.1	75	225	0.872
EMA	3x3	0.2	1.1	81	243	0.887
EMA	3x3	0.2	1.1	90	270	0.92
EMA	3x3	0.2	1.1	96	288	0.942
EMA	3x3	0.2	1.1	105	315	0.953
EMA	3x3	0.3	0.9	9	27	0.017
EMA	3x3	0.3	0.9	15	45	0.141
EMA	3x3	0.3	0.9	24	72	0.345
EMA	3x3	0.3	0.9	33	99	0.485
EMA	3x3	0.3	0.9	39	117	0.54
EMA	3x3	0.3	0.9	48	144	0.627
EMA	3x3	0.3	0.9	54	162	0.682
EMA	3x3	0.3	0.9	60	180	0.728
EMA	3x3	0.3	0.9	66	198	0.734
EMA	3x3	0.3	0.9	75	225	0.762
EMA	3x3	0.3	0.9	81	243	0.812
EMA	3x3	0.3	0.9	90	270	0.842
EMA	3x3	0.3	0.9	96	288	0.839
EMA	3x3	0.3	0.9	105	315	0.896
EMA	3x3	0.3	0.95	9	27	0.026
EMA	3x3	0.3	0.95	12	36	0.064
EMA	3x3	0.3	0.95	15	45	0.186
EMA	3x3	0.3	0.95	18	54	0.295
EMA	3x3	0.3	0.95	21	63	0.376
EMA	3x3	0.3	0.95	24	72	0.48
EMA	3x3	0.3	0.95	27	81	0.55

EMA	3x3	0.3	0.95	36	108	0.726
EMA	3x3	0.3	0.95	39	117	0.756
EMA	3x3	0.3	0.95	48	144	0.857
EMA	3x3	0.3	0.95	54	162	0.874
EMA	3x3	0.3	0.95	60	180	0.908
EMA	3x3	0.3	0.95	66	198	0.936
EMA	3x3	0.3	0.95	75	225	0.966
EMA	3x3	0.3	0.95	81	243	0.962
EMA	3x3	0.3	0.95	90	270	0.979
EMA	3x3	0.3	0.95	96	288	0.987
EMA	3x3	0.3	0.95	105	315	0.995
EMA	3x3	0.3	1	9	27	0.018
EMA	3x3	0.3	1	12	36	0.089
EMA	3x3	0.3	1	15	45	0.206
EMA	3x3	0.3	1	18	54	0.321
EMA	3x3	0.3	1	21	63	0.43
EMA	3x3	0.3	1	24	72	0.507
EMA	3x3	0.3	1	27	81	0.628
EMA	3x3	0.3	1	30	90	0.689
EMA	3x3	0.3	1	39	117	0.785
EMA	3x3	0.3	1	48	144	0.912
EMA	3x3	0.3	1	54	162	0.926
EMA	3x3	0.3	1	60	180	0.966
EMA	3x3	0.3	1	66	198	0.977
EMA	3x3	0.3	1	75	225	0.989
EMA	3x3	0.3	1	81	243	0.994
EMA	3x3	0.3	1	90	270	0.995
EMA	3x3	0.3	1	96	288	0.998
EMA	3x3	0.3	1	105	315	0.997
EMA	3x3	0.3	1.05	9	27	0.028
EMA	3x3	0.3	1.05	12	36	0.068
EMA	3x3	0.3	1.05	15	45	0.175
EMA	3x3	0.3	1.05	18	54	0.298
EMA	3x3	0.3	1.05	21	63	0.363
EMA	3x3	0.3	1.05	24	72	0.481
EMA	3x3	0.3	1.05	27	81	0.534
EMA	3x3	0.3	1.05	33	99	0.683
EMA	3x3	0.3	1.05	36	108	0.687
EMA	3x3	0.3	1.05	39	117	0.715
EMA	3x3	0.3	1.05	48	144	0.803
EMA	3x3	0.3	1.05	54	162	0.867

EMA	3x3	0.3	1.05	60	180	0.892
EMA	3x3	0.3	1.05	66	198	0.923
EMA	3x3	0.3	1.05	75	225	0.942
EMA	3x3	0.3	1.05	81	243	0.958
EMA	3x3	0.3	1.05	90	270	0.974
EMA	3x3	0.3	1.05	96	288	0.977
EMA	3x3	0.3	1.05	105	315	0.986
EMA	3x3	0.3	1.1	9	27	0.015
EMA	3x3	0.3	1.1	15	45	0.148
EMA	3x3	0.3	1.1	24	72	0.373
EMA	3x3	0.3	1.1	33	99	0.501
EMA	3x3	0.3	1.1	39	117	0.559
EMA	3x3	0.3	1.1	45	135	0.616
EMA	3x3	0.3	1.1	51	153	0.66
EMA	3x3	0.3	1.1	51	153	0.623
EMA	3x3	0.3	1.1	63	189	0.726
EMA	3x3	0.3	1.1	66	198	0.742
EMA	3x3	0.3	1.1	48	144	0.616
EMA	3x3	0.3	1.1	54	162	0.654
EMA	3x3	0.3	1.1	60	180	0.715
EMA	3x3	0.3	1.1	66	198	0.743
EMA	3x3	0.3	1.1	75	225	0.781
EMA	3x3	0.3	1.1	81	243	0.815
EMA	3x3	0.3	1.1	90	270	0.842
EMA	3x3	0.3	1.1	96	288	0.876
EMA	3x3	0.3	1.1	105	315	0.894
EMA	3x3	0.4	0.9	9	27	0.004
EMA	3x3	0.4	0.9	15	45	0.048
EMA	3x3	0.4	0.9	24	72	0.248
EMA	3x3	0.4	0.9	33	99	0.416
EMA	3x3	0.4	0.9	39	117	0.443
EMA	3x3	0.4	0.9	42	126	0.482
EMA	3x3	0.4	0.9	45	135	0.53
EMA	3x3	0.4	0.9	51	153	0.526
EMA	3x3	0.4	0.9	54	162	0.593
EMA	3x3	0.4	0.9	63	189	0.623
EMA	3x3	0.4	0.9	72	216	0.702
EMA	3x3	0.4	0.9	81	243	0.734
EMA	3x3	0.4	0.9	90	270	0.765
EMA	3x3	0.4	0.9	96	288	0.773
EMA	3x3	0.4	0.9	105	315	0.816

EMA	3x3	0.4	0.95	9	27	0.004
EMA	3x3	0.4	0.95	15	45	0.048
EMA	3x3	0.4	0.95	24	72	0.31
EMA	3x3	0.4	0.95	27	81	0.393
EMA	3x3	0.4	0.95	30	90	0.479
EMA	3x3	0.4	0.95	36	108	0.603
EMA	3x3	0.4	0.95	39	117	0.639
EMA	3x3	0.4	0.95	42	126	0.664
EMA	3x3	0.4	0.95	45	135	0.729
EMA	3x3	0.4	0.95	51	153	0.767
EMA	3x3	0.4	0.95	54	162	0.795
EMA	3x3	0.4	0.95	63	189	0.847
EMA	3x3	0.4	0.95	72	216	0.886
EMA	3x3	0.4	0.95	81	243	0.919
EMA	3x3	0.4	0.95	90	270	0.944
EMA	3x3	0.4	0.95	96	288	0.965
EMA	3x3	0.4	0.95	105	315	0.972
EMA	3x3	0.4	1	9	27	0.007
EMA	3x3	0.4	1	15	45	0.055
EMA	3x3	0.4	1	24	72	0.364
EMA	3x3	0.4	1	27	81	0.431
EMA	3x3	0.4	1	33	99	0.586
EMA	3x3	0.4	1	39	117	0.714
EMA	3x3	0.4	1	42	126	0.756
EMA	3x3	0.4	1	45	135	0.793
EMA	3x3	0.4	1	51	153	0.825
EMA	3x3	0.4	1	54	162	0.877
EMA	3x3	0.4	1	63	189	0.929
EMA	3x3	0.4	1	72	216	0.961
EMA	3x3	0.4	1	81	243	0.979
EMA	3x3	0.4	1	90	270	0.989
EMA	3x3	0.4	1	96	288	0.992
EMA	3x3	0.4	1	105	315	0.996
EMA	3x3	0.4	1.05	9	27	0.002
EMA	3x3	0.4	1.05	15	45	0.07
EMA	3x3	0.4	1.05	24	72	0.314
EMA	3x3	0.4	1.05	27	81	0.399
EMA	3x3	0.4	1.05	30	90	0.455
EMA	3x3	0.4	1.05	36	108	0.61
EMA	3x3	0.4	1.05	39	117	0.666
EMA	3x3	0.4	1.05	42	126	0.663

EMA	3x3	0.4	1.05	45	135	0.692
EMA	3x3	0.4	1.05	51	153	0.784
EMA	3x3	0.4	1.05	54	162	0.799
EMA	3x3	0.4	1.05	63	189	0.844
EMA	3x3	0.4	1.05	72	216	0.899
EMA	3x3	0.4	1.05	81	243	0.926
EMA	3x3	0.4	1.05	90	270	0.957
EMA	3x3	0.4	1.05	96	288	0.953
EMA	3x3	0.4	1.05	105	315	0.975
EMA	3x3	0.4	1.1	9	27	0.003
EMA	3x3	0.4	1.1	15	45	0.064
EMA	3x3	0.4	1.1	24	72	0.255
EMA	3x3	0.4	1.1	33	99	0.432
EMA	3x3	0.4	1.1	39	117	0.498
EMA	3x3	0.4	1.1	42	126	0.529
EMA	3x3	0.4	1.1	48	144	0.58
EMA	3x3	0.4	1.1	54	162	0.636
EMA	3x3	0.4	1.1	57	171	0.66
EMA	3x3	0.4	1.1	63	189	0.667
EMA	3x3	0.4	1.1	72	216	0.731
EMA	3x3	0.4	1.1	81	243	0.801
EMA	3x3	0.4	1.1	90	270	0.812
EMA	3x3	0.4	1.1	96	288	0.835
EMA	3x3	0.4	1.1	105	315	0.86
EMA	3x3	0.5	0.9	9	27	0
EMA	3x3	0.5	0.9	15	45	0.013
EMA	3x3	0.5	0.9	24	72	0.136
EMA	3x3	0.5	0.9	33	99	0.287
EMA	3x3	0.5	0.9	39	117	0.376
EMA	3x3	0.5	0.9	42	126	0.376
EMA	3x3	0.5	0.9	48	144	0.423
EMA	3x3	0.5	0.9	54	162	0.477
EMA	3x3	0.5	0.9	57	171	0.484
EMA	3x3	0.5	0.9	63	189	0.573
EMA	3x3	0.5	0.9	72	216	0.578
EMA	3x3	0.5	0.9	81	243	0.626
EMA	3x3	0.5	0.9	90	270	0.667
EMA	3x3	0.5	0.9	96	288	0.696
EMA	3x3	0.5	0.9	105	315	0.724
EMA	3x3	0.5	0.95	9	27	0.003
EMA	3x3	0.5	0.95	15	45	0.019

EMA	3x3	0.5	0.95	24	72	0.146
EMA	3x3	0.5	0.95	30	90	0.294
EMA	3x3	0.5	0.95	36	108	0.451
EMA	3x3	0.5	0.95	39	117	0.464
EMA	3x3	0.5	0.95	42	126	0.531
EMA	3x3	0.5	0.95	48	144	0.571
EMA	3x3	0.5	0.95	54	162	0.663
EMA	3x3	0.5	0.95	57	171	0.698
EMA	3x3	0.5	0.95	63	189	0.745
EMA	3x3	0.5	0.95	72	216	0.792
EMA	3x3	0.5	0.95	81	243	0.821
EMA	3x3	0.5	0.95	90	270	0.897
EMA	3x3	0.5	0.95	96	288	0.904
EMA	3x3	0.5	0.95	105	315	0.923
EMA	3x3	0.5	1	9	27	0
EMA	3x3	0.5	1	15	45	0.018
EMA	3x3	0.5	1	24	72	0.169
EMA	3x3	0.5	1	27	81	0.25
EMA	3x3	0.5	1	33	99	0.423
EMA	3x3	0.5	1	36	108	0.465
EMA	3x3	0.5	1	39	117	0.521
EMA	3x3	0.5	1	42	126	0.595
EMA	3x3	0.5	1	48	144	0.681
EMA	3x3	0.5	1	54	162	0.753
EMA	3x3	0.5	1	57	171	0.8
EMA	3x3	0.5	1	63	189	0.822
EMA	3x3	0.5	1	72	216	0.895
EMA	3x3	0.5	1	81	243	0.917
EMA	3x3	0.5	1	90	270	0.956
EMA	3x3	0.5	1	96	288	0.965
EMA	3x3	0.5	1	105	315	0.964
EMA	3x3	0.5	1.05	9	27	0.001
EMA	3x3	0.5	1.05	15	45	0.014
EMA	3x3	0.5	1.05	24	72	0.186
EMA	3x3	0.5	1.05	30	90	0.322
EMA	3x3	0.5	1.05	33	99	0.357
EMA	3x3	0.5	1.05	39	117	0.469
EMA	3x3	0.5	1.05	42	126	0.537
EMA	3x3	0.5	1.05	48	144	0.63
EMA	3x3	0.5	1.05	54	162	0.672
EMA	3x3	0.5	1.05	57	171	0.731

EMA	3x3	0.5	1.05	63	189	0.751
EMA	3x3	0.5	1.05	72	216	0.815
EMA	3x3	0.5	1.05	81	243	0.864
EMA	3x3	0.5	1.05	90	270	0.881
EMA	3x3	0.5	1.05	96	288	0.926
EMA	3x3	0.5	1.05	105	315	0.922
EMA	3x3	0.5	1.1	9	27	0.003
EMA	3x3	0.5	1.1	15	45	0.017
EMA	3x3	0.5	1.1	24	72	0.139
EMA	3x3	0.5	1.1	30	90	0.253
EMA	3x3	0.5	1.1	39	117	0.384
EMA	3x3	0.5	1.1	45	135	0.443
EMA	3x3	0.5	1.1	48	144	0.508
EMA	3x3	0.5	1.1	51	153	0.495
EMA	3x3	0.5	1.1	54	162	0.545
EMA	3x3	0.5	1.1	57	171	0.558
EMA	3x3	0.5	1.1	63	189	0.619
EMA	3x3	0.5	1.1	72	216	0.639
EMA	3x3	0.5	1.1	81	243	0.694
EMA	3x3	0.5	1.1	90	270	0.717
EMA	3x3	0.5	1.1	96	288	0.744
EMA	3x3	0.5	1.1	105	315	0.768
EMA	3x3	0.6	0.9	9	27	0
EMA	3x3	0.6	0.9	15	45	0.002
EMA	3x3	0.6	0.9	18	54	0.009
EMA	3x3	0.6	0.9	24	72	0.05
EMA	3x3	0.6	0.9	30	90	0.125
EMA	3x3	0.6	0.9	36	108	0.216
EMA	3x3	0.6	0.9	42	126	0.276
EMA	3x3	0.6	0.9	48	144	0.329
EMA	3x3	0.6	0.9	54	162	0.399
EMA	3x3	0.6	0.9	60	180	0.414
EMA	3x3	0.6	0.9	66	198	0.473
EMA	3x3	0.6	0.9	72	216	0.448
EMA	3x3	0.6	0.9	81	243	0.555
EMA	3x3	0.6	0.9	90	270	0.556
EMA	3x3	0.6	0.9	96	288	0.616
EMA	3x3	0.6	0.9	105	315	0.638
EMA	3x3	0.6	0.95	9	27	0
EMA	3x3	0.6	0.95	15	45	0.004
EMA	3x3	0.6	0.95	18	54	0.011

EMA	3x3	0.6	0.95	24	72	0.053
EMA	3x3	0.6	0.95	30	90	0.175
EMA	3x3	0.6	0.95	36	108	0.27
EMA	3x3	0.6	0.95	39	117	0.325
EMA	3x3	0.6	0.95	45	135	0.445
EMA	3x3	0.6	0.95	48	144	0.49
EMA	3x3	0.6	0.95	54	162	0.532
EMA	3x3	0.6	0.95	60	180	0.597
EMA	3x3	0.6	0.95	66	198	0.669
EMA	3x3	0.6	0.95	72	216	0.683
EMA	3x3	0.6	0.95	81	243	0.765
EMA	3x3	0.6	0.95	90	270	0.766
EMA	3x3	0.6	0.95	96	288	0.808
EMA	3x3	0.6	0.95	105	315	0.858
EMA	3x3	0.6	1	9	27	0
EMA	3x3	0.6	1	15	45	0.003
EMA	3x3	0.6	1	18	54	0.02
EMA	3x3	0.6	1	24	72	0.059
EMA	3x3	0.6	1	30	90	0.148
EMA	3x3	0.6	1	33	99	0.233
EMA	3x3	0.6	1	36	108	0.3
EMA	3x3	0.6	1	42	126	0.41
EMA	3x3	0.6	1	48	144	0.496
EMA	3x3	0.6	1	54	162	0.584
EMA	3x3	0.6	1	60	180	0.659
EMA	3x3	0.6	1	66	198	0.72
EMA	3x3	0.6	1	72	216	0.786
EMA	3x3	0.6	1	81	243	0.824
EMA	3x3	0.6	1	90	270	0.879
EMA	3x3	0.6	1	96	288	0.881
EMA	3x3	0.6	1	105	315	0.906
EMA	3x3	0.6	1.05	9	27	0
EMA	3x3	0.6	1.05	15	45	0.002
EMA	3x3	0.6	1.05	18	54	0.013
EMA	3x3	0.6	1.05	24	72	0.072
EMA	3x3	0.6	1.05	30	90	0.182
EMA	3x3	0.6	1.05	33	99	0.228
EMA	3x3	0.6	1.05	36	108	0.277
EMA	3x3	0.6	1.05	42	126	0.37
EMA	3x3	0.6	1.05	48	144	0.477
EMA	3x3	0.6	1.05	54	162	0.563

EMA	3x3	0.6	1.05	60	180	0.62
EMA	3x3	0.6	1.05	66	198	0.664
EMA	3x3	0.6	1.05	72	216	0.714
EMA	3x3	0.6	1.05	81	243	0.77
EMA	3x3	0.6	1.05	90	270	0.817
EMA	3x3	0.6	1.05	96	288	0.826
EMA	3x3	0.6	1.05	105	315	0.877
EMA	3x3	0.6	1.1	9	27	0.001
EMA	3x3	0.6	1.1	15	45	0.003
EMA	3x3	0.6	1.1	24	72	0.038
EMA	3x3	0.6	1.1	30	90	0.16
EMA	3x3	0.6	1.1	39	117	0.295
EMA	3x3	0.6	1.1	42	126	0.298
EMA	3x3	0.6	1.1	45	135	0.349
EMA	3x3	0.6	1.1	54	162	0.418
EMA	3x3	0.6	1.1	60	180	0.487
EMA	3x3	0.6	1.1	66	198	0.528
EMA	3x3	0.6	1.1	72	216	0.565
EMA	3x3	0.6	1.1	81	243	0.6
EMA	3x3	0.6	1.1	90	270	0.637
EMA	3x3	0.6	1.1	96	288	0.647
EMA	3x3	0.6	1.1	105	315	0.709
EMA	2x4	0.2	0.9	6	24	0.083
EMA	2x4	0.2	0.9	8	32	0.146
EMA	2x4	0.2	0.9	12	48	0.343
EMA	2x4	0.2	0.9	14	56	0.385
EMA	2x4	0.2	0.9	16	64	0.434
EMA	2x4	0.2	0.9	18	72	0.506
EMA	2x4	0.2	0.9	20	80	0.546
EMA	2x4	0.2	0.9	24	96	0.579
EMA	2x4	0.2	0.9	30	120	0.679
EMA	2x4	0.2	0.9	34	136	0.693
EMA	2x4	0.2	0.9	40	160	0.78
EMA	2x4	0.2	0.9	46	184	0.811
EMA	2x4	0.2	0.9	50	200	0.85
EMA	2x4	0.2	0.9	54	216	0.892
EMA	2x4	0.2	0.9	60	240	0.9
EMA	2x4	0.2	0.9	66	264	0.918
EMA	2x4	0.2	0.9	72	288	0.937
EMA	2x4	0.2	0.9	80	320	0.964
EMA	2x4	0.2	0.95	6	24	0.097

EMA	2x4	0.2	0.95	8	32	0.198
EMA	2x4	0.2	0.95	10	40	0.345
EMA	2x4	0.2	0.95	14	56	0.53
EMA	2x4	0.2	0.95	16	64	0.594
EMA	2x4	0.2	0.95	18	72	0.669
EMA	2x4	0.2	0.95	20	80	0.733
EMA	2x4	0.2	0.95	24	96	0.797
EMA	2x4	0.2	0.95	30	120	0.871
EMA	2x4	0.2	0.95	34	136	0.912
EMA	2x4	0.2	0.95	40	160	0.956
EMA	2x4	0.2	0.95	46	184	0.976
EMA	2x4	0.2	0.95	50	200	0.982
EMA	2x4	0.2	0.95	54	216	0.987
EMA	2x4	0.2	0.95	60	240	0.992
EMA	2x4	0.2	0.95	66	264	0.992
EMA	2x4	0.2	0.95	72	288	0.999
EMA	2x4	0.2	0.95	80	320	0.998
EMA	2x4	0.2	1	6	24	0.099
EMA	2x4	0.2	1	8	32	0.215
EMA	2x4	0.2	1	10	40	0.347
EMA	2x4	0.2	1	14	56	0.56
EMA	2x4	0.2	1	18	72	0.713
EMA	2x4	0.2	1	20	80	0.788
EMA	2x4	0.2	1	24	96	0.869
EMA	2x4	0.2	1	30	120	0.931
EMA	2x4	0.2	1	34	136	0.956
EMA	2x4	0.2	1	40	160	0.984
EMA	2x4	0.2	1	46	184	0.995
EMA	2x4	0.2	1	50	200	0.995
EMA	2x4	0.2	1	54	216	0.995
EMA	2x4	0.2	1	60	240	1
EMA	2x4	0.2	1	66	264	1
EMA	2x4	0.2	1	72	288	0.999
EMA	2x4	0.2	1	80	320	1
EMA	2x4	0.2	1.05	6	24	0.09
EMA	2x4	0.2	1.05	8	32	0.213
EMA	2x4	0.2	1.05	12	48	0.44
EMA	2x4	0.2	1.05	14	56	0.491
EMA	2x4	0.2	1.05	16	64	0.578
EMA	2x4	0.2	1.05	18	72	0.621
EMA	2x4	0.2	1.05	20	80	0.698

EMA	2x4	0.2	1.05	24	96	0.768
EMA	2x4	0.2	1.05	30	120	0.865
EMA	2x4	0.2	1.05	34	136	0.875
EMA	2x4	0.2	1.05	40	160	0.918
EMA	2x4	0.2	1.05	46	184	0.951
EMA	2x4	0.2	1.05	50	200	0.969
EMA	2x4	0.2	1.05	54	216	0.964
EMA	2x4	0.2	1.05	60	240	0.978
EMA	2x4	0.2	1.05	66	264	0.985
EMA	2x4	0.2	1.05	72	288	0.988
EMA	2x4	0.2	1.05	80	320	0.997
EMA	2x4	0.2	1.1	6	24	0.087
EMA	2x4	0.2	1.1	8	32	0.199
EMA	2x4	0.2	1.1	10	40	0.299
EMA	2x4	0.2	1.1	14	56	0.394
EMA	2x4	0.2	1.1	16	64	0.428
EMA	2x4	0.2	1.1	18	72	0.51
EMA	2x4	0.2	1.1	20	80	0.508
EMA	2x4	0.2	1.1	22	88	0.587
EMA	2x4	0.2	1.1	30	120	0.682
EMA	2x4	0.2	1.1	34	136	0.7
EMA	2x4	0.2	1.1	40	160	0.787
EMA	2x4	0.2	1.1	46	184	0.832
EMA	2x4	0.2	1.1	50	200	0.841
EMA	2x4	0.2	1.1	54	216	0.854
EMA	2x4	0.2	1.1	60	240	0.882
EMA	2x4	0.2	1.1	66	264	0.924
EMA	2x4	0.2	1.1	72	288	0.918
EMA	2x4	0.2	1.1	80	320	0.941
EMA	2x4	0.3	0.9	6	24	0.03
EMA	2x4	0.3	0.9	12	48	0.188
EMA	2x4	0.3	0.9	18	72	0.354
EMA	2x4	0.3	0.9	24	96	0.492
EMA	2x4	0.3	0.9	30	120	0.598
EMA	2x4	0.3	0.9	36	144	0.633
EMA	2x4	0.3	0.9	40	160	0.671
EMA	2x4	0.3	0.9	44	176	0.696
EMA	2x4	0.3	0.9	50	200	0.743
EMA	2x4	0.3	0.9	54	216	0.775
EMA	2x4	0.3	0.9	60	240	0.812
EMA	2x4	0.3	0.9	66	264	0.846

EMA	2x4	0.3	0.9	72	288	0.887
EMA	2x4	0.3	0.9	80	320	0.908
EMA	2x4	0.3	0.95	6	24	0.03
EMA	2x4	0.3	0.95	8	32	0.072
EMA	2x4	0.3	0.95	12	48	0.276
EMA	2x4	0.3	0.95	14	56	0.342
EMA	2x4	0.3	0.95	16	64	0.431
EMA	2x4	0.3	0.95	18	72	0.475
EMA	2x4	0.3	0.95	20	80	0.58
EMA	2x4	0.3	0.95	26	104	0.724
EMA	2x4	0.3	0.95	30	120	0.786
EMA	2x4	0.3	0.95	36	144	0.866
EMA	2x4	0.3	0.95	40	160	0.875
EMA	2x4	0.3	0.95	44	176	0.911
EMA	2x4	0.3	0.95	50	200	0.937
EMA	2x4	0.3	0.95	54	216	0.954
EMA	2x4	0.3	0.95	60	240	0.979
EMA	2x4	0.3	0.95	66	264	0.978
EMA	2x4	0.3	0.95	72	288	0.982
EMA	2x4	0.3	0.95	80	320	0.984
EMA	2x4	0.3	1	6	24	0.024
EMA	2x4	0.3	1	8	32	0.081
EMA	2x4	0.3	1	12	48	0.263
EMA	2x4	0.3	1	14	56	0.402
EMA	2x4	0.3	1	16	64	0.473
EMA	2x4	0.3	1	18	72	0.554
EMA	2x4	0.3	1	20	80	0.64
EMA	2x4	0.3	1	22	88	0.684
EMA	2x4	0.3	1	30	120	0.833
EMA	2x4	0.3	1	36	144	0.902
EMA	2x4	0.3	1	40	160	0.939
EMA	2x4	0.3	1	44	176	0.964
EMA	2x4	0.3	1	50	200	0.977
EMA	2x4	0.3	1	54	216	0.988
EMA	2x4	0.3	1	60	240	0.987
EMA	2x4	0.3	1	66	264	0.999
EMA	2x4	0.3	1	72	288	0.998
EMA	2x4	0.3	1	80	320	0.999
EMA	2x4	0.3	1.05	6	24	0.023
EMA	2x4	0.3	1.05	8	32	0.066
EMA	2x4	0.3	1.05	12	48	0.256

EMA	2x4	0.3	1.05	14	56	0.368
EMA	2x4	0.3	1.05	16	64	0.429
EMA	2x4	0.3	1.05	18	72	0.497
EMA	2x4	0.3	1.05	20	80	0.54
EMA	2x4	0.3	1.05	24	96	0.669
EMA	2x4	0.3	1.05	26	104	0.725
EMA	2x4	0.3	1.05	30	120	0.776
EMA	2x4	0.3	1.05	36	144	0.831
EMA	2x4	0.3	1.05	40	160	0.853
EMA	2x4	0.3	1.05	44	176	0.89
EMA	2x4	0.3	1.05	50	200	0.926
EMA	2x4	0.3	1.05	54	216	0.924
EMA	2x4	0.3	1.05	60	240	0.948
EMA	2x4	0.3	1.05	66	264	0.97
EMA	2x4	0.3	1.05	72	288	0.975
EMA	2x4	0.3	1.05	80	320	0.984
EMA	2x4	0.3	1.1	6	24	0.023
EMA	2x4	0.3	1.1	12	48	0.211
EMA	2x4	0.3	1.1	18	72	0.392
EMA	2x4	0.3	1.1	24	96	0.488
EMA	2x4	0.3	1.1	30	120	0.607
EMA	2x4	0.3	1.1	34	136	0.626
EMA	2x4	0.3	1.1	38	152	0.669
EMA	2x4	0.3	1.1	42	168	0.715
EMA	2x4	0.3	1.1	46	184	0.747
EMA	2x4	0.3	1.1	50	200	0.768
EMA	2x4	0.3	1.1	36	144	0.654
EMA	2x4	0.3	1.1	40	160	0.675
EMA	2x4	0.3	1.1	44	176	0.734
EMA	2x4	0.3	1.1	50	200	0.738
EMA	2x4	0.3	1.1	54	216	0.781
EMA	2x4	0.3	1.1	60	240	0.814
EMA	2x4	0.3	1.1	66	264	0.851
EMA	2x4	0.3	1.1	72	288	0.86
EMA	2x4	0.3	1.1	80	320	0.914
EMA	2x4	0.4	0.9	6	24	0.007
EMA	2x4	0.4	0.9	12	48	0.08
EMA	2x4	0.4	0.9	18	72	0.3
EMA	2x4	0.4	0.9	24	96	0.379
EMA	2x4	0.4	0.9	28	112	0.48
EMA	2x4	0.4	0.9	32	128	0.519

EMA	2x4	0.4	0.9	34	136	0.54
EMA	2x4	0.4	0.9	38	152	0.588
EMA	2x4	0.4	0.9	42	168	0.597
EMA	2x4	0.4	0.9	48	192	0.669
EMA	2x4	0.4	0.9	54	216	0.735
EMA	2x4	0.4	0.9	60	240	0.746
EMA	2x4	0.4	0.9	66	264	0.77
EMA	2x4	0.4	0.9	72	288	0.809
EMA	2x4	0.4	0.9	80	320	0.829
EMA	2x4	0.4	0.95	6	24	0.003
EMA	2x4	0.4	0.95	12	48	0.116
EMA	2x4	0.4	0.95	18	72	0.375
EMA	2x4	0.4	0.95	20	80	0.445
EMA	2x4	0.4	0.95	22	88	0.491
EMA	2x4	0.4	0.95	26	104	0.581
EMA	2x4	0.4	0.95	28	112	0.657
EMA	2x4	0.4	0.95	32	128	0.708
EMA	2x4	0.4	0.95	34	136	0.717
EMA	2x4	0.4	0.95	38	152	0.783
EMA	2x4	0.4	0.95	42	168	0.826
EMA	2x4	0.4	0.95	48	192	0.894
EMA	2x4	0.4	0.95	54	216	0.915
EMA	2x4	0.4	0.95	60	240	0.933
EMA	2x4	0.4	0.95	66	264	0.951
EMA	2x4	0.4	0.95	72	288	0.969
EMA	2x4	0.4	0.95	80	320	0.972
EMA	2x4	0.4	1	6	24	0.002
EMA	2x4	0.4	1	12	48	0.141
EMA	2x4	0.4	1	18	72	0.403
EMA	2x4	0.4	1	20	80	0.483
EMA	2x4	0.4	1	24	96	0.613
EMA	2x4	0.4	1	28	112	0.694
EMA	2x4	0.4	1	32	128	0.78
EMA	2x4	0.4	1	34	136	0.82
EMA	2x4	0.4	1	38	152	0.858
EMA	2x4	0.4	1	42	168	0.888
EMA	2x4	0.4	1	48	192	0.943
EMA	2x4	0.4	1	54	216	0.966
EMA	2x4	0.4	1	60	240	0.981
EMA	2x4	0.4	1	66	264	0.988
EMA	2x4	0.4	1	72	288	0.993

EMA	2x4	0.4	1	80	320	0.996
EMA	2x4	0.4	1.05	6	24	0.004
EMA	2x4	0.4	1.05	12	48	0.124
EMA	2x4	0.4	1.05	18	72	0.387
EMA	2x4	0.4	1.05	20	80	0.423
EMA	2x4	0.4	1.05	22	88	0.468
EMA	2x4	0.4	1.05	26	104	0.639
EMA	2x4	0.4	1.05	30	120	0.668
EMA	2x4	0.4	1.05	32	128	0.75
EMA	2x4	0.4	1.05	34	136	0.763
EMA	2x4	0.4	1.05	38	152	0.809
EMA	2x4	0.4	1.05	42	168	0.856
EMA	2x4	0.4	1.05	48	192	0.89
EMA	2x4	0.4	1.05	54	216	0.915
EMA	2x4	0.4	1.05	60	240	0.95
EMA	2x4	0.4	1.05	66	264	0.946
EMA	2x4	0.4	1.05	72	288	0.967
EMA	2x4	0.4	1.05	80	320	0.976
EMA	2x4	0.4	1.1	6	24	0.004
EMA	2x4	0.4	1.1	12	48	0.09
EMA	2x4	0.4	1.1	18	72	0.288
EMA	2x4	0.4	1.1	24	96	0.442
EMA	2x4	0.4	1.1	28	112	0.489
EMA	2x4	0.4	1.1	32	128	0.552
EMA	2x4	0.4	1.1	36	144	0.625
EMA	2x4	0.4	1.1	40	160	0.671
EMA	2x4	0.4	1.1	44	176	0.684
EMA	2x4	0.4	1.1	48	192	0.744
EMA	2x4	0.4	1.1	54	216	0.78
EMA	2x4	0.4	1.1	60	240	0.815
EMA	2x4	0.4	1.1	66	264	0.859
EMA	2x4	0.4	1.1	72	288	0.844
EMA	2x4	0.4	1.1	80	320	0.888
EMA	2x4	0.5	0.9	6	24	0.002
EMA	2x4	0.5	0.9	12	48	0.027
EMA	2x4	0.5	0.9	18	72	0.198
EMA	2x4	0.5	0.9	24	96	0.309
EMA	2x4	0.5	0.9	28	112	0.386
EMA	2x4	0.5	0.9	32	128	0.388
EMA	2x4	0.5	0.9	36	144	0.464
EMA	2x4	0.5	0.9	40	160	0.52

EMA	2x4	0.5	0.9	44	176	0.542
EMA	2x4	0.5	0.9	48	192	0.6
EMA	2x4	0.5	0.9	54	216	0.62
EMA	2x4	0.5	0.9	60	240	0.67
EMA	2x4	0.5	0.9	66	264	0.693
EMA	2x4	0.5	0.9	72	288	0.736
EMA	2x4	0.5	0.9	80	320	0.774
EMA	2x4	0.5	0.95	6	24	0.002
EMA	2x4	0.5	0.95	12	48	0.036
EMA	2x4	0.5	0.95	18	72	0.252
EMA	2x4	0.5	0.95	22	88	0.371
EMA	2x4	0.5	0.95	26	104	0.449
EMA	2x4	0.5	0.95	28	112	0.509
EMA	2x4	0.5	0.95	32	128	0.581
EMA	2x4	0.5	0.95	36	144	0.644
EMA	2x4	0.5	0.95	40	160	0.69
EMA	2x4	0.5	0.95	44	176	0.744
EMA	2x4	0.5	0.95	48	192	0.777
EMA	2x4	0.5	0.95	54	216	0.812
EMA	2x4	0.5	0.95	60	240	0.857
EMA	2x4	0.5	0.95	66	264	0.893
EMA	2x4	0.5	0.95	72	288	0.895
EMA	2x4	0.5	0.95	80	320	0.947
EMA	2x4	0.5	1	6	24	0.005
EMA	2x4	0.5	1	12	48	0.037
EMA	2x4	0.5	1	18	72	0.249
EMA	2x4	0.5	1	20	80	0.308
EMA	2x4	0.5	1	24	96	0.453
EMA	2x4	0.5	1	26	104	0.459
EMA	2x4	0.5	1	28	112	0.543
EMA	2x4	0.5	1	32	128	0.649
EMA	2x4	0.5	1	36	144	0.709
EMA	2x4	0.5	1	40	160	0.777
EMA	2x4	0.5	1	44	176	0.832
EMA	2x4	0.5	1	48	192	0.847
EMA	2x4	0.5	1	54	216	0.89
EMA	2x4	0.5	1	60	240	0.935
EMA	2x4	0.5	1	66	264	0.935
EMA	2x4	0.5	1	72	288	0.967
EMA	2x4	0.5	1	80	320	0.979
EMA	2x4	0.5	1.05	6	24	0.001

EMA	2x4	0.5	1.05	12	48	0.051
EMA	2x4	0.5	1.05	18	72	0.223
EMA	2x4	0.5	1.05	22	88	0.361
EMA	2x4	0.5	1.05	24	96	0.389
EMA	2x4	0.5	1.05	28	112	0.52
EMA	2x4	0.5	1.05	32	128	0.603
EMA	2x4	0.5	1.05	36	144	0.633
EMA	2x4	0.5	1.05	40	160	0.713
EMA	2x4	0.5	1.05	44	176	0.775
EMA	2x4	0.5	1.05	48	192	0.803
EMA	2x4	0.5	1.05	54	216	0.833
EMA	2x4	0.5	1.05	60	240	0.899
EMA	2x4	0.5	1.05	66	264	0.907
EMA	2x4	0.5	1.05	72	288	0.923
EMA	2x4	0.5	1.05	80	320	0.949
EMA	2x4	0.5	1.1	6	24	0.001
EMA	2x4	0.5	1.1	12	48	0.033
EMA	2x4	0.5	1.1	18	72	0.199
EMA	2x4	0.5	1.1	22	88	0.309
EMA	2x4	0.5	1.1	28	112	0.418
EMA	2x4	0.5	1.1	34	136	0.494
EMA	2x4	0.5	1.1	36	144	0.525
EMA	2x4	0.5	1.1	38	152	0.541
EMA	2x4	0.5	1.1	40	160	0.585
EMA	2x4	0.5	1.1	44	176	0.599
EMA	2x4	0.5	1.1	48	192	0.65
EMA	2x4	0.5	1.1	54	216	0.71
EMA	2x4	0.5	1.1	60	240	0.726
EMA	2x4	0.5	1.1	66	264	0.774
EMA	2x4	0.5	1.1	72	288	0.78
EMA	2x4	0.5	1.1	80	320	0.822
EMA	2x4	0.6	0.9	6	24	0
EMA	2x4	0.6	0.9	10	40	0.004
EMA	2x4	0.6	0.9	14	56	0.018
EMA	2x4	0.6	0.9	18	72	0.079
EMA	2x4	0.6	0.9	22	88	0.159
EMA	2x4	0.6	0.9	26	104	0.26
EMA	2x4	0.6	0.9	32	128	0.34
EMA	2x4	0.6	0.9	36	144	0.405
EMA	2x4	0.6	0.9	40	160	0.417
EMA	2x4	0.6	0.9	46	184	0.468

EMA	2x4	0.6	0.9	50	200	0.505
EMA	2x4	0.6	0.9	54	216	0.538
EMA	2x4	0.6	0.9	60	240	0.544
EMA	2x4	0.6	0.9	66	264	0.6
EMA	2x4	0.6	0.9	72	288	0.659
EMA	2x4	0.6	0.9	80	320	0.676
EMA	2x4	0.6	0.95	6	24	0
EMA	2x4	0.6	0.95	10	40	0.005
EMA	2x4	0.6	0.95	14	56	0.019
EMA	2x4	0.6	0.95	18	72	0.103
EMA	2x4	0.6	0.95	22	88	0.209
EMA	2x4	0.6	0.95	26	104	0.331
EMA	2x4	0.6	0.95	30	120	0.383
EMA	2x4	0.6	0.95	32	128	0.434
EMA	2x4	0.6	0.95	36	144	0.528
EMA	2x4	0.6	0.95	40	160	0.573
EMA	2x4	0.6	0.95	46	184	0.663
EMA	2x4	0.6	0.95	50	200	0.711
EMA	2x4	0.6	0.95	54	216	0.74
EMA	2x4	0.6	0.95	60	240	0.793
EMA	2x4	0.6	0.95	66	264	0.822
EMA	2x4	0.6	0.95	72	288	0.841
EMA	2x4	0.6	0.95	80	320	0.896
EMA	2x4	0.6	1	6	24	0
EMA	2x4	0.6	1	10	40	0.003
EMA	2x4	0.6	1	14	56	0.026
EMA	2x4	0.6	1	18	72	0.1
EMA	2x4	0.6	1	22	88	0.253
EMA	2x4	0.6	1	24	96	0.284
EMA	2x4	0.6	1	26	104	0.337
EMA	2x4	0.6	1	30	120	0.436
EMA	2x4	0.6	1	36	144	0.558
EMA	2x4	0.6	1	40	160	0.637
EMA	2x4	0.6	1	46	184	0.746
EMA	2x4	0.6	1	50	200	0.772
EMA	2x4	0.6	1	54	216	0.798
EMA	2x4	0.6	1	60	240	0.878
EMA	2x4	0.6	1	66	264	0.884
EMA	2x4	0.6	1	72	288	0.934
EMA	2x4	0.6	1	80	320	0.945
EMA	2x4	0.6	1.05	6	24	0.001

EMA	2x4	0.6	1.05	10	40	0.005
EMA	2x4	0.6	1.05	14	56	0.03
EMA	2x4	0.6	1.05	18	72	0.094
EMA	2x4	0.6	1.05	22	88	0.228
EMA	2x4	0.6	1.05	24	96	0.246
EMA	2x4	0.6	1.05	26	104	0.316
EMA	2x4	0.6	1.05	32	128	0.475
EMA	2x4	0.6	1.05	36	144	0.533
EMA	2x4	0.6	1.05	40	160	0.597
EMA	2x4	0.6	1.05	46	184	0.68
EMA	2x4	0.6	1.05	50	200	0.717
EMA	2x4	0.6	1.05	54	216	0.776
EMA	2x4	0.6	1.05	60	240	0.797
EMA	2x4	0.6	1.05	66	264	0.835
EMA	2x4	0.6	1.05	72	288	0.853
EMA	2x4	0.6	1.05	80	320	0.887
EMA	2x4	0.6	1.1	6	24	0.001
EMA	2x4	0.6	1.1	12	48	0.012
EMA	2x4	0.6	1.1	18	72	0.089
EMA	2x4	0.6	1.1	24	96	0.223
EMA	2x4	0.6	1.1	30	120	0.341
EMA	2x4	0.6	1.1	32	128	0.378
EMA	2x4	0.6	1.1	36	144	0.434
EMA	2x4	0.6	1.1	42	168	0.514
EMA	2x4	0.6	1.1	46	184	0.514
EMA	2x4	0.6	1.1	50	200	0.577
EMA	2x4	0.6	1.1	54	216	0.61
EMA	2x4	0.6	1.1	60	240	0.63
EMA	2x4	0.6	1.1	66	264	0.663
EMA	2x4	0.6	1.1	72	288	0.725
EMA	2x4	0.6	1.1	80	320	0.757
FDA	2x2	0.2	0.9	12	24	0.035
FDA	2x2	0.2	0.9	16	32	0.109
FDA	2x2	0.2	0.9	24	48	0.254
FDA	2x2	0.2	0.9	28	56	0.335
FDA	2x2	0.2	0.9	32	64	0.358
FDA	2x2	0.2	0.9	36	72	0.442
FDA	2x2	0.2	0.9	38	76	0.473
FDA	2x2	0.2	0.9	50	100	0.597
FDA	2x2	0.2	0.9	60	120	0.669
FDA	2x2	0.2	0.9	70	140	0.734

FDA	2x2	0.2	0.9	80	160	0.801
FDA	2x2	0.2	0.9	90	180	0.856
FDA	2x2	0.2	0.9	100	200	0.872
FDA	2x2	0.2	0.9	108	216	0.91
FDA	2x2	0.2	0.9	120	240	0.928
FDA	2x2	0.2	0.9	132	264	0.931
FDA	2x2	0.2	0.9	144	288	0.962
FDA	2x2	0.2	0.9	160	320	0.975
FDA	2x2	0.2	0.95	12	24	0.051
FDA	2x2	0.2	0.95	16	32	0.14
FDA	2x2	0.2	0.95	20	40	0.28
FDA	2x2	0.2	0.95	26	52	0.465
FDA	2x2	0.2	0.95	32	64	0.638
FDA	2x2	0.2	0.95	36	72	0.686
FDA	2x2	0.2	0.95	40	80	0.755
FDA	2x2	0.2	0.95	50	100	0.836
FDA	2x2	0.2	0.95	60	120	0.907
FDA	2x2	0.2	0.95	70	140	0.949
FDA	2x2	0.2	0.95	80	160	0.972
FDA	2x2	0.2	0.95	90	180	0.987
FDA	2x2	0.2	0.95	100	200	0.991
FDA	2x2	0.2	0.95	108	216	0.995
FDA	2x2	0.2	0.95	120	240	0.994
FDA	2x2	0.2	0.95	132	264	0.999
FDA	2x2	0.2	0.95	144	288	0.998
FDA	2x2	0.2	0.95	160	320	1
FDA	2x2	0.2	1	12	24	0.056
FDA	2x2	0.2	1	16	32	0.165
FDA	2x2	0.2	1	20	40	0.303
FDA	2x2	0.2	1	28	56	0.578
FDA	2x2	0.2	1	36	72	0.743
FDA	2x2	0.2	1	40	80	0.801
FDA	2x2	0.2	1	50	100	0.917
FDA	2x2	0.2	1	60	120	0.96
FDA	2x2	0.2	1	70	140	0.977
FDA	2x2	0.2	1	80	160	0.99
FDA	2x2	0.2	1	90	180	1
FDA	2x2	0.2	1	100	200	1
FDA	2x2	0.2	1	108	216	0.999
FDA	2x2	0.2	1	120	240	1
FDA	2x2	0.2	1	132	264	1

FDA	2x2	0.2	1	144	288	1
FDA	2x2	0.2	1	160	320	1
FDA	2x2	0.2	1.05	12	24	0.058
FDA	2x2	0.2	1.05	18	36	0.201
FDA	2x2	0.2	1.05	24	48	0.387
FDA	2x2	0.2	1.05	28	56	0.487
FDA	2x2	0.2	1.05	32	64	0.585
FDA	2x2	0.2	1.05	36	72	0.632
FDA	2x2	0.2	1.05	40	80	0.708
FDA	2x2	0.2	1.05	50	100	0.812
FDA	2x2	0.2	1.05	60	120	0.883
FDA	2x2	0.2	1.05	70	140	0.918
FDA	2x2	0.2	1.05	80	160	0.937
FDA	2x2	0.2	1.05	90	180	0.96
FDA	2x2	0.2	1.05	100	200	0.979
FDA	2x2	0.2	1.05	108	216	0.981
FDA	2x2	0.2	1.05	120	240	0.994
FDA	2x2	0.2	1.05	132	264	0.992
FDA	2x2	0.2	1.05	144	288	0.999
FDA	2x2	0.2	1.05	160	320	1
FDA	2x2	0.2	1.1	12	24	0.039
FDA	2x2	0.2	1.1	16	32	0.119
FDA	2x2	0.2	1.1	20	40	0.202
FDA	2x2	0.2	1.1	28	56	0.346
FDA	2x2	0.2	1.1	32	64	0.393
FDA	2x2	0.2	1.1	36	72	0.489
FDA	2x2	0.2	1.1	40	80	0.523
FDA	2x2	0.2	1.1	44	88	0.559
FDA	2x2	0.2	1.1	60	120	0.671
FDA	2x2	0.2	1.1	70	140	0.729
FDA	2x2	0.2	1.1	80	160	0.775
FDA	2x2	0.2	1.1	90	180	0.789
FDA	2x2	0.2	1.1	100	200	0.851
FDA	2x2	0.2	1.1	108	216	0.863
FDA	2x2	0.2	1.1	120	240	0.922
FDA	2x2	0.2	1.1	132	264	0.937
FDA	2x2	0.2	1.1	144	288	0.94
FDA	2x2	0.2	1.1	160	320	0.964
FDA	2x2	0.3	0.9	12	24	0.01
FDA	2x2	0.3	0.9	24	48	0.145
FDA	2x2	0.3	0.9	36	72	0.329

FDA	2x2	0.3	0.9	48	96	0.479
FDA	2x2	0.3	0.9	60	120	0.573
FDA	2x2	0.3	0.9	72	144	0.613
FDA	2x2	0.3	0.9	80	160	0.664
FDA	2x2	0.3	0.9	88	176	0.71
FDA	2x2	0.3	0.9	100	200	0.753
FDA	2x2	0.3	0.9	108	216	0.78
FDA	2x2	0.3	0.9	120	240	0.819
FDA	2x2	0.3	0.9	132	264	0.861
FDA	2x2	0.3	0.9	144	288	0.889
FDA	2x2	0.3	0.9	160	320	0.932
FDA	2x2	0.3	0.95	12	24	0.023
FDA	2x2	0.3	0.95	16	32	0.047
FDA	2x2	0.3	0.95	24	48	0.204
FDA	2x2	0.3	0.95	28	56	0.28
FDA	2x2	0.3	0.95	32	64	0.388
FDA	2x2	0.3	0.95	36	72	0.489
FDA	2x2	0.3	0.95	40	80	0.568
FDA	2x2	0.3	0.95	52	104	0.703
FDA	2x2	0.3	0.95	60	120	0.811
FDA	2x2	0.3	0.95	72	144	0.852
FDA	2x2	0.3	0.95	80	160	0.913
FDA	2x2	0.3	0.95	88	176	0.929
FDA	2x2	0.3	0.95	100	200	0.955
FDA	2x2	0.3	0.95	108	216	0.968
FDA	2x2	0.3	0.95	120	240	0.978
FDA	2x2	0.3	0.95	132	264	0.984
FDA	2x2	0.3	0.95	144	288	0.995
FDA	2x2	0.3	0.95	160	320	0.993
FDA	2x2	0.3	1	12	24	0.018
FDA	2x2	0.3	1	16	32	0.044
FDA	2x2	0.3	1	24	48	0.228
FDA	2x2	0.3	1	28	56	0.338
FDA	2x2	0.3	1	32	64	0.445
FDA	2x2	0.3	1	36	72	0.53
FDA	2x2	0.3	1	40	80	0.61
FDA	2x2	0.3	1	44	88	0.662
FDA	2x2	0.3	1	60	120	0.834
FDA	2x2	0.3	1	72	144	0.921
FDA	2x2	0.3	1	80	160	0.948
FDA	2x2	0.3	1	88	176	0.978

FDA	2x2	0.3	1	100	200	0.987
FDA	2x2	0.3	1	108	216	0.992
FDA	2x2	0.3	1	120	240	0.995
FDA	2x2	0.3	1	132	264	0.999
FDA	2x2	0.3	1	144	288	0.999
FDA	2x2	0.3	1	160	320	0.999
FDA	2x2	0.3	1.05	12	24	0.018
FDA	2x2	0.3	1.05	16	32	0.036
FDA	2x2	0.3	1.05	24	48	0.188
FDA	2x2	0.3	1.05	28	56	0.299
FDA	2x2	0.3	1.05	32	64	0.367
FDA	2x2	0.3	1.05	38	76	0.496
FDA	2x2	0.3	1.05	40	80	0.517
FDA	2x2	0.3	1.05	48	96	0.636
FDA	2x2	0.3	1.05	52	104	0.684
FDA	2x2	0.3	1.05	60	120	0.719
FDA	2x2	0.3	1.05	72	144	0.845
FDA	2x2	0.3	1.05	80	160	0.86
FDA	2x2	0.3	1.05	88	176	0.885
FDA	2x2	0.3	1.05	100	200	0.936
FDA	2x2	0.3	1.05	108	216	0.952
FDA	2x2	0.3	1.05	120	240	0.974
FDA	2x2	0.3	1.05	132	264	0.978
FDA	2x2	0.3	1.05	144	288	0.978
FDA	2x2	0.3	1.05	160	320	0.988
FDA	2x2	0.3	1.1	12	24	0.012
FDA	2x2	0.3	1.1	24	48	0.138
FDA	2x2	0.3	1.1	36	72	0.329
FDA	2x2	0.3	1.1	48	96	0.458
FDA	2x2	0.3	1.1	60	120	0.519
FDA	2x2	0.3	1.1	68	136	0.569
FDA	2x2	0.3	1.1	76	152	0.658
FDA	2x2	0.3	1.1	84	168	0.699
FDA	2x2	0.3	1.1	92	184	0.679
FDA	2x2	0.3	1.1	100	200	0.74
FDA	2x2	0.3	1.1	72	144	0.594
FDA	2x2	0.3	1.1	80	160	0.645
FDA	2x2	0.3	1.1	88	176	0.701
FDA	2x2	0.3	1.1	100	200	0.743
FDA	2x2	0.3	1.1	108	216	0.761
FDA	2x2	0.3	1.1	120	240	0.805

FDA	2x2	0.3	1.1	132	264	0.83
FDA	2x2	0.3	1.1	144	288	0.893
FDA	2x2	0.3	1.1	160	320	0.904
FDA	2x2	0.4	0.9	12	24	0.004
FDA	2x2	0.4	0.9	24	48	0.058
FDA	2x2	0.4	0.9	36	72	0.183
FDA	2x2	0.4	0.9	48	96	0.334
FDA	2x2	0.4	0.9	56	112	0.369
FDA	2x2	0.4	0.9	64	128	0.473
FDA	2x2	0.4	0.9	68	136	0.469
FDA	2x2	0.4	0.9	76	152	0.54
FDA	2x2	0.4	0.9	84	168	0.572
FDA	2x2	0.4	0.9	96	192	0.643
FDA	2x2	0.4	0.9	108	216	0.671
FDA	2x2	0.4	0.9	120	240	0.716
FDA	2x2	0.4	0.9	132	264	0.776
FDA	2x2	0.4	0.9	144	288	0.777
FDA	2x2	0.4	0.9	160	320	0.813
FDA	2x2	0.4	0.95	12	24	0.009
FDA	2x2	0.4	0.95	24	48	0.077
FDA	2x2	0.4	0.95	36	72	0.266
FDA	2x2	0.4	0.95	40	80	0.338
FDA	2x2	0.4	0.95	44	88	0.42
FDA	2x2	0.4	0.95	52	104	0.535
FDA	2x2	0.4	0.95	56	112	0.584
FDA	2x2	0.4	0.95	64	128	0.665
FDA	2x2	0.4	0.95	68	136	0.663
FDA	2x2	0.4	0.95	76	152	0.749
FDA	2x2	0.4	0.95	84	168	0.818
FDA	2x2	0.4	0.95	96	192	0.859
FDA	2x2	0.4	0.95	108	216	0.908
FDA	2x2	0.4	0.95	120	240	0.932
FDA	2x2	0.4	0.95	132	264	0.95
FDA	2x2	0.4	0.95	144	288	0.97
FDA	2x2	0.4	0.95	160	320	0.981
FDA	2x2	0.4	1	12	24	0.007
FDA	2x2	0.4	1	24	48	0.07
FDA	2x2	0.4	1	36	72	0.29
FDA	2x2	0.4	1	40	80	0.385
FDA	2x2	0.4	1	48	96	0.521
FDA	2x2	0.4	1	56	112	0.65

FDA	2x2	0.4	1	64	128	0.737
FDA	2x2	0.4	1	68	136	0.787
FDA	2x2	0.4	1	76	152	0.809
FDA	2x2	0.4	1	84	168	0.866
FDA	2x2	0.4	1	96	192	0.924
FDA	2x2	0.4	1	108	216	0.944
FDA	2x2	0.4	1	120	240	0.971
FDA	2x2	0.4	1	132	264	0.978
FDA	2x2	0.4	1	144	288	0.985
FDA	2x2	0.4	1	160	320	0.994
FDA	2x2	0.4	1.05	12	24	0.005
FDA	2x2	0.4	1.05	24	48	0.053
FDA	2x2	0.4	1.05	36	72	0.268
FDA	2x2	0.4	1.05	40	80	0.336
FDA	2x2	0.4	1.05	44	88	0.397
FDA	2x2	0.4	1.05	52	104	0.51
FDA	2x2	0.4	1.05	60	120	0.574
FDA	2x2	0.4	1.05	64	128	0.661
FDA	2x2	0.4	1.05	68	136	0.66
FDA	2x2	0.4	1.05	76	152	0.735
FDA	2x2	0.4	1.05	84	168	0.774
FDA	2x2	0.4	1.05	96	192	0.84
FDA	2x2	0.4	1.05	108	216	0.882
FDA	2x2	0.4	1.05	120	240	0.891
FDA	2x2	0.4	1.05	132	264	0.934
FDA	2x2	0.4	1.05	144	288	0.945
FDA	2x2	0.4	1.05	160	320	0.962
FDA	2x2	0.4	1.1	12	24	0.005
FDA	2x2	0.4	1.1	24	48	0.056
FDA	2x2	0.4	1.1	36	72	0.197
FDA	2x2	0.4	1.1	48	96	0.336
FDA	2x2	0.4	1.1	56	112	0.389
FDA	2x2	0.4	1.1	64	128	0.457
FDA	2x2	0.4	1.1	72	144	0.519
FDA	2x2	0.4	1.1	80	160	0.531
FDA	2x2	0.4	1.1	88	176	0.593
FDA	2x2	0.4	1.1	96	192	0.606
FDA	2x2	0.4	1.1	108	216	0.683
FDA	2x2	0.4	1.1	120	240	0.713
FDA	2x2	0.4	1.1	132	264	0.756
FDA	2x2	0.4	1.1	144	288	0.782

FDA	2x2	0.4	1.1	160	320	0.804
FDA	2x2	0.5	0.9	12	24	0
FDA	2x2	0.5	0.9	24	48	0.012
FDA	2x2	0.5	0.9	36	72	0.08
FDA	2x2	0.5	0.9	48	96	0.204
FDA	2x2	0.5	0.9	56	112	0.284
FDA	2x2	0.5	0.9	64	128	0.364
FDA	2x2	0.5	0.9	72	144	0.403
FDA	2x2	0.5	0.9	80	160	0.437
FDA	2x2	0.5	0.9	88	176	0.472
FDA	2x2	0.5	0.9	96	192	0.535
FDA	2x2	0.5	0.9	108	216	0.581
FDA	2x2	0.5	0.9	120	240	0.619
FDA	2x2	0.5	0.9	132	264	0.679
FDA	2x2	0.5	0.9	144	288	0.694
FDA	2x2	0.5	0.9	160	320	0.752
FDA	2x2	0.5	0.95	12	24	0
FDA	2x2	0.5	0.95	24	48	0.013
FDA	2x2	0.5	0.95	36	72	0.13
FDA	2x2	0.5	0.95	44	88	0.252
FDA	2x2	0.5	0.95	52	104	0.364
FDA	2x2	0.5	0.95	56	112	0.408
FDA	2x2	0.5	0.95	64	128	0.49
FDA	2x2	0.5	0.95	72	144	0.581
FDA	2x2	0.5	0.95	80	160	0.65
FDA	2x2	0.5	0.95	88	176	0.737
FDA	2x2	0.5	0.95	96	192	0.772
FDA	2x2	0.5	0.95	108	216	0.821
FDA	2x2	0.5	0.95	120	240	0.862
FDA	2x2	0.5	0.95	132	264	0.889
FDA	2x2	0.5	0.95	144	288	0.924
FDA	2x2	0.5	0.95	160	320	0.946
FDA	2x2	0.5	1	12	24	0.004
FDA	2x2	0.5	1	24	48	0.022
FDA	2x2	0.5	1	36	72	0.11
FDA	2x2	0.5	1	40	80	0.174
FDA	2x2	0.5	1	48	96	0.298
FDA	2x2	0.5	1	52	104	0.378
FDA	2x2	0.5	1	56	112	0.41
FDA	2x2	0.5	1	64	128	0.566
FDA	2x2	0.5	1	72	144	0.654

FDA	2x2	0.5	1	80	160	0.712
FDA	2x2	0.5	1	88	176	0.752
FDA	2x2	0.5	1	96	192	0.8
FDA	2x2	0.5	1	108	216	0.862
FDA	2x2	0.5	1	120	240	0.911
FDA	2x2	0.5	1	132	264	0.937
FDA	2x2	0.5	1	144	288	0.954
FDA	2x2	0.5	1	160	320	0.976
FDA	2x2	0.5	1.05	12	24	0.002
FDA	2x2	0.5	1.05	24	48	0.011
FDA	2x2	0.5	1.05	36	72	0.121
FDA	2x2	0.5	1.05	44	88	0.241
FDA	2x2	0.5	1.05	48	96	0.291
FDA	2x2	0.5	1.05	56	112	0.387
FDA	2x2	0.5	1.05	64	128	0.463
FDA	2x2	0.5	1.05	72	144	0.56
FDA	2x2	0.5	1.05	80	160	0.631
FDA	2x2	0.5	1.05	88	176	0.684
FDA	2x2	0.5	1.05	96	192	0.72
FDA	2x2	0.5	1.05	108	216	0.759
FDA	2x2	0.5	1.05	120	240	0.835
FDA	2x2	0.5	1.05	132	264	0.859
FDA	2x2	0.5	1.05	144	288	0.865
FDA	2x2	0.5	1.05	160	320	0.911
FDA	2x2	0.5	1.1	12	24	0.002
FDA	2x2	0.5	1.1	24	48	0.008
FDA	2x2	0.5	1.1	36	72	0.11
FDA	2x2	0.5	1.1	44	88	0.202
FDA	2x2	0.5	1.1	56	112	0.298
FDA	2x2	0.5	1.1	68	136	0.376
FDA	2x2	0.5	1.1	72	144	0.414
FDA	2x2	0.5	1.1	76	152	0.437
FDA	2x2	0.5	1.1	80	160	0.45
FDA	2x2	0.5	1.1	88	176	0.505
FDA	2x2	0.5	1.1	96	192	0.525
FDA	2x2	0.5	1.1	108	216	0.57
FDA	2x2	0.5	1.1	120	240	0.598
FDA	2x2	0.5	1.1	132	264	0.653
FDA	2x2	0.5	1.1	144	288	0.718
FDA	2x2	0.5	1.1	160	320	0.739
FDA	2x2	0.6	0.9	12	24	0

FDA	2x2	0.6	0.9	20	40	0.001
FDA	2x2	0.6	0.9	28	56	0.004
FDA	2x2	0.6	0.9	36	72	0.034
FDA	2x2	0.6	0.9	44	88	0.076
FDA	2x2	0.6	0.9	52	104	0.126
FDA	2x2	0.6	0.9	64	128	0.275
FDA	2x2	0.6	0.9	72	144	0.306
FDA	2x2	0.6	0.9	80	160	0.37
FDA	2x2	0.6	0.9	92	184	0.429
FDA	2x2	0.6	0.9	100	200	0.443
FDA	2x2	0.6	0.9	108	216	0.489
FDA	2x2	0.6	0.9	120	240	0.528
FDA	2x2	0.6	0.9	132	264	0.611
FDA	2x2	0.6	0.9	144	288	0.597
FDA	2x2	0.6	0.9	160	320	0.652
FDA	2x2	0.6	0.95	12	24	0
FDA	2x2	0.6	0.95	20	40	0.004
FDA	2x2	0.6	0.95	28	56	0.012
FDA	2x2	0.6	0.95	36	72	0.045
FDA	2x2	0.6	0.95	44	88	0.094
FDA	2x2	0.6	0.95	52	104	0.197
FDA	2x2	0.6	0.95	60	120	0.283
FDA	2x2	0.6	0.95	64	128	0.322
FDA	2x2	0.6	0.95	72	144	0.439
FDA	2x2	0.6	0.95	80	160	0.495
FDA	2x2	0.6	0.95	92	184	0.578
FDA	2x2	0.6	0.95	100	200	0.64
FDA	2x2	0.6	0.95	108	216	0.695
FDA	2x2	0.6	0.95	120	240	0.774
FDA	2x2	0.6	0.95	132	264	0.806
FDA	2x2	0.6	0.95	144	288	0.824
FDA	2x2	0.6	0.95	160	320	0.845
FDA	2x2	0.6	1	12	24	0
FDA	2x2	0.6	1	20	40	0.004
FDA	2x2	0.6	1	28	56	0.009
FDA	2x2	0.6	1	36	72	0.034
FDA	2x2	0.6	1	44	88	0.114
FDA	2x2	0.6	1	48	96	0.165
FDA	2x2	0.6	1	52	104	0.214
FDA	2x2	0.6	1	60	120	0.329
FDA	2x2	0.6	1	72	144	0.482

FDA	2x2	0.6	1	80	160	0.559
FDA	2x2	0.6	1	92	184	0.658
FDA	2x2	0.6	1	100	200	0.714
FDA	2x2	0.6	1	108	216	0.763
FDA	2x2	0.6	1	120	240	0.815
FDA	2x2	0.6	1	132	264	0.864
FDA	2x2	0.6	1	144	288	0.889
FDA	2x2	0.6	1	160	320	0.93
FDA	2x2	0.6	1.05	12	24	0
FDA	2x2	0.6	1.05	20	40	0.004
FDA	2x2	0.6	1.05	28	56	0.015
FDA	2x2	0.6	1.05	36	72	0.038
FDA	2x2	0.6	1.05	44	88	0.105
FDA	2x2	0.6	1.05	48	96	0.14
FDA	2x2	0.6	1.05	52	104	0.207
FDA	2x2	0.6	1.05	64	128	0.334
FDA	2x2	0.6	1.05	72	144	0.445
FDA	2x2	0.6	1.05	80	160	0.501
FDA	2x2	0.6	1.05	92	184	0.579
FDA	2x2	0.6	1.05	100	200	0.633
FDA	2x2	0.6	1.05	108	216	0.682
FDA	2x2	0.6	1.05	120	240	0.743
FDA	2x2	0.6	1.05	132	264	0.761
FDA	2x2	0.6	1.05	144	288	0.805
FDA	2x2	0.6	1.05	160	320	0.847
FDA	2x2	0.6	1.1	12	24	0
FDA	2x2	0.6	1.1	24	48	0.002
FDA	2x2	0.6	1.1	36	72	0.037
FDA	2x2	0.6	1.1	48	96	0.119
FDA	2x2	0.6	1.1	60	120	0.233
FDA	2x2	0.6	1.1	64	128	0.277
FDA	2x2	0.6	1.1	72	144	0.325
FDA	2x2	0.6	1.1	84	168	0.407
FDA	2x2	0.6	1.1	92	184	0.436
FDA	2x2	0.6	1.1	100	200	0.451
FDA	2x2	0.6	1.1	108	216	0.494
FDA	2x2	0.6	1.1	120	240	0.5
FDA	2x2	0.6	1.1	132	264	0.58
FDA	2x2	0.6	1.1	144	288	0.614
FDA	2x2	0.6	1.1	160	320	0.637
FDA	3x3	0.2	0.9	9	27	0.079

FDA	3x3	0.2	0.9	12	36	0.171
FDA	3x3	0.2	0.9	15	45	0.281
FDA	3x3	0.2	0.9	18	54	0.367
FDA	3x3	0.2	0.9	21	63	0.43
FDA	3x3	0.2	0.9	24	72	0.49
FDA	3x3	0.2	0.9	27	81	0.5
FDA	3x3	0.2	0.9	33	99	0.632
FDA	3x3	0.2	0.9	39	117	0.645
FDA	3x3	0.2	0.9	45	135	0.72
FDA	3x3	0.2	0.9	54	162	0.811
FDA	3x3	0.2	0.9	60	180	0.811
FDA	3x3	0.2	0.9	66	198	0.848
FDA	3x3	0.2	0.9	75	225	0.897
FDA	3x3	0.2	0.9	81	243	0.896
FDA	3x3	0.2	0.9	90	270	0.932
FDA	3x3	0.2	0.9	96	288	0.943
FDA	3x3	0.2	0.9	105	315	0.963
FDA	3x3	0.2	0.95	9	27	0.098
FDA	3x3	0.2	0.95	12	36	0.233
FDA	3x3	0.2	0.95	15	45	0.367
FDA	3x3	0.2	0.95	18	54	0.476
FDA	3x3	0.2	0.95	21	63	0.593
FDA	3x3	0.2	0.95	24	72	0.668
FDA	3x3	0.2	0.95	27	81	0.707
FDA	3x3	0.2	0.95	33	99	0.824
FDA	3x3	0.2	0.95	39	117	0.893
FDA	3x3	0.2	0.95	45	135	0.921
FDA	3x3	0.2	0.95	54	162	0.962
FDA	3x3	0.2	0.95	60	180	0.97
FDA	3x3	0.2	0.95	66	198	0.986
FDA	3x3	0.2	0.95	75	225	0.984
FDA	3x3	0.2	0.95	81	243	0.995
FDA	3x3	0.2	0.95	90	270	0.996
FDA	3x3	0.2	0.95	96	288	0.997
FDA	3x3	0.2	0.95	105	315	0.997
FDA	3x3	0.2	1	9	27	0.112
FDA	3x3	0.2	1	12	36	0.265
FDA	3x3	0.2	1	15	45	0.417
FDA	3x3	0.2	1	18	54	0.53
FDA	3x3	0.2	1	24	72	0.716
FDA	3x3	0.2	1	27	81	0.811

FDA	3x3	0.2	1	33	99	0.875
FDA	3x3	0.2	1	39	117	0.92
FDA	3x3	0.2	1	45	135	0.961
FDA	3x3	0.2	1	54	162	0.979
FDA	3x3	0.2	1	60	180	0.988
FDA	3x3	0.2	1	66	198	0.992
FDA	3x3	0.2	1	75	225	0.997
FDA	3x3	0.2	1	81	243	0.997
FDA	3x3	0.2	1	90	270	1
FDA	3x3	0.2	1	96	288	0.999
FDA	3x3	0.2	1	105	315	1
FDA	3x3	0.2	1.05	9	27	0.106
FDA	3x3	0.2	1.05	12	36	0.235
FDA	3x3	0.2	1.05	15	45	0.359
FDA	3x3	0.2	1.05	18	54	0.467
FDA	3x3	0.2	1.05	21	63	0.558
FDA	3x3	0.2	1.05	24	72	0.624
FDA	3x3	0.2	1.05	27	81	0.681
FDA	3x3	0.2	1.05	33	99	0.767
FDA	3x3	0.2	1.05	39	117	0.864
FDA	3x3	0.2	1.05	45	135	0.893
FDA	3x3	0.2	1.05	54	162	0.931
FDA	3x3	0.2	1.05	60	180	0.946
FDA	3x3	0.2	1.05	66	198	0.971
FDA	3x3	0.2	1.05	75	225	0.971
FDA	3x3	0.2	1.05	81	243	0.985
FDA	3x3	0.2	1.05	90	270	0.991
FDA	3x3	0.2	1.05	96	288	0.995
FDA	3x3	0.2	1.05	105	315	0.996
FDA	3x3	0.2	1.1	9	27	0.086
FDA	3x3	0.2	1.1	12	36	0.173
FDA	3x3	0.2	1.1	15	45	0.292
FDA	3x3	0.2	1.1	18	54	0.368
FDA	3x3	0.2	1.1	21	63	0.428
FDA	3x3	0.2	1.1	24	72	0.449
FDA	3x3	0.2	1.1	27	81	0.504
FDA	3x3	0.2	1.1	30	90	0.56
FDA	3x3	0.2	1.1	39	117	0.65
FDA	3x3	0.2	1.1	45	135	0.709
FDA	3x3	0.2	1.1	54	162	0.76
FDA	3x3	0.2	1.1	60	180	0.804

FDA	3x3	0.2	1.1	66	198	0.826
FDA	3x3	0.2	1.1	75	225	0.872
FDA	3x3	0.2	1.1	81	243	0.887
FDA	3x3	0.2	1.1	90	270	0.92
FDA	3x3	0.2	1.1	96	288	0.942
FDA	3x3	0.2	1.1	105	315	0.953
FDA	3x3	0.3	0.9	9	27	0.044
FDA	3x3	0.3	0.9	15	45	0.202
FDA	3x3	0.3	0.9	24	72	0.407
FDA	3x3	0.3	0.9	33	99	0.541
FDA	3x3	0.3	0.9	39	117	0.603
FDA	3x3	0.3	0.9	48	144	0.708
FDA	3x3	0.3	0.9	54	162	0.744
FDA	3x3	0.3	0.9	60	180	0.782
FDA	3x3	0.3	0.9	66	198	0.795
FDA	3x3	0.3	0.9	75	225	0.82
FDA	3x3	0.3	0.9	81	243	0.85
FDA	3x3	0.3	0.9	90	270	0.881
FDA	3x3	0.3	0.9	96	288	0.873
FDA	3x3	0.3	0.9	105	315	0.923
FDA	3x3	0.3	0.95	9	27	0.058
FDA	3x3	0.3	0.95	12	36	0.127
FDA	3x3	0.3	0.95	15	45	0.241
FDA	3x3	0.3	0.95	18	54	0.351
FDA	3x3	0.3	0.95	21	63	0.433
FDA	3x3	0.3	0.95	24	72	0.547
FDA	3x3	0.3	0.95	27	81	0.61
FDA	3x3	0.3	0.95	36	108	0.781
FDA	3x3	0.3	0.95	39	117	0.804
FDA	3x3	0.3	0.95	48	144	0.882
FDA	3x3	0.3	0.95	54	162	0.906
FDA	3x3	0.3	0.95	60	180	0.925
FDA	3x3	0.3	0.95	66	198	0.955
FDA	3x3	0.3	0.95	75	225	0.972
FDA	3x3	0.3	0.95	81	243	0.974
FDA	3x3	0.3	0.95	90	270	0.985
FDA	3x3	0.3	0.95	96	288	0.989
FDA	3x3	0.3	0.95	105	315	0.997
FDA	3x3	0.3	1	9	27	0.07
FDA	3x3	0.3	1	12	36	0.145
FDA	3x3	0.3	1	15	45	0.276

FDA	3x3	0.3	1	18	54	0.389
FDA	3x3	0.3	1	21	63	0.494
FDA	3x3	0.3	1	24	72	0.579
FDA	3x3	0.3	1	27	81	0.677
FDA	3x3	0.3	1	30	90	0.749
FDA	3x3	0.3	1	39	117	0.832
FDA	3x3	0.3	1	48	144	0.937
FDA	3x3	0.3	1	54	162	0.943
FDA	3x3	0.3	1	60	180	0.977
FDA	3x3	0.3	1	66	198	0.985
FDA	3x3	0.3	1	75	225	0.992
FDA	3x3	0.3	1	81	243	0.995
FDA	3x3	0.3	1	90	270	0.995
FDA	3x3	0.3	1	96	288	0.999
FDA	3x3	0.3	1	105	315	0.998
FDA	3x3	0.3	1.05	9	27	0.071
FDA	3x3	0.3	1.05	12	36	0.122
FDA	3x3	0.3	1.05	15	45	0.239
FDA	3x3	0.3	1.05	18	54	0.353
FDA	3x3	0.3	1.05	21	63	0.436
FDA	3x3	0.3	1.05	24	72	0.537
FDA	3x3	0.3	1.05	27	81	0.6
FDA	3x3	0.3	1.05	33	99	0.728
FDA	3x3	0.3	1.05	36	108	0.741
FDA	3x3	0.3	1.05	39	117	0.76
FDA	3x3	0.3	1.05	48	144	0.848
FDA	3x3	0.3	1.05	54	162	0.91
FDA	3x3	0.3	1.05	60	180	0.914
FDA	3x3	0.3	1.05	66	198	0.941
FDA	3x3	0.3	1.05	75	225	0.96
FDA	3x3	0.3	1.05	81	243	0.966
FDA	3x3	0.3	1.05	90	270	0.983
FDA	3x3	0.3	1.05	96	288	0.982
FDA	3x3	0.3	1.05	105	315	0.992
FDA	3x3	0.3	1.1	9	27	0.048
FDA	3x3	0.3	1.1	15	45	0.197
FDA	3x3	0.3	1.1	24	72	0.436
FDA	3x3	0.3	1.1	33	99	0.574
FDA	3x3	0.3	1.1	39	117	0.618
FDA	3x3	0.3	1.1	45	135	0.684
FDA	3x3	0.3	1.1	51	153	0.725

FDA	3x3	0.3	1.1	51	153	0.686
FDA	3x3	0.3	1.1	63	189	0.794
FDA	3x3	0.3	1.1	66	198	0.789
FDA	3x3	0.3	1.1	48	144	0.682
FDA	3x3	0.3	1.1	54	162	0.728
FDA	3x3	0.3	1.1	60	180	0.778
FDA	3x3	0.3	1.1	66	198	0.803
FDA	3x3	0.3	1.1	75	225	0.825
FDA	3x3	0.3	1.1	81	243	0.871
FDA	3x3	0.3	1.1	90	270	0.884
FDA	3x3	0.3	1.1	96	288	0.906
FDA	3x3	0.3	1.1	105	315	0.921
FDA	3x3	0.4	0.9	9	27	0.069
FDA	3x3	0.4	0.9	15	45	0.235
FDA	3x3	0.4	0.9	24	72	0.475
FDA	3x3	0.4	0.9	33	99	0.703
FDA	3x3	0.4	0.9	39	117	0.745
FDA	3x3	0.4	0.9	42	126	0.759
FDA	3x3	0.4	0.9	45	135	0.808
FDA	3x3	0.4	0.9	51	153	0.822
FDA	3x3	0.4	0.9	54	162	0.895
FDA	3x3	0.4	0.9	63	189	0.914
FDA	3x3	0.4	0.9	72	216	0.939
FDA	3x3	0.4	0.9	81	243	0.948
FDA	3x3	0.4	0.9	90	270	0.971
FDA	3x3	0.4	0.9	96	288	0.981
FDA	3x3	0.4	0.9	105	315	0.983
FDA	3x3	0.4	0.95	9	27	0.066
FDA	3x3	0.4	0.95	15	45	0.254
FDA	3x3	0.4	0.95	24	72	0.558
FDA	3x3	0.4	0.95	27	81	0.674
FDA	3x3	0.4	0.95	30	90	0.711
FDA	3x3	0.4	0.95	36	108	0.84
FDA	3x3	0.4	0.95	39	117	0.842
FDA	3x3	0.4	0.95	42	126	0.876
FDA	3x3	0.4	0.95	45	135	0.919
FDA	3x3	0.4	0.95	51	153	0.942
FDA	3x3	0.4	0.95	54	162	0.959
FDA	3x3	0.4	0.95	63	189	0.979
FDA	3x3	0.4	0.95	72	216	0.982
FDA	3x3	0.4	0.95	81	243	0.993

FDA	3x3	0.4	0.95	90	270	0.996
FDA	3x3	0.4	0.95	96	288	0.999
FDA	3x3	0.4	0.95	105	315	1
FDA	3x3	0.4	1	9	27	0.094
FDA	3x3	0.4	1	15	45	0.265
FDA	3x3	0.4	1	24	72	0.605
FDA	3x3	0.4	1	27	81	0.708
FDA	3x3	0.4	1	33	99	0.816
FDA	3x3	0.4	1	39	117	0.902
FDA	3x3	0.4	1	42	126	0.92
FDA	3x3	0.4	1	45	135	0.949
FDA	3x3	0.4	1	51	153	0.951
FDA	3x3	0.4	1	54	162	0.97
FDA	3x3	0.4	1	63	189	0.996
FDA	3x3	0.4	1	72	216	0.996
FDA	3x3	0.4	1	81	243	0.999
FDA	3x3	0.4	1	90	270	1
FDA	3x3	0.4	1	96	288	1
FDA	3x3	0.4	1	105	315	1
FDA	3x3	0.4	1.05	9	27	0.085
FDA	3x3	0.4	1.05	15	45	0.267
FDA	3x3	0.4	1.05	24	72	0.594
FDA	3x3	0.4	1.05	27	81	0.665
FDA	3x3	0.4	1.05	30	90	0.714
FDA	3x3	0.4	1.05	36	108	0.81
FDA	3x3	0.4	1.05	39	117	0.86
FDA	3x3	0.4	1.05	42	126	0.87
FDA	3x3	0.4	1.05	45	135	0.896
FDA	3x3	0.4	1.05	51	153	0.934
FDA	3x3	0.4	1.05	54	162	0.94
FDA	3x3	0.4	1.05	63	189	0.968
FDA	3x3	0.4	1.05	72	216	0.982
FDA	3x3	0.4	1.05	81	243	0.993
FDA	3x3	0.4	1.05	90	270	0.998
FDA	3x3	0.4	1.05	96	288	0.995
FDA	3x3	0.4	1.05	105	315	0.999
FDA	3x3	0.4	1.1	9	27	0.075
FDA	3x3	0.4	1.1	15	45	0.227
FDA	3x3	0.4	1.1	24	72	0.506
FDA	3x3	0.4	1.1	33	99	0.666
FDA	3x3	0.4	1.1	39	117	0.75

FDA	3x3	0.4	1.1	42	126	0.788
FDA	3x3	0.4	1.1	48	144	0.837
FDA	3x3	0.4	1.1	54	162	0.867
FDA	3x3	0.4	1.1	57	171	0.893
FDA	3x3	0.4	1.1	63	189	0.899
FDA	3x3	0.4	1.1	72	216	0.951
FDA	3x3	0.4	1.1	81	243	0.966
FDA	3x3	0.4	1.1	90	270	0.97
FDA	3x3	0.4	1.1	96	288	0.974
FDA	3x3	0.4	1.1	105	315	0.983
FDA	3x3	0.5	0.9	9	27	0.104
FDA	3x3	0.5	0.9	15	45	0.311
FDA	3x3	0.5	0.9	24	72	0.576
FDA	3x3	0.5	0.9	33	99	0.738
FDA	3x3	0.5	0.9	39	117	0.819
FDA	3x3	0.5	0.9	42	126	0.811
FDA	3x3	0.5	0.9	48	144	0.863
FDA	3x3	0.5	0.9	54	162	0.888
FDA	3x3	0.5	0.9	57	171	0.907
FDA	3x3	0.5	0.9	63	189	0.949
FDA	3x3	0.5	0.9	72	216	0.957
FDA	3x3	0.5	0.9	81	243	0.968
FDA	3x3	0.5	0.9	90	270	0.971
FDA	3x3	0.5	0.9	96	288	0.976
FDA	3x3	0.5	0.9	105	315	0.981
FDA	3x3	0.5	0.95	9	27	0.11
FDA	3x3	0.5	0.95	15	45	0.302
FDA	3x3	0.5	0.95	24	72	0.635
FDA	3x3	0.5	0.95	30	90	0.783
FDA	3x3	0.5	0.95	36	108	0.867
FDA	3x3	0.5	0.95	39	117	0.891
FDA	3x3	0.5	0.95	42	126	0.909
FDA	3x3	0.5	0.95	48	144	0.937
FDA	3x3	0.5	0.95	54	162	0.971
FDA	3x3	0.5	0.95	57	171	0.966
FDA	3x3	0.5	0.95	63	189	0.982
FDA	3x3	0.5	0.95	72	216	0.989
FDA	3x3	0.5	0.95	81	243	0.993
FDA	3x3	0.5	0.95	90	270	0.997
FDA	3x3	0.5	0.95	96	288	0.994
FDA	3x3	0.5	0.95	105	315	0.999

FDA	3x3	0.5	1	9	27	0.117
FDA	3x3	0.5	1	15	45	0.338
FDA	3x3	0.5	1	24	72	0.67
FDA	3x3	0.5	1	27	81	0.74
FDA	3x3	0.5	1	33	99	0.876
FDA	3x3	0.5	1	36	108	0.904
FDA	3x3	0.5	1	39	117	0.906
FDA	3x3	0.5	1	42	126	0.945
FDA	3x3	0.5	1	48	144	0.97
FDA	3x3	0.5	1	54	162	0.986
FDA	3x3	0.5	1	57	171	0.988
FDA	3x3	0.5	1	63	189	0.989
FDA	3x3	0.5	1	72	216	0.997
FDA	3x3	0.5	1	81	243	0.997
FDA	3x3	0.5	1	90	270	0.999
FDA	3x3	0.5	1	96	288	0.998
FDA	3x3	0.5	1	105	315	1
FDA	3x3	0.5	1.05	9	27	0.091
FDA	3x3	0.5	1.05	15	45	0.289
FDA	3x3	0.5	1.05	24	72	0.652
FDA	3x3	0.5	1.05	30	90	0.781
FDA	3x3	0.5	1.05	33	99	0.82
FDA	3x3	0.5	1.05	39	117	0.884
FDA	3x3	0.5	1.05	42	126	0.915
FDA	3x3	0.5	1.05	48	144	0.949
FDA	3x3	0.5	1.05	54	162	0.958
FDA	3x3	0.5	1.05	57	171	0.971
FDA	3x3	0.5	1.05	63	189	0.98
FDA	3x3	0.5	1.05	72	216	0.988
FDA	3x3	0.5	1.05	81	243	0.993
FDA	3x3	0.5	1.05	90	270	0.989
FDA	3x3	0.5	1.05	96	288	0.995
FDA	3x3	0.5	1.05	105	315	0.995
FDA	3x3	0.5	1.1	9	27	0.091
FDA	3x3	0.5	1.1	15	45	0.291
FDA	3x3	0.5	1.1	24	72	0.596
FDA	3x3	0.5	1.1	30	90	0.687
FDA	3x3	0.5	1.1	39	117	0.824
FDA	3x3	0.5	1.1	45	135	0.861
FDA	3x3	0.5	1.1	48	144	0.876
FDA	3x3	0.5	1.1	51	153	0.886

FDA	3x3	0.5	1.1	54	162	0.898
FDA	3x3	0.5	1.1	57	171	0.907
FDA	3x3	0.5	1.1	63	189	0.938
FDA	3x3	0.5	1.1	72	216	0.954
FDA	3x3	0.5	1.1	81	243	0.958
FDA	3x3	0.5	1.1	90	270	0.972
FDA	3x3	0.5	1.1	96	288	0.979
FDA	3x3	0.5	1.1	105	315	0.976
FDA	3x3	0.6	0.9	9	27	0.108
FDA	3x3	0.6	0.9	15	45	0.318
FDA	3x3	0.6	0.9	18	54	0.437
FDA	3x3	0.6	0.9	24	72	0.599
FDA	3x3	0.6	0.9	30	90	0.704
FDA	3x3	0.6	0.9	36	108	0.804
FDA	3x3	0.6	0.9	42	126	0.849
FDA	3x3	0.6	0.9	48	144	0.867
FDA	3x3	0.6	0.9	54	162	0.9
FDA	3x3	0.6	0.9	60	180	0.897
FDA	3x3	0.6	0.9	66	198	0.929
FDA	3x3	0.6	0.9	72	216	0.935
FDA	3x3	0.6	0.9	81	243	0.955
FDA	3x3	0.6	0.9	90	270	0.944
FDA	3x3	0.6	0.9	96	288	0.967
FDA	3x3	0.6	0.9	105	315	0.971
FDA	3x3	0.6	0.95	9	27	0.121
FDA	3x3	0.6	0.95	15	45	0.335
FDA	3x3	0.6	0.95	18	54	0.457
FDA	3x3	0.6	0.95	24	72	0.71
FDA	3x3	0.6	0.95	30	90	0.816
FDA	3x3	0.6	0.95	36	108	0.862
FDA	3x3	0.6	0.95	39	117	0.897
FDA	3x3	0.6	0.95	45	135	0.931
FDA	3x3	0.6	0.95	48	144	0.936
FDA	3x3	0.6	0.95	54	162	0.958
FDA	3x3	0.6	0.95	60	180	0.975
FDA	3x3	0.6	0.95	66	198	0.97
FDA	3x3	0.6	0.95	72	216	0.981
FDA	3x3	0.6	0.95	81	243	0.988
FDA	3x3	0.6	0.95	90	270	0.993
FDA	3x3	0.6	0.95	96	288	0.991
FDA	3x3	0.6	0.95	105	315	0.994

FDA	3x3	0.6	1	9	27	0.114
FDA	3x3	0.6	1	15	45	0.37
FDA	3x3	0.6	1	18	54	0.521
FDA	3x3	0.6	1	24	72	0.704
FDA	3x3	0.6	1	30	90	0.834
FDA	3x3	0.6	1	33	99	0.881
FDA	3x3	0.6	1	36	108	0.902
FDA	3x3	0.6	1	42	126	0.935
FDA	3x3	0.6	1	48	144	0.96
FDA	3x3	0.6	1	54	162	0.982
FDA	3x3	0.6	1	60	180	0.983
FDA	3x3	0.6	1	66	198	0.979
FDA	3x3	0.6	1	72	216	0.991
FDA	3x3	0.6	1	81	243	0.993
FDA	3x3	0.6	1	90	270	0.997
FDA	3x3	0.6	1	96	288	0.998
FDA	3x3	0.6	1	105	315	0.996
FDA	3x3	0.6	1.05	9	27	0.102
FDA	3x3	0.6	1.05	15	45	0.344
FDA	3x3	0.6	1.05	18	54	0.467
FDA	3x3	0.6	1.05	24	72	0.719
FDA	3x3	0.6	1.05	30	90	0.803
FDA	3x3	0.6	1.05	33	99	0.843
FDA	3x3	0.6	1.05	36	108	0.867
FDA	3x3	0.6	1.05	42	126	0.907
FDA	3x3	0.6	1.05	48	144	0.933
FDA	3x3	0.6	1.05	54	162	0.95
FDA	3x3	0.6	1.05	60	180	0.969
FDA	3x3	0.6	1.05	66	198	0.977
FDA	3x3	0.6	1.05	72	216	0.976
FDA	3x3	0.6	1.05	81	243	0.986
FDA	3x3	0.6	1.05	90	270	0.983
FDA	3x3	0.6	1.05	96	288	0.989
FDA	3x3	0.6	1.05	105	315	0.994
FDA	3x3	0.6	1.1	9	27	0.122
FDA	3x3	0.6	1.1	15	45	0.33
FDA	3x3	0.6	1.1	24	72	0.618
FDA	3x3	0.6	1.1	30	90	0.749
FDA	3x3	0.6	1.1	39	117	0.833
FDA	3x3	0.6	1.1	42	126	0.855
FDA	3x3	0.6	1.1	45	135	0.844

FDA	3x3	0.6	1.1	54	162	0.915
FDA	3x3	0.6	1.1	60	180	0.915
FDA	3x3	0.6	1.1	66	198	0.928
FDA	3x3	0.6	1.1	72	216	0.939
FDA	3x3	0.6	1.1	81	243	0.96
FDA	3x3	0.6	1.1	90	270	0.958
FDA	3x3	0.6	1.1	96	288	0.943
FDA	3x3	0.6	1.1	105	315	0.974
FDA	2x4	0.2	0.9	6	24	0.018
FDA	2x4	0.2	0.9	8	32	0.025
FDA	2x4	0.2	0.9	12	48	0.116
FDA	2x4	0.2	0.9	14	56	0.157
FDA	2x4	0.2	0.9	16	64	0.206
FDA	2x4	0.2	0.9	18	72	0.274
FDA	2x4	0.2	0.9	20	80	0.329
FDA	2x4	0.2	0.9	24	96	0.382
FDA	2x4	0.2	0.9	30	120	0.489
FDA	2x4	0.2	0.9	34	136	0.516
FDA	2x4	0.2	0.9	40	160	0.627
FDA	2x4	0.2	0.9	46	184	0.667
FDA	2x4	0.2	0.9	50	200	0.711
FDA	2x4	0.2	0.9	54	216	0.767
FDA	2x4	0.2	0.9	60	240	0.798
FDA	2x4	0.2	0.9	66	264	0.825
FDA	2x4	0.2	0.9	72	288	0.86
FDA	2x4	0.2	0.9	80	320	0.913
FDA	2x4	0.2	0.95	6	24	0.019
FDA	2x4	0.2	0.95	8	32	0.041
FDA	2x4	0.2	0.95	10	40	0.086
FDA	2x4	0.2	0.95	14	56	0.207
FDA	2x4	0.2	0.95	16	64	0.316
FDA	2x4	0.2	0.95	18	72	0.372
FDA	2x4	0.2	0.95	20	80	0.464
FDA	2x4	0.2	0.95	24	96	0.577
FDA	2x4	0.2	0.95	30	120	0.747
FDA	2x4	0.2	0.95	34	136	0.802
FDA	2x4	0.2	0.95	40	160	0.874
FDA	2x4	0.2	0.95	46	184	0.911
FDA	2x4	0.2	0.95	50	200	0.953
FDA	2x4	0.2	0.95	54	216	0.947
FDA	2x4	0.2	0.95	60	240	0.972

FDA	2x4	0.2	0.95	66	264	0.972
FDA	2x4	0.2	0.95	72	288	0.988
FDA	2x4	0.2	0.95	80	320	0.991
FDA	2x4	0.2	1	6	24	0.024
FDA	2x4	0.2	1	8	32	0.043
FDA	2x4	0.2	1	10	40	0.099
FDA	2x4	0.2	1	14	56	0.222
FDA	2x4	0.2	1	18	72	0.429
FDA	2x4	0.2	1	20	80	0.507
FDA	2x4	0.2	1	24	96	0.661
FDA	2x4	0.2	1	30	120	0.829
FDA	2x4	0.2	1	34	136	0.86
FDA	2x4	0.2	1	40	160	0.924
FDA	2x4	0.2	1	46	184	0.953
FDA	2x4	0.2	1	50	200	0.981
FDA	2x4	0.2	1	54	216	0.983
FDA	2x4	0.2	1	60	240	0.995
FDA	2x4	0.2	1	66	264	0.996
FDA	2x4	0.2	1	72	288	0.999
FDA	2x4	0.2	1	80	320	0.999
FDA	2x4	0.2	1.05	6	24	0.023
FDA	2x4	0.2	1.05	8	32	0.047
FDA	2x4	0.2	1.05	12	48	0.144
FDA	2x4	0.2	1.05	14	56	0.203
FDA	2x4	0.2	1.05	16	64	0.29
FDA	2x4	0.2	1.05	18	72	0.36
FDA	2x4	0.2	1.05	20	80	0.446
FDA	2x4	0.2	1.05	24	96	0.577
FDA	2x4	0.2	1.05	30	120	0.732
FDA	2x4	0.2	1.05	34	136	0.744
FDA	2x4	0.2	1.05	40	160	0.813
FDA	2x4	0.2	1.05	46	184	0.89
FDA	2x4	0.2	1.05	50	200	0.896
FDA	2x4	0.2	1.05	54	216	0.905
FDA	2x4	0.2	1.05	60	240	0.931
FDA	2x4	0.2	1.05	66	264	0.968
FDA	2x4	0.2	1.05	72	288	0.97
FDA	2x4	0.2	1.05	80	320	0.977
FDA	2x4	0.2	1.1	6	24	0.019
FDA	2x4	0.2	1.1	8	32	0.035
FDA	2x4	0.2	1.1	10	40	0.074

FDA	2x4	0.2	1.1	14	56	0.152
FDA	2x4	0.2	1.1	16	64	0.206
FDA	2x4	0.2	1.1	18	72	0.28
FDA	2x4	0.2	1.1	20	80	0.307
FDA	2x4	0.2	1.1	22	88	0.346
FDA	2x4	0.2	1.1	30	120	0.483
FDA	2x4	0.2	1.1	34	136	0.512
FDA	2x4	0.2	1.1	40	160	0.591
FDA	2x4	0.2	1.1	46	184	0.682
FDA	2x4	0.2	1.1	50	200	0.722
FDA	2x4	0.2	1.1	54	216	0.731
FDA	2x4	0.2	1.1	60	240	0.753
FDA	2x4	0.2	1.1	66	264	0.816
FDA	2x4	0.2	1.1	72	288	0.838
FDA	2x4	0.2	1.1	80	320	0.869
FDA	2x4	0.3	0.9	6	24	0.022
FDA	2x4	0.3	0.9	12	48	0.12
FDA	2x4	0.3	0.9	18	72	0.251
FDA	2x4	0.3	0.9	24	96	0.424
FDA	2x4	0.3	0.9	30	120	0.509
FDA	2x4	0.3	0.9	36	144	0.59
FDA	2x4	0.3	0.9	40	160	0.6
FDA	2x4	0.3	0.9	44	176	0.644
FDA	2x4	0.3	0.9	50	200	0.694
FDA	2x4	0.3	0.9	54	216	0.75
FDA	2x4	0.3	0.9	60	240	0.784
FDA	2x4	0.3	0.9	66	264	0.813
FDA	2x4	0.3	0.9	72	288	0.853
FDA	2x4	0.3	0.9	80	320	0.89
FDA	2x4	0.3	0.95	6	24	0.034
FDA	2x4	0.3	0.95	8	32	0.064
FDA	2x4	0.3	0.95	12	48	0.194
FDA	2x4	0.3	0.95	14	56	0.222
FDA	2x4	0.3	0.95	16	64	0.296
FDA	2x4	0.3	0.95	18	72	0.341
FDA	2x4	0.3	0.95	20	80	0.47
FDA	2x4	0.3	0.95	26	104	0.626
FDA	2x4	0.3	0.95	30	120	0.712
FDA	2x4	0.3	0.95	36	144	0.825
FDA	2x4	0.3	0.95	40	160	0.83
FDA	2x4	0.3	0.95	44	176	0.875

FDA	2x4	0.3	0.95	50	200	0.905
FDA	2x4	0.3	0.95	54	216	0.926
FDA	2x4	0.3	0.95	60	240	0.958
FDA	2x4	0.3	0.95	66	264	0.958
FDA	2x4	0.3	0.95	72	288	0.974
FDA	2x4	0.3	0.95	80	320	0.982
FDA	2x4	0.3	1	6	24	0.041
FDA	2x4	0.3	1	8	32	0.058
FDA	2x4	0.3	1	12	48	0.15
FDA	2x4	0.3	1	14	56	0.234
FDA	2x4	0.3	1	16	64	0.308
FDA	2x4	0.3	1	18	72	0.399
FDA	2x4	0.3	1	20	80	0.471
FDA	2x4	0.3	1	22	88	0.557
FDA	2x4	0.3	1	30	120	0.764
FDA	2x4	0.3	1	36	144	0.855
FDA	2x4	0.3	1	40	160	0.89
FDA	2x4	0.3	1	44	176	0.93
FDA	2x4	0.3	1	50	200	0.949
FDA	2x4	0.3	1	54	216	0.965
FDA	2x4	0.3	1	60	240	0.982
FDA	2x4	0.3	1	66	264	0.992
FDA	2x4	0.3	1	72	288	0.993
FDA	2x4	0.3	1	80	320	0.996
FDA	2x4	0.3	1.05	6	24	0.031
FDA	2x4	0.3	1.05	8	32	0.053
FDA	2x4	0.3	1.05	12	48	0.169
FDA	2x4	0.3	1.05	14	56	0.227
FDA	2x4	0.3	1.05	16	64	0.29
FDA	2x4	0.3	1.05	18	72	0.372
FDA	2x4	0.3	1.05	20	80	0.405
FDA	2x4	0.3	1.05	24	96	0.58
FDA	2x4	0.3	1.05	26	104	0.615
FDA	2x4	0.3	1.05	30	120	0.714
FDA	2x4	0.3	1.05	36	144	0.779
FDA	2x4	0.3	1.05	40	160	0.815
FDA	2x4	0.3	1.05	44	176	0.854
FDA	2x4	0.3	1.05	50	200	0.896
FDA	2x4	0.3	1.05	54	216	0.903
FDA	2x4	0.3	1.05	60	240	0.922
FDA	2x4	0.3	1.05	66	264	0.943

FDA	2x4	0.3	1.05	72	288	0.962
FDA	2x4	0.3	1.05	80	320	0.974
FDA	2x4	0.3	1.1	6	24	0.031
FDA	2x4	0.3	1.1	12	48	0.136
FDA	2x4	0.3	1.1	18	72	0.303
FDA	2x4	0.3	1.1	24	96	0.408
FDA	2x4	0.3	1.1	30	120	0.532
FDA	2x4	0.3	1.1	34	136	0.569
FDA	2x4	0.3	1.1	38	152	0.602
FDA	2x4	0.3	1.1	42	168	0.668
FDA	2x4	0.3	1.1	46	184	0.692
FDA	2x4	0.3	1.1	50	200	0.717
FDA	2x4	0.3	1.1	36	144	0.592
FDA	2x4	0.3	1.1	40	160	0.629
FDA	2x4	0.3	1.1	44	176	0.685
FDA	2x4	0.3	1.1	50	200	0.696
FDA	2x4	0.3	1.1	54	216	0.744
FDA	2x4	0.3	1.1	60	240	0.794
FDA	2x4	0.3	1.1	66	264	0.796
FDA	2x4	0.3	1.1	72	288	0.831
FDA	2x4	0.3	1.1	80	320	0.89
FDA	2x4	0.4	0.9	6	24	0.039
FDA	2x4	0.4	0.9	12	48	0.213
FDA	2x4	0.4	0.9	18	72	0.437
FDA	2x4	0.4	0.9	24	96	0.589
FDA	2x4	0.4	0.9	28	112	0.677
FDA	2x4	0.4	0.9	32	128	0.752
FDA	2x4	0.4	0.9	34	136	0.746
FDA	2x4	0.4	0.9	38	152	0.804
FDA	2x4	0.4	0.9	42	168	0.847
FDA	2x4	0.4	0.9	48	192	0.885
FDA	2x4	0.4	0.9	54	216	0.926
FDA	2x4	0.4	0.9	60	240	0.942
FDA	2x4	0.4	0.9	66	264	0.944
FDA	2x4	0.4	0.9	72	288	0.974
FDA	2x4	0.4	0.9	80	320	0.976
FDA	2x4	0.4	0.95	6	24	0.06
FDA	2x4	0.4	0.95	12	48	0.261
FDA	2x4	0.4	0.95	18	72	0.51
FDA	2x4	0.4	0.95	20	80	0.608
FDA	2x4	0.4	0.95	22	88	0.649

FDA	2x4	0.4	0.95	26	104	0.76
FDA	2x4	0.4	0.95	28	112	0.787
FDA	2x4	0.4	0.95	32	128	0.872
FDA	2x4	0.4	0.95	34	136	0.874
FDA	2x4	0.4	0.95	38	152	0.916
FDA	2x4	0.4	0.95	42	168	0.939
FDA	2x4	0.4	0.95	48	192	0.968
FDA	2x4	0.4	0.95	54	216	0.981
FDA	2x4	0.4	0.95	60	240	0.989
FDA	2x4	0.4	0.95	66	264	0.996
FDA	2x4	0.4	0.95	72	288	0.995
FDA	2x4	0.4	0.95	80	320	0.998
FDA	2x4	0.4	1	6	24	0.058
FDA	2x4	0.4	1	12	48	0.265
FDA	2x4	0.4	1	18	72	0.557
FDA	2x4	0.4	1	20	80	0.638
FDA	2x4	0.4	1	24	96	0.75
FDA	2x4	0.4	1	28	112	0.827
FDA	2x4	0.4	1	32	128	0.871
FDA	2x4	0.4	1	34	136	0.909
FDA	2x4	0.4	1	38	152	0.944
FDA	2x4	0.4	1	42	168	0.966
FDA	2x4	0.4	1	48	192	0.986
FDA	2x4	0.4	1	54	216	0.989
FDA	2x4	0.4	1	60	240	0.998
FDA	2x4	0.4	1	66	264	1
FDA	2x4	0.4	1	72	288	1
FDA	2x4	0.4	1	80	320	1
FDA	2x4	0.4	1.05	6	24	0.06
FDA	2x4	0.4	1.05	12	48	0.247
FDA	2x4	0.4	1.05	18	72	0.518
FDA	2x4	0.4	1.05	20	80	0.567
FDA	2x4	0.4	1.05	22	88	0.636
FDA	2x4	0.4	1.05	26	104	0.751
FDA	2x4	0.4	1.05	30	120	0.824
FDA	2x4	0.4	1.05	32	128	0.849
FDA	2x4	0.4	1.05	34	136	0.881
FDA	2x4	0.4	1.05	38	152	0.918
FDA	2x4	0.4	1.05	42	168	0.945
FDA	2x4	0.4	1.05	48	192	0.962
FDA	2x4	0.4	1.05	54	216	0.967

FDA	2x4	0.4	1.05	60	240	0.992
FDA	2x4	0.4	1.05	66	264	0.988
FDA	2x4	0.4	1.05	72	288	0.997
FDA	2x4	0.4	1.05	80	320	0.998
FDA	2x4	0.4	1.1	6	24	0.041
FDA	2x4	0.4	1.1	12	48	0.226
FDA	2x4	0.4	1.1	18	72	0.424
FDA	2x4	0.4	1.1	24	96	0.59
FDA	2x4	0.4	1.1	28	112	0.665
FDA	2x4	0.4	1.1	32	128	0.73
FDA	2x4	0.4	1.1	36	144	0.789
FDA	2x4	0.4	1.1	40	160	0.834
FDA	2x4	0.4	1.1	44	176	0.864
FDA	2x4	0.4	1.1	48	192	0.899
FDA	2x4	0.4	1.1	54	216	0.934
FDA	2x4	0.4	1.1	60	240	0.958
FDA	2x4	0.4	1.1	66	264	0.964
FDA	2x4	0.4	1.1	72	288	0.966
FDA	2x4	0.4	1.1	80	320	0.976
FDA	2x4	0.5	0.9	6	24	0.071
FDA	2x4	0.5	0.9	12	48	0.308
FDA	2x4	0.5	0.9	18	72	0.561
FDA	2x4	0.5	0.9	24	96	0.688
FDA	2x4	0.5	0.9	28	112	0.766
FDA	2x4	0.5	0.9	32	128	0.831
FDA	2x4	0.5	0.9	36	144	0.853
FDA	2x4	0.5	0.9	40	160	0.888
FDA	2x4	0.5	0.9	44	176	0.901
FDA	2x4	0.5	0.9	48	192	0.922
FDA	2x4	0.5	0.9	54	216	0.94
FDA	2x4	0.5	0.9	60	240	0.957
FDA	2x4	0.5	0.9	66	264	0.971
FDA	2x4	0.5	0.9	72	288	0.986
FDA	2x4	0.5	0.9	80	320	0.983
FDA	2x4	0.5	0.95	6	24	0.067
FDA	2x4	0.5	0.95	12	48	0.349
FDA	2x4	0.5	0.95	18	72	0.611
FDA	2x4	0.5	0.95	22	88	0.739
FDA	2x4	0.5	0.95	26	104	0.819
FDA	2x4	0.5	0.95	28	112	0.871
FDA	2x4	0.5	0.95	32	128	0.911

FDA	2x4	0.5	0.95	36	144	0.937
FDA	2x4	0.5	0.95	40	160	0.96
FDA	2x4	0.5	0.95	44	176	0.966
FDA	2x4	0.5	0.95	48	192	0.971
FDA	2x4	0.5	0.95	54	216	0.986
FDA	2x4	0.5	0.95	60	240	0.99
FDA	2x4	0.5	0.95	66	264	0.994
FDA	2x4	0.5	0.95	72	288	0.996
FDA	2x4	0.5	0.95	80	320	0.999
FDA	2x4	0.5	1	6	24	0.075
FDA	2x4	0.5	1	12	48	0.353
FDA	2x4	0.5	1	18	72	0.653
FDA	2x4	0.5	1	20	80	0.698
FDA	2x4	0.5	1	24	96	0.827
FDA	2x4	0.5	1	26	104	0.844
FDA	2x4	0.5	1	28	112	0.882
FDA	2x4	0.5	1	32	128	0.924
FDA	2x4	0.5	1	36	144	0.944
FDA	2x4	0.5	1	40	160	0.976
FDA	2x4	0.5	1	44	176	0.987
FDA	2x4	0.5	1	48	192	0.988
FDA	2x4	0.5	1	54	216	0.996
FDA	2x4	0.5	1	60	240	0.998
FDA	2x4	0.5	1	66	264	0.995
FDA	2x4	0.5	1	72	288	1
FDA	2x4	0.5	1	80	320	0.999
FDA	2x4	0.5	1.05	6	24	0.068
FDA	2x4	0.5	1.05	12	48	0.361
FDA	2x4	0.5	1.05	18	72	0.615
FDA	2x4	0.5	1.05	22	88	0.735
FDA	2x4	0.5	1.05	24	96	0.776
FDA	2x4	0.5	1.05	28	112	0.85
FDA	2x4	0.5	1.05	32	128	0.892
FDA	2x4	0.5	1.05	36	144	0.922
FDA	2x4	0.5	1.05	40	160	0.946
FDA	2x4	0.5	1.05	44	176	0.965
FDA	2x4	0.5	1.05	48	192	0.977
FDA	2x4	0.5	1.05	54	216	0.982
FDA	2x4	0.5	1.05	60	240	0.989
FDA	2x4	0.5	1.05	66	264	0.994
FDA	2x4	0.5	1.05	72	288	0.996

FDA	2x4	0.5	1.05	80	320	0.996
FDA	2x4	0.5	1.1	6	24	0.077
FDA	2x4	0.5	1.1	12	48	0.283
FDA	2x4	0.5	1.1	18	72	0.531
FDA	2x4	0.5	1.1	22	88	0.641
FDA	2x4	0.5	1.1	28	112	0.789
FDA	2x4	0.5	1.1	34	136	0.851
FDA	2x4	0.5	1.1	36	144	0.867
FDA	2x4	0.5	1.1	38	152	0.889
FDA	2x4	0.5	1.1	40	160	0.896
FDA	2x4	0.5	1.1	44	176	0.916
FDA	2x4	0.5	1.1	48	192	0.922
FDA	2x4	0.5	1.1	54	216	0.946
FDA	2x4	0.5	1.1	60	240	0.956
FDA	2x4	0.5	1.1	66	264	0.969
FDA	2x4	0.5	1.1	72	288	0.978
FDA	2x4	0.5	1.1	80	320	0.979
FDA	2x4	0.6	0.9	6	24	0.085
FDA	2x4	0.6	0.9	10	40	0.232
FDA	2x4	0.6	0.9	14	56	0.438
FDA	2x4	0.6	0.9	18	72	0.597
FDA	2x4	0.6	0.9	22	88	0.709
FDA	2x4	0.6	0.9	26	104	0.757
FDA	2x4	0.6	0.9	32	128	0.858
FDA	2x4	0.6	0.9	36	144	0.886
FDA	2x4	0.6	0.9	40	160	0.894
FDA	2x4	0.6	0.9	46	184	0.922
FDA	2x4	0.6	0.9	50	200	0.925
FDA	2x4	0.6	0.9	54	216	0.952
FDA	2x4	0.6	0.9	60	240	0.943
FDA	2x4	0.6	0.9	66	264	0.945
FDA	2x4	0.6	0.9	72	288	0.967
FDA	2x4	0.6	0.9	80	320	0.978
FDA	2x4	0.6	0.95	6	24	0.081
FDA	2x4	0.6	0.95	10	40	0.264
FDA	2x4	0.6	0.95	14	56	0.497
FDA	2x4	0.6	0.95	18	72	0.647
FDA	2x4	0.6	0.95	22	88	0.772
FDA	2x4	0.6	0.95	26	104	0.857
FDA	2x4	0.6	0.95	30	120	0.903
FDA	2x4	0.6	0.95	32	128	0.916

FDA	2x4	0.6	0.95	36	144	0.939
FDA	2x4	0.6	0.95	40	160	0.963
FDA	2x4	0.6	0.95	46	184	0.973
FDA	2x4	0.6	0.95	50	200	0.989
FDA	2x4	0.6	0.95	54	216	0.985
FDA	2x4	0.6	0.95	60	240	0.99
FDA	2x4	0.6	0.95	66	264	0.993
FDA	2x4	0.6	0.95	72	288	0.991
FDA	2x4	0.6	0.95	80	320	0.995
FDA	2x4	0.6	1	6	24	0.11
FDA	2x4	0.6	1	10	40	0.277
FDA	2x4	0.6	1	14	56	0.492
FDA	2x4	0.6	1	18	72	0.668
FDA	2x4	0.6	1	22	88	0.805
FDA	2x4	0.6	1	24	96	0.822
FDA	2x4	0.6	1	26	104	0.879
FDA	2x4	0.6	1	30	120	0.917
FDA	2x4	0.6	1	36	144	0.965
FDA	2x4	0.6	1	40	160	0.969
FDA	2x4	0.6	1	46	184	0.982
FDA	2x4	0.6	1	50	200	0.993
FDA	2x4	0.6	1	54	216	0.993
FDA	2x4	0.6	1	60	240	0.994
FDA	2x4	0.6	1	66	264	0.998
FDA	2x4	0.6	1	72	288	1
FDA	2x4	0.6	1	80	320	0.999
FDA	2x4	0.6	1.05	6	24	0.078
FDA	2x4	0.6	1.05	10	40	0.252
FDA	2x4	0.6	1.05	14	56	0.473
FDA	2x4	0.6	1.05	18	72	0.648
FDA	2x4	0.6	1.05	22	88	0.761
FDA	2x4	0.6	1.05	24	96	0.786
FDA	2x4	0.6	1.05	26	104	0.836
FDA	2x4	0.6	1.05	32	128	0.917
FDA	2x4	0.6	1.05	36	144	0.933
FDA	2x4	0.6	1.05	40	160	0.955
FDA	2x4	0.6	1.05	46	184	0.955
FDA	2x4	0.6	1.05	50	200	0.968
FDA	2x4	0.6	1.05	54	216	0.975
FDA	2x4	0.6	1.05	60	240	0.98
FDA	2x4	0.6	1.05	66	264	0.984

FDA	2x4	0.6	1.05	72	288	0.99
FDA	2x4	0.6	1.05	80	320	0.995
FDA	2x4	0.6	1.1	6	24	0.068
FDA	2x4	0.6	1.1	12	48	0.355
FDA	2x4	0.6	1.1	18	72	0.613
FDA	2x4	0.6	1.1	24	96	0.719
FDA	2x4	0.6	1.1	30	120	0.815
FDA	2x4	0.6	1.1	32	128	0.832
FDA	2x4	0.6	1.1	36	144	0.877
FDA	2x4	0.6	1.1	42	168	0.888
FDA	2x4	0.6	1.1	46	184	0.916
FDA	2x4	0.6	1.1	50	200	0.936
FDA	2x4	0.6	1.1	54	216	0.934
FDA	2x4	0.6	1.1	60	240	0.939
FDA	2x4	0.6	1.1	66	264	0.951
FDA	2x4	0.6	1.1	72	288	0.954
FDA	2x4	0.6	1.1	80	320	0.983
