# Supplementary material for: Model-Based Approach for Designing an Efficient Bioequivalence Study for Highly Variable Drugs
Source: Pharmaceuticals (Basel). 2021 Oct 28;14(11):1101. doi: 10.3390/ph14111101 (PMC8624447; doi:10.3390/ph14111101)
Supplement: Supplementary file 1 [file pharmaceuticals-14-01101-s001.zip › [Supplementary File S3] The results for Figure 5.pdf]

Table S3. Comparison of power among clinical trial designs by correlation coefficient of bioavailability (F) between reference and test drugs: The simulation results are summarized in Figure 5.

Agency	Correlation	Design	CV	GMR	Total number of subject	Total number of hospitalization (N)	Power
EMA	0.7	2x2	0.3	1	12	24	0.002
EMA	0.7	2x2	0.3	1	16	32	0.004
EMA	0.7	2x2	0.3	1	24	48	0.039
EMA	0.7	2x2	0.3	1	28	56	0.077
EMA	0.7	2x2	0.3	1	32	64	0.136
EMA	0.7	2x2	0.3	1	36	72	0.183
EMA	0.7	2x2	0.3	1	40	80	0.257
EMA	0.7	2x2	0.3	1	44	88	0.303
EMA	0.7	2x2	0.3	1	60	120	0.56
EMA	0.7	2x2	0.3	1	72	144	0.717
EMA	0.7	2x2	0.3	1	80	160	0.771
EMA	0.7	2x2	0.3	1	88	176	0.823
EMA	0.7	2x2	0.3	1	100	200	0.864
EMA	0.7	2x2	0.3	1	108	216	0.912
EMA	0.7	2x2	0.3	1	120	240	0.945
EMA	0.7	2x2	0.3	1	132	264	0.959
EMA	0.7	2x2	0.3	1	144	288	0.969
EMA	0.7	2x2	0.3	1	160	320	0.983
EMA	0.7	3x3	0.3	1	9	27	0.002
EMA	0.7	3x3	0.3	1	12	36	0.019
EMA	0.7	3x3	0.3	1	15	45	0.039
EMA	0.7	3x3	0.3	1	18	54	0.081
EMA	0.7	3x3	0.3	1	21	63	0.18
EMA	0.7	3x3	0.3	1	24	72	0.232
EMA	0.7	3x3	0.3	1	27	81	0.333
EMA	0.7	3x3	0.3	1	30	90	0.374
EMA	0.7	3x3	0.3	1	39	117	0.56
EMA	0.7	3x3	0.3	1	48	144	0.696
EMA	0.7	3x3	0.3	1	54	162	0.762
EMA	0.7	3x3	0.3	1	60	180	0.826
EMA	0.7	3x3	0.3	1	66	198	0.856
EMA	0.7	3x3	0.3	1	75	225	0.903
EMA	0.7	3x3	0.3	1	81	243	0.935
EMA	0.7	3x3	0.3	1	90	270	0.945
EMA	0.7	3x3	0.3	1	96	288	0.951
EMA	0.7	3x3	0.3	1	105	315	0.96
EMA	0.7	2x4	0.3	1	6	24	0.003

EMA	0.7	2x4	0.3	1	8	32	0.022
EMA	0.7	2x4	0.3	1	12	48	0.072
EMA	0.7	2x4	0.3	1	14	56	0.143
EMA	0.7	2x4	0.3	1	16	64	0.201
EMA	0.7	2x4	0.3	1	18	72	0.269
EMA	0.7	2x4	0.3	1	20	80	0.329
EMA	0.7	2x4	0.3	1	22	88	0.383
EMA	0.7	2x4	0.3	1	30	120	0.601
EMA	0.7	2x4	0.3	1	36	144	0.714
EMA	0.7	2x4	0.3	1	40	160	0.755
EMA	0.7	2x4	0.3	1	44	176	0.803
EMA	0.7	2x4	0.3	1	50	200	0.831
EMA	0.7	2x4	0.3	1	54	216	0.884
EMA	0.7	2x4	0.3	1	60	240	0.911
EMA	0.7	2x4	0.3	1	66	264	0.924
EMA	0.7	2x4	0.3	1	72	288	0.952
EMA	0.7	2x4	0.3	1	80	320	0.968
EMA	0.9	2x2	0.3	1	12	24	0.018
EMA	0.9	2x2	0.3	1	16	32	0.044
EMA	0.9	2x2	0.3	1	24	48	0.228
EMA	0.9	2x2	0.3	1	28	56	0.338
EMA	0.9	2x2	0.3	1	32	64	0.445
EMA	0.9	2x2	0.3	1	36	72	0.53
EMA	0.9	2x2	0.3	1	40	80	0.61
EMA	0.9	2x2	0.3	1	44	88	0.662
EMA	0.9	2x2	0.3	1	60	120	0.834
EMA	0.9	2x2	0.3	1	72	144	0.921
EMA	0.9	2x2	0.3	1	80	160	0.948
EMA	0.9	2x2	0.3	1	88	176	0.978
EMA	0.9	2x2	0.3	1	100	200	0.987
EMA	0.9	2x2	0.3	1	108	216	0.992
EMA	0.9	2x2	0.3	1	120	240	0.995
EMA	0.9	2x2	0.3	1	132	264	0.999
EMA	0.9	2x2	0.3	1	144	288	0.999
EMA	0.9	2x2	0.3	1	160	320	0.999
EMA	0.9	3x3	0.3	1	9	27	0.018
EMA	0.9	3x3	0.3	1	12	36	0.089
EMA	0.9	3x3	0.3	1	15	45	0.206
EMA	0.9	3x3	0.3	1	18	54	0.321
EMA	0.9	3x3	0.3	1	21	63	0.43
EMA	0.9	3x3	0.3	1	24	72	0.507

EMA	0.9	3x3	0.3	1	27	81	0.628
EMA	0.9	3x3	0.3	1	30	90	0.689
EMA	0.9	3x3	0.3	1	39	117	0.785
EMA	0.9	3x3	0.3	1	48	144	0.912
EMA	0.9	3x3	0.3	1	54	162	0.926
EMA	0.9	3x3	0.3	1	60	180	0.966
EMA	0.9	3x3	0.3	1	66	198	0.977
EMA	0.9	3x3	0.3	1	75	225	0.989
EMA	0.9	3x3	0.3	1	81	243	0.994
EMA	0.9	3x3	0.3	1	90	270	0.995
EMA	0.9	3x3	0.3	1	96	288	0.998
EMA	0.9	3x3	0.3	1	105	315	0.997
EMA	0.9	2x4	0.3	1	6	24	0.024
EMA	0.9	2x4	0.3	1	8	32	0.081
EMA	0.9	2x4	0.3	1	12	48	0.263
EMA	0.9	2x4	0.3	1	14	56	0.402
EMA	0.9	2x4	0.3	1	16	64	0.473
EMA	0.9	2x4	0.3	1	18	72	0.554
EMA	0.9	2x4	0.3	1	20	80	0.64
EMA	0.9	2x4	0.3	1	22	88	0.684
EMA	0.9	2x4	0.3	1	30	120	0.833
EMA	0.9	2x4	0.3	1	36	144	0.902
EMA	0.9	2x4	0.3	1	40	160	0.939
EMA	0.9	2x4	0.3	1	44	176	0.964
EMA	0.9	2x4	0.3	1	50	200	0.977
EMA	0.9	2x4	0.3	1	54	216	0.988
EMA	0.9	2x4	0.3	1	60	240	0.987
EMA	0.9	2x4	0.3	1	66	264	0.999
EMA	0.9	2x4	0.3	1	72	288	0.998
EMA	0.9	2x4	0.3	1	80	320	0.999
EMA	1	2x2	0.3	1	12	24	0.071
EMA	1	2x2	0.3	1	16	32	0.154
EMA	1	2x2	0.3	1	24	48	0.437
EMA	1	2x2	0.3	1	28	56	0.592
EMA	1	2x2	0.3	1	32	64	0.702
EMA	1	2x2	0.3	1	36	72	0.77
EMA	1	2x2	0.3	1	40	80	0.829
EMA	1	2x2	0.3	1	44	88	0.862
EMA	1	2x2	0.3	1	60	120	0.967
EMA	1	2x2	0.3	1	72	144	0.987
EMA	1	2x2	0.3	1	80	160	0.994

EMA	1	2x2	0.3	1	88	176	0.996
EMA	1	2x2	0.3	1	100	200	0.998
EMA	1	2x2	0.3	1	108	216	0.999
EMA	1	2x2	0.3	1	120	240	0.999
EMA	1	2x2	0.3	1	132	264	1
EMA	1	2x2	0.3	1	144	288	1
EMA	1	2x2	0.3	1	160	320	1
EMA	1	3x3	0.3	1	9	27	0.07
EMA	1	3x3	0.3	1	12	36	0.209
EMA	1	3x3	0.3	1	15	45	0.403
EMA	1	3x3	0.3	1	18	54	0.524
EMA	1	3x3	0.3	1	21	63	0.641
EMA	1	3x3	0.3	1	24	72	0.735
EMA	1	3x3	0.3	1	27	81	0.822
EMA	1	3x3	0.3	1	30	90	0.866
EMA	1	3x3	0.3	1	39	117	0.945
EMA	1	3x3	0.3	1	48	144	0.974
EMA	1	3x3	0.3	1	54	162	0.992
EMA	1	3x3	0.3	1	60	180	0.996
EMA	1	3x3	0.3	1	66	198	0.996
EMA	1	3x3	0.3	1	75	225	1
EMA	1	3x3	0.3	1	81	243	1
EMA	1	3x3	0.3	1	90	270	1
EMA	1	3x3	0.3	1	96	288	1
EMA	1	3x3	0.3	1	105	315	1
EMA	1	2x4	0.3	1	6	24	0.084
EMA	1	2x4	0.3	1	8	32	0.205
EMA	1	2x4	0.3	1	12	48	0.509
EMA	1	2x4	0.3	1	14	56	0.639
EMA	1	2x4	0.3	1	16	64	0.736
EMA	1	2x4	0.3	1	18	72	0.807
EMA	1	2x4	0.3	1	20	80	0.85
EMA	1	2x4	0.3	1	22	88	0.869
EMA	1	2x4	0.3	1	30	120	0.979
EMA	1	2x4	0.3	1	36	144	0.986
EMA	1	2x4	0.3	1	40	160	0.995
EMA	1	2x4	0.3	1	44	176	0.999
EMA	1	2x4	0.3	1	50	200	0.998
EMA	1	2x4	0.3	1	54	216	1
EMA	1	2x4	0.3	1	60	240	1
EMA	1	2x4	0.3	1	66	264	1

EMA	1	2x4	0.3	1	72	288	1
EMA	1	2x4	0.3	1	80	320	1
FDA	0.7	2x2	0.3	1	12	24	0.002
FDA	0.7	2x2	0.3	1	16	32	0.004
FDA	0.7	2x2	0.3	1	24	48	0.039
FDA	0.7	2x2	0.3	1	28	56	0.077
FDA	0.7	2x2	0.3	1	32	64	0.136
FDA	0.7	2x2	0.3	1	36	72	0.183
FDA	0.7	2x2	0.3	1	40	80	0.257
FDA	0.7	2x2	0.3	1	44	88	0.303
FDA	0.7	2x2	0.3	1	60	120	0.56
FDA	0.7	2x2	0.3	1	72	144	0.717
FDA	0.7	2x2	0.3	1	80	160	0.771
FDA	0.7	2x2	0.3	1	88	176	0.823
FDA	0.7	2x2	0.3	1	100	200	0.864
FDA	0.7	2x2	0.3	1	108	216	0.912
FDA	0.7	2x2	0.3	1	120	240	0.945
FDA	0.7	2x2	0.3	1	132	264	0.959
FDA	0.7	2x2	0.3	1	144	288	0.969
FDA	0.7	2x2	0.3	1	160	320	0.983
FDA	0.7	3x3	0.3	1	9	27	0.012
FDA	0.7	3x3	0.3	1	12	36	0.036
FDA	0.7	3x3	0.3	1	15	45	0.07
FDA	0.7	3x3	0.3	1	18	54	0.12
FDA	0.7	3x3	0.3	1	21	63	0.204
FDA	0.7	3x3	0.3	1	24	72	0.248
FDA	0.7	3x3	0.3	1	27	81	0.357
FDA	0.7	3x3	0.3	1	30	90	0.395
FDA	0.7	3x3	0.3	1	39	117	0.592
FDA	0.7	3x3	0.3	1	48	144	0.728
FDA	0.7	3x3	0.3	1	54	162	0.795
FDA	0.7	3x3	0.3	1	60	180	0.852
FDA	0.7	3x3	0.3	1	66	198	0.877
FDA	0.7	3x3	0.3	1	75	225	0.915
FDA	0.7	3x3	0.3	1	81	243	0.948
FDA	0.7	3x3	0.3	1	90	270	0.957
FDA	0.7	3x3	0.3	1	96	288	0.958
FDA	0.7	3x3	0.3	1	105	315	0.973
FDA	0.7	2x4	0.3	1	6	24	0.005
FDA	0.7	2x4	0.3	1	8	32	0.017
FDA	0.7	2x4	0.3	1	12	48	0.028

FDA	0.7	2x4	0.3	1	14	56	0.048
FDA	0.7	2x4	0.3	1	16	64	0.058
FDA	0.7	2x4	0.3	1	18	72	0.105
FDA	0.7	2x4	0.3	1	20	80	0.122
FDA	0.7	2x4	0.3	1	22	88	0.176
FDA	0.7	2x4	0.3	1	30	120	0.325
FDA	0.7	2x4	0.3	1	36	144	0.504
FDA	0.7	2x4	0.3	1	40	160	0.581
FDA	0.7	2x4	0.3	1	44	176	0.621
FDA	0.7	2x4	0.3	1	50	200	0.687
FDA	0.7	2x4	0.3	1	54	216	0.753
FDA	0.7	2x4	0.3	1	60	240	0.82
FDA	0.7	2x4	0.3	1	66	264	0.858
FDA	0.7	2x4	0.3	1	72	288	0.901
FDA	0.7	2x4	0.3	1	80	320	0.91
FDA	0.9	2x2	0.3	1	12	24	0.018
FDA	0.9	2x2	0.3	1	16	32	0.044
FDA	0.9	2x2	0.3	1	24	48	0.228
FDA	0.9	2x2	0.3	1	28	56	0.338
FDA	0.9	2x2	0.3	1	32	64	0.445
FDA	0.9	2x2	0.3	1	36	72	0.53
FDA	0.9	2x2	0.3	1	40	80	0.61
FDA	0.9	2x2	0.3	1	44	88	0.662
FDA	0.9	2x2	0.3	1	60	120	0.834
FDA	0.9	2x2	0.3	1	72	144	0.921
FDA	0.9	2x2	0.3	1	80	160	0.948
FDA	0.9	2x2	0.3	1	88	176	0.978
FDA	0.9	2x2	0.3	1	100	200	0.987
FDA	0.9	2x2	0.3	1	108	216	0.992
FDA	0.9	2x2	0.3	1	120	240	0.995
FDA	0.9	2x2	0.3	1	132	264	0.999
FDA	0.9	2x2	0.3	1	144	288	0.999
FDA	0.9	2x2	0.3	1	160	320	0.999
FDA	0.9	3x3	0.3	1	9	27	0.07
FDA	0.9	3x3	0.3	1	12	36	0.145
FDA	0.9	3x3	0.3	1	15	45	0.276
FDA	0.9	3x3	0.3	1	18	54	0.389
FDA	0.9	3x3	0.3	1	21	63	0.494
FDA	0.9	3x3	0.3	1	24	72	0.579
FDA	0.9	3x3	0.3	1	27	81	0.677
FDA	0.9	3x3	0.3	1	30	90	0.749

FDA	0.9	3x3	0.3	1	39	117	0.832
FDA	0.9	3x3	0.3	1	48	144	0.937
FDA	0.9	3x3	0.3	1	54	162	0.943
FDA	0.9	3x3	0.3	1	60	180	0.977
FDA	0.9	3x3	0.3	1	66	198	0.985
FDA	0.9	3x3	0.3	1	75	225	0.992
FDA	0.9	3x3	0.3	1	81	243	0.995
FDA	0.9	3x3	0.3	1	90	270	0.995
FDA	0.9	3x3	0.3	1	96	288	0.999
FDA	0.9	3x3	0.3	1	105	315	0.998
FDA	0.9	2x4	0.3	1	6	24	0.041
FDA	0.9	2x4	0.3	1	8	32	0.058
FDA	0.9	2x4	0.3	1	12	48	0.15
FDA	0.9	2x4	0.3	1	14	56	0.234
FDA	0.9	2x4	0.3	1	16	64	0.308
FDA	0.9	2x4	0.3	1	18	72	0.399
FDA	0.9	2x4	0.3	1	20	80	0.471
FDA	0.9	2x4	0.3	1	22	88	0.557
FDA	0.9	2x4	0.3	1	30	120	0.764
FDA	0.9	2x4	0.3	1	36	144	0.855
FDA	0.9	2x4	0.3	1	40	160	0.89
FDA	0.9	2x4	0.3	1	44	176	0.93
FDA	0.9	2x4	0.3	1	50	200	0.949
FDA	0.9	2x4	0.3	1	54	216	0.965
FDA	0.9	2x4	0.3	1	60	240	0.982
FDA	0.9	2x4	0.3	1	66	264	0.992
FDA	0.9	2x4	0.3	1	72	288	0.993
FDA	0.9	2x4	0.3	1	80	320	0.996
FDA	1	2x2	0.3	1	12	24	0.071
FDA	1	2x2	0.3	1	16	32	0.154
FDA	1	2x2	0.3	1	24	48	0.437
FDA	1	2x2	0.3	1	28	56	0.592
FDA	1	2x2	0.3	1	32	64	0.702
FDA	1	2x2	0.3	1	36	72	0.77
FDA	1	2x2	0.3	1	40	80	0.829
FDA	1	2x2	0.3	1	44	88	0.862
FDA	1	2x2	0.3	1	60	120	0.967
FDA	1	2x2	0.3	1	72	144	0.987
FDA	1	2x2	0.3	1	80	160	0.994
FDA	1	2x2	0.3	1	88	176	0.996
FDA	1	2x2	0.3	1	100	200	0.998

FDA	1	2x2	0.3	1	108	216	0.999
FDA	1	2x2	0.3	1	120	240	0.999
FDA	1	2x2	0.3	1	132	264	1
FDA	1	2x2	0.3	1	144	288	1
FDA	1	2x2	0.3	1	160	320	1
FDA	1	3x3	0.3	1	9	27	0.177
FDA	1	3x3	0.3	1	12	36	0.334
FDA	1	3x3	0.3	1	15	45	0.506
FDA	1	3x3	0.3	1	18	54	0.617
FDA	1	3x3	0.3	1	21	63	0.732
FDA	1	3x3	0.3	1	24	72	0.791
FDA	1	3x3	0.3	1	27	81	0.871
FDA	1	3x3	0.3	1	30	90	0.897
FDA	1	3x3	0.3	1	39	117	0.964
FDA	1	3x3	0.3	1	48	144	0.98
FDA	1	3x3	0.3	1	54	162	0.995
FDA	1	3x3	0.3	1	60	180	0.996
FDA	1	3x3	0.3	1	66	198	0.997
FDA	1	3x3	0.3	1	75	225	1
FDA	1	3x3	0.3	1	81	243	1
FDA	1	3x3	0.3	1	90	270	1
FDA	1	3x3	0.3	1	96	288	1
FDA	1	3x3	0.3	1	105	315	1
FDA	1	2x4	0.3	1	6	24	0.114
FDA	1	2x4	0.3	1	8	32	0.201
FDA	1	2x4	0.3	1	12	48	0.456
FDA	1	2x4	0.3	1	14	56	0.573
FDA	1	2x4	0.3	1	16	64	0.705
FDA	1	2x4	0.3	1	18	72	0.784
FDA	1	2x4	0.3	1	20	80	0.833
FDA	1	2x4	0.3	1	22	88	0.854
FDA	1	2x4	0.3	1	30	120	0.968
FDA	1	2x4	0.3	1	36	144	0.982
FDA	1	2x4	0.3	1	40	160	0.995
FDA	1	2x4	0.3	1	44	176	0.998
FDA	1	2x4	0.3	1	50	200	0.996
FDA	1	2x4	0.3	1	54	216	1
FDA	1	2x4	0.3	1	60	240	1
FDA	1	2x4	0.3	1	66	264	1
FDA	1	2x4	0.3	1	72	288	1
FDA	1	2x4	0.3	1	80	320	1

Table A4. Comparison of power among clinical trial designs by correlation coefficient of absorption rate co nstant (Ka) between reference and test drugs: The simulation results are summarized in Figure 5.

Agency	Correlation	Design	CV	GMR	Total number of subject	Total number of hospitalization (N)	Power
EMA	0.7	2x2	0.3	1	12	24	0.015
EMA	0.7	2x2	0.3	1	16	32	0.034
EMA	0.7	2x2	0.3	1	24	48	0.2
EMA	0.7	2x2	0.3	1	28	56	0.305
EMA	0.7	2x2	0.3	1	32	64	0.419
EMA	0.7	2x2	0.3	1	36	72	0.506
EMA	0.7	2x2	0.3	1	40	80	0.591
EMA	0.7	2x2	0.3	1	44	88	0.631
EMA	0.7	2x2	0.3	1	60	120	0.819
EMA	0.7	2x2	0.3	1	72	144	0.913
EMA	0.7	2x2	0.3	1	80	160	0.938
EMA	0.7	2x2	0.3	1	88	176	0.973
EMA	0.7	2x2	0.3	1	100	200	0.985
EMA	0.7	2x2	0.3	1	108	216	0.989
EMA	0.7	2x2	0.3	1	120	240	0.994
EMA	0.7	2x2	0.3	1	132	264	0.999
EMA	0.7	2x2	0.3	1	144	288	0.999
EMA	0.7	2x2	0.3	1	160	320	0.999
EMA	0.7	3x3	0.3	1	9	27	0.015
EMA	0.7	3x3	0.3	1	12	36	0.074
EMA	0.7	3x3	0.3	1	15	45	0.195
EMA	0.7	3x3	0.3	1	18	54	0.295
EMA	0.7	3x3	0.3	1	21	63	0.411
EMA	0.7	3x3	0.3	1	24	72	0.483
EMA	0.7	3x3	0.3	1	27	81	0.608
EMA	0.7	3x3	0.3	1	30	90	0.668
EMA	0.7	3x3	0.3	1	39	117	0.778
EMA	0.7	3x3	0.3	1	48	144	0.902
EMA	0.7	3x3	0.3	1	54	162	0.916
EMA	0.7	3x3	0.3	1	60	180	0.965
EMA	0.7	3x3	0.3	1	66	198	0.973
EMA	0.7	3x3	0.3	1	75	225	0.989
EMA	0.7	3x3	0.3	1	81	243	0.994
EMA	0.7	3x3	0.3	1	90	270	0.994
EMA	0.7	3x3	0.3	1	96	288	0.996
EMA	0.7	3x3	0.3	1	105	315	0.995
EMA	0.7	2x4	0.3	1	6	24	0.022

EMA	0.7	2x4	0.3	1	8	32	0.066
EMA	0.7	2x4	0.3	1	12	48	0.243
EMA	0.7	2x4	0.3	1	14	56	0.38
EMA	0.7	2x4	0.3	1	16	64	0.454
EMA	0.7	2x4	0.3	1	18	72	0.537
EMA	0.7	2x4	0.3	1	20	80	0.618
EMA	0.7	2x4	0.3	1	22	88	0.663
EMA	0.7	2x4	0.3	1	30	120	0.821
EMA	0.7	2x4	0.3	1	36	144	0.892
EMA	0.7	2x4	0.3	1	40	160	0.934
EMA	0.7	2x4	0.3	1	44	176	0.958
EMA	0.7	2x4	0.3	1	50	200	0.973
EMA	0.7	2x4	0.3	1	54	216	0.985
EMA	0.7	2x4	0.3	1	60	240	0.984
EMA	0.7	2x4	0.3	1	66	264	0.998
EMA	0.7	2x4	0.3	1	72	288	0.995
EMA	0.7	2x4	0.3	1	80	320	0.998
EMA	0.8	2x2	0.3	1	12	24	0.018
EMA	0.8	2x2	0.3	1	16	32	0.044
EMA	0.8	2x2	0.3	1	24	48	0.228
EMA	0.8	2x2	0.3	1	28	56	0.338
EMA	0.8	2x2	0.3	1	32	64	0.445
EMA	0.8	2x2	0.3	1	36	72	0.53
EMA	0.8	2x2	0.3	1	40	80	0.61
EMA	0.8	2x2	0.3	1	44	88	0.662
EMA	0.8	2x2	0.3	1	60	120	0.834
EMA	0.8	2x2	0.3	1	72	144	0.921
EMA	0.8	2x2	0.3	1	80	160	0.948
EMA	0.8	2x2	0.3	1	88	176	0.978
EMA	0.8	2x2	0.3	1	100	200	0.987
EMA	0.8	2x2	0.3	1	108	216	0.992
EMA	0.8	2x2	0.3	1	120	240	0.995
EMA	0.8	2x2	0.3	1	132	264	0.999
EMA	0.8	2x2	0.3	1	144	288	0.999
EMA	0.8	2x2	0.3	1	160	320	0.999
EMA	0.8	3x3	0.3	1	9	27	0.018
EMA	0.8	3x3	0.3	1	12	36	0.089
EMA	0.8	3x3	0.3	1	15	45	0.206
EMA	0.8	3x3	0.3	1	18	54	0.321
EMA	0.8	3x3	0.3	1	21	63	0.43
EMA	0.8	3x3	0.3	1	24	72	0.507

EMA	0.8	3x3	0.3	1	27	81	0.628
EMA	0.8	3x3	0.3	1	30	90	0.689
EMA	0.8	3x3	0.3	1	39	117	0.785
EMA	0.8	3x3	0.3	1	48	144	0.912
EMA	0.8	3x3	0.3	1	54	162	0.926
EMA	0.8	3x3	0.3	1	60	180	0.966
EMA	0.8	3x3	0.3	1	66	198	0.977
EMA	0.8	3x3	0.3	1	75	225	0.989
EMA	0.8	3x3	0.3	1	81	243	0.994
EMA	0.8	3x3	0.3	1	90	270	0.995
EMA	0.8	3x3	0.3	1	96	288	0.998
EMA	0.8	3x3	0.3	1	105	315	0.997
EMA	0.8	2x4	0.3	1	6	24	0.024
EMA	0.8	2x4	0.3	1	8	32	0.081
EMA	0.8	2x4	0.3	1	12	48	0.263
EMA	0.8	2x4	0.3	1	14	56	0.402
EMA	0.8	2x4	0.3	1	16	64	0.473
EMA	0.8	2x4	0.3	1	18	72	0.554
EMA	0.8	2x4	0.3	1	20	80	0.64
EMA	0.8	2x4	0.3	1	22	88	0.684
EMA	0.8	2x4	0.3	1	30	120	0.833
EMA	0.8	2x4	0.3	1	36	144	0.902
EMA	0.8	2x4	0.3	1	40	160	0.939
EMA	0.8	2x4	0.3	1	44	176	0.964
EMA	0.8	2x4	0.3	1	50	200	0.977
EMA	0.8	2x4	0.3	1	54	216	0.988
EMA	0.8	2x4	0.3	1	60	240	0.987
EMA	0.8	2x4	0.3	1	66	264	0.999
EMA	0.8	2x4	0.3	1	72	288	0.998
EMA	0.8	2x4	0.3	1	80	320	0.999
EMA	1	2x2	0.3	1	12	24	0.034
EMA	1	2x2	0.3	1	16	32	0.068
EMA	1	2x2	0.3	1	24	48	0.268
EMA	1	2x2	0.3	1	28	56	0.393
EMA	1	2x2	0.3	1	32	64	0.479
EMA	1	2x2	0.3	1	36	72	0.586
EMA	1	2x2	0.3	1	40	80	0.666
EMA	1	2x2	0.3	1	44	88	0.704
EMA	1	2x2	0.3	1	60	120	0.876
EMA	1	2x2	0.3	1	72	144	0.931
EMA	1	2x2	0.3	1	80	160	0.959

EMA	1	2x2	0.3	1	88	176	0.982
EMA	1	2x2	0.3	1	100	200	0.988
EMA	1	2x2	0.3	1	108	216	0.997
EMA	1	2x2	0.3	1	120	240	1
EMA	1	2x2	0.3	1	132	264	0.999
EMA	1	2x2	0.3	1	144	288	0.998
EMA	1	2x2	0.3	1	160	320	1
EMA	1	3x3	0.3	1	9	27	0.046
EMA	1	3x3	0.3	1	12	36	0.132
EMA	1	3x3	0.3	1	15	45	0.233
EMA	1	3x3	0.3	1	18	54	0.371
EMA	1	3x3	0.3	1	21	63	0.475
EMA	1	3x3	0.3	1	24	72	0.569
EMA	1	3x3	0.3	1	27	81	0.667
EMA	1	3x3	0.3	1	30	90	0.724
EMA	1	3x3	0.3	1	39	117	0.828
EMA	1	3x3	0.3	1	48	144	0.937
EMA	1	3x3	0.3	1	54	162	0.949
EMA	1	3x3	0.3	1	60	180	0.974
EMA	1	3x3	0.3	1	66	198	0.986
EMA	1	3x3	0.3	1	75	225	0.996
EMA	1	3x3	0.3	1	81	243	0.995
EMA	1	3x3	0.3	1	90	270	0.997
EMA	1	3x3	0.3	1	96	288	0.999
EMA	1	3x3	0.3	1	105	315	0.998
EMA	1	2x4	0.3	1	6	24	0.035
EMA	1	2x4	0.3	1	8	32	0.11
EMA	1	2x4	0.3	1	12	48	0.305
EMA	1	2x4	0.3	1	14	56	0.446
EMA	1	2x4	0.3	1	16	64	0.522
EMA	1	2x4	0.3	1	18	72	0.619
EMA	1	2x4	0.3	1	20	80	0.663
EMA	1	2x4	0.3	1	22	88	0.73
EMA	1	2x4	0.3	1	30	120	0.858
EMA	1	2x4	0.3	1	36	144	0.91
EMA	1	2x4	0.3	1	40	160	0.952
EMA	1	2x4	0.3	1	44	176	0.974
EMA	1	2x4	0.3	1	50	200	0.983
EMA	1	2x4	0.3	1	54	216	0.992
EMA	1	2x4	0.3	1	60	240	0.995
EMA	1	2x4	0.3	1	66	264	1

EMA	1	2x4	0.3	1	72	288	1
EMA	1	2x4	0.3	1	80	320	0.999
FDA	0.7	2x2	0.3	1	12	24	0.015
FDA	0.7	2x2	0.3	1	16	32	0.034
FDA	0.7	2x2	0.3	1	24	48	0.2
FDA	0.7	2x2	0.3	1	28	56	0.305
FDA	0.7	2x2	0.3	1	32	64	0.419
FDA	0.7	2x2	0.3	1	36	72	0.506
FDA	0.7	2x2	0.3	1	40	80	0.591
FDA	0.7	2x2	0.3	1	44	88	0.631
FDA	0.7	2x2	0.3	1	60	120	0.819
FDA	0.7	2x2	0.3	1	72	144	0.913
FDA	0.7	2x2	0.3	1	80	160	0.938
FDA	0.7	2x2	0.3	1	88	176	0.973
FDA	0.7	2x2	0.3	1	100	200	0.985
FDA	0.7	2x2	0.3	1	108	216	0.989
FDA	0.7	2x2	0.3	1	120	240	0.994
FDA	0.7	2x2	0.3	1	132	264	0.999
FDA	0.7	2x2	0.3	1	144	288	0.999
FDA	0.7	2x2	0.3	1	160	320	0.999
FDA	0.7	3x3	0.3	1	9	27	0.065
FDA	0.7	3x3	0.3	1	12	36	0.126
FDA	0.7	3x3	0.3	1	15	45	0.256
FDA	0.7	3x3	0.3	1	18	54	0.362
FDA	0.7	3x3	0.3	1	21	63	0.469
FDA	0.7	3x3	0.3	1	24	72	0.555
FDA	0.7	3x3	0.3	1	27	81	0.662
FDA	0.7	3x3	0.3	1	30	90	0.73
FDA	0.7	3x3	0.3	1	39	117	0.82
FDA	0.7	3x3	0.3	1	48	144	0.927
FDA	0.7	3x3	0.3	1	54	162	0.936
FDA	0.7	3x3	0.3	1	60	180	0.974
FDA	0.7	3x3	0.3	1	66	198	0.984
FDA	0.7	3x3	0.3	1	75	225	0.992
FDA	0.7	3x3	0.3	1	81	243	0.995
FDA	0.7	3x3	0.3	1	90	270	0.995
FDA	0.7	3x3	0.3	1	96	288	0.997
FDA	0.7	3x3	0.3	1	105	315	0.997
FDA	0.7	2x4	0.3	1	6	24	0.033
FDA	0.7	2x4	0.3	1	8	32	0.056
FDA	0.7	2x4	0.3	1	12	48	0.131

FDA	0.7	2x4	0.3	1	14	56	0.214
FDA	0.7	2x4	0.3	1	16	64	0.289
FDA	0.7	2x4	0.3	1	18	72	0.365
FDA	0.7	2x4	0.3	1	20	80	0.433
FDA	0.7	2x4	0.3	1	22	88	0.528
FDA	0.7	2x4	0.3	1	30	120	0.735
FDA	0.7	2x4	0.3	1	36	144	0.841
FDA	0.7	2x4	0.3	1	40	160	0.876
FDA	0.7	2x4	0.3	1	44	176	0.922
FDA	0.7	2x4	0.3	1	50	200	0.936
FDA	0.7	2x4	0.3	1	54	216	0.96
FDA	0.7	2x4	0.3	1	60	240	0.977
FDA	0.7	2x4	0.3	1	66	264	0.991
FDA	0.7	2x4	0.3	1	72	288	0.992
FDA	0.7	2x4	0.3	1	80	320	0.993
FDA	0.8	2x2	0.3	1	12	24	0.018
FDA	0.8	2x2	0.3	1	16	32	0.044
FDA	0.8	2x2	0.3	1	24	48	0.228
FDA	0.8	2x2	0.3	1	28	56	0.338
FDA	0.8	2x2	0.3	1	32	64	0.445
FDA	0.8	2x2	0.3	1	36	72	0.53
FDA	0.8	2x2	0.3	1	40	80	0.61
FDA	0.8	2x2	0.3	1	44	88	0.662
FDA	0.8	2x2	0.3	1	60	120	0.834
FDA	0.8	2x2	0.3	1	72	144	0.921
FDA	0.8	2x2	0.3	1	80	160	0.948
FDA	0.8	2x2	0.3	1	88	176	0.978
FDA	0.8	2x2	0.3	1	100	200	0.987
FDA	0.8	2x2	0.3	1	108	216	0.992
FDA	0.8	2x2	0.3	1	120	240	0.995
FDA	0.8	2x2	0.3	1	132	264	0.999
FDA	0.8	2x2	0.3	1	144	288	0.999
FDA	0.8	2x2	0.3	1	160	320	0.999
FDA	0.8	3x3	0.3	1	9	27	0.07
FDA	0.8	3x3	0.3	1	12	36	0.145
FDA	0.8	3x3	0.3	1	15	45	0.276
FDA	0.8	3x3	0.3	1	18	54	0.389
FDA	0.8	3x3	0.3	1	21	63	0.494
FDA	0.8	3x3	0.3	1	24	72	0.579
FDA	0.8	3x3	0.3	1	27	81	0.677
FDA	0.8	3x3	0.3	1	30	90	0.749

FDA	0.8	3x3	0.3	1	39	117	0.832
FDA	0.8	3x3	0.3	1	48	144	0.937
FDA	0.8	3x3	0.3	1	54	162	0.943
FDA	0.8	3x3	0.3	1	60	180	0.977
FDA	0.8	3x3	0.3	1	66	198	0.985
FDA	0.8	3x3	0.3	1	75	225	0.992
FDA	0.8	3x3	0.3	1	81	243	0.995
FDA	0.8	3x3	0.3	1	90	270	0.995
FDA	0.8	3x3	0.3	1	96	288	0.999
FDA	0.8	3x3	0.3	1	105	315	0.998
FDA	0.8	2x4	0.3	1	6	24	0.041
FDA	0.8	2x4	0.3	1	8	32	0.058
FDA	0.8	2x4	0.3	1	12	48	0.15
FDA	0.8	2x4	0.3	1	14	56	0.234
FDA	0.8	2x4	0.3	1	16	64	0.308
FDA	0.8	2x4	0.3	1	18	72	0.399
FDA	0.8	2x4	0.3	1	20	80	0.471
FDA	0.8	2x4	0.3	1	22	88	0.557
FDA	0.8	2x4	0.3	1	30	120	0.764
FDA	0.8	2x4	0.3	1	36	144	0.855
FDA	0.8	2x4	0.3	1	40	160	0.89
FDA	0.8	2x4	0.3	1	44	176	0.93
FDA	0.8	2x4	0.3	1	50	200	0.949
FDA	0.8	2x4	0.3	1	54	216	0.965
FDA	0.8	2x4	0.3	1	60	240	0.982
FDA	0.8	2x4	0.3	1	66	264	0.992
FDA	0.8	2x4	0.3	1	72	288	0.993
FDA	0.8	2x4	0.3	1	80	320	0.996
FDA	1	2x2	0.3	1	12	24	0.034
FDA	1	2x2	0.3	1	16	32	0.068
FDA	1	2x2	0.3	1	24	48	0.268
FDA	1	2x2	0.3	1	28	56	0.393
FDA	1	2x2	0.3	1	32	64	0.479
FDA	1	2x2	0.3	1	36	72	0.586
FDA	1	2x2	0.3	1	40	80	0.666
FDA	1	2x2	0.3	1	44	88	0.704
FDA	1	2x2	0.3	1	60	120	0.876
FDA	1	2x2	0.3	1	72	144	0.931
FDA	1	2x2	0.3	1	80	160	0.959
FDA	1	2x2	0.3	1	88	176	0.982
FDA	1	2x2	0.3	1	100	200	0.988

FDA	1	2x2	0.3	1	108	216	0.997
FDA	1	2x2	0.3	1	120	240	1
FDA	1	2x2	0.3	1	132	264	0.999
FDA	1	2x2	0.3	1	144	288	0.998
FDA	1	2x2	0.3	1	160	320	1
FDA	1	3x3	0.3	1	9	27	0.1
FDA	1	3x3	0.3	1	12	36	0.186
FDA	1	3x3	0.3	1	15	45	0.327
FDA	1	3x3	0.3	1	18	54	0.441
FDA	1	3x3	0.3	1	21	63	0.54
FDA	1	3x3	0.3	1	24	72	0.63
FDA	1	3x3	0.3	1	27	81	0.733
FDA	1	3x3	0.3	1	30	90	0.784
FDA	1	3x3	0.3	1	39	117	0.878
FDA	1	3x3	0.3	1	48	144	0.954
FDA	1	3x3	0.3	1	54	162	0.96
FDA	1	3x3	0.3	1	60	180	0.984
FDA	1	3x3	0.3	1	66	198	0.989
FDA	1	3x3	0.3	1	75	225	0.997
FDA	1	3x3	0.3	1	81	243	0.997
FDA	1	3x3	0.3	1	90	270	0.999
FDA	1	3x3	0.3	1	96	288	0.999
FDA	1	3x3	0.3	1	105	315	0.999
FDA	1	2x4	0.3	1	6	24	0.04
FDA	1	2x4	0.3	1	8	32	0.082
FDA	1	2x4	0.3	1	12	48	0.204
FDA	1	2x4	0.3	1	14	56	0.297
FDA	1	2x4	0.3	1	16	64	0.388
FDA	1	2x4	0.3	1	18	72	0.475
FDA	1	2x4	0.3	1	20	80	0.557
FDA	1	2x4	0.3	1	22	88	0.635
FDA	1	2x4	0.3	1	30	120	0.812
FDA	1	2x4	0.3	1	36	144	0.895
FDA	1	2x4	0.3	1	40	160	0.926
FDA	1	2x4	0.3	1	44	176	0.945
FDA	1	2x4	0.3	1	50	200	0.97
FDA	1	2x4	0.3	1	54	216	0.979
FDA	1	2x4	0.3	1	60	240	0.987
FDA	1	2x4	0.3	1	66	264	0.995
FDA	1	2x4	0.3	1	72	288	0.997
FDA	1	2x4	0.3	1	80	320	0.998

Table A5. Comparison of power among clinical trial designs by typical value of Ka: The simulation results are summarized in Figure 5.

Agency	ka	Design	CV	GMR	Total number of subject	Total number of hospitalization (N)	Power
EMA	1.34	2x2	0.3	1	12	24	0.018
EMA	1.34	2x2	0.3	1	16	32	0.044
EMA	1.34	2x2	0.3	1	24	48	0.228
EMA	1.34	2x2	0.3	1	28	56	0.338
EMA	1.34	2x2	0.3	1	32	64	0.445
EMA	1.34	2x2	0.3	1	36	72	0.53
EMA	1.34	2x2	0.3	1	40	80	0.61
EMA	1.34	2x2	0.3	1	44	88	0.662
EMA	1.34	2x2	0.3	1	60	120	0.834
EMA	1.34	2x2	0.3	1	72	144	0.921
EMA	1.34	2x2	0.3	1	80	160	0.948
EMA	1.34	2x2	0.3	1	88	176	0.978
EMA	1.34	2x2	0.3	1	100	200	0.987
EMA	1.34	2x2	0.3	1	108	216	0.992
EMA	1.34	2x2	0.3	1	120	240	0.995
EMA	1.34	2x2	0.3	1	132	264	0.999
EMA	1.34	2x2	0.3	1	144	288	0.999
EMA	1.34	2x2	0.3	1	160	320	0.999
EMA	1.34	3x3	0.3	1	9	27	0.018
EMA	1.34	3x3	0.3	1	12	36	0.089
EMA	1.34	3x3	0.3	1	15	45	0.206
EMA	1.34	3x3	0.3	1	18	54	0.321
EMA	1.34	3x3	0.3	1	21	63	0.43
EMA	1.34	3x3	0.3	1	24	72	0.507
EMA	1.34	3x3	0.3	1	27	81	0.628
EMA	1.34	3x3	0.3	1	30	90	0.689
EMA	1.34	3x3	0.3	1	39	117	0.785
EMA	1.34	3x3	0.3	1	48	144	0.912
EMA	1.34	3x3	0.3	1	54	162	0.926
EMA	1.34	3x3	0.3	1	60	180	0.966
EMA	1.34	3x3	0.3	1	66	198	0.977
EMA	1.34	3x3	0.3	1	75	225	0.989
EMA	1.34	3x3	0.3	1	81	243	0.994
EMA	1.34	3x3	0.3	1	90	270	0.995
EMA	1.34	3x3	0.3	1	96	288	0.998
EMA	1.34	3x3	0.3	1	105	315	0.997
EMA	1.34	2x4	0.3	1	6	24	0.024

EMA	1.34	2x4	0.3	1	8	32	0.081
EMA	1.34	2x4	0.3	1	12	48	0.263
EMA	1.34	2x4	0.3	1	14	56	0.402
EMA	1.34	2x4	0.3	1	16	64	0.473
EMA	1.34	2x4	0.3	1	18	72	0.554
EMA	1.34	2x4	0.3	1	20	80	0.64
EMA	1.34	2x4	0.3	1	22	88	0.684
EMA	1.34	2x4	0.3	1	30	120	0.833
EMA	1.34	2x4	0.3	1	36	144	0.902
EMA	1.34	2x4	0.3	1	40	160	0.939
EMA	1.34	2x4	0.3	1	44	176	0.964
EMA	1.34	2x4	0.3	1	50	200	0.977
EMA	1.34	2x4	0.3	1	54	216	0.988
EMA	1.34	2x4	0.3	1	60	240	0.987
EMA	1.34	2x4	0.3	1	66	264	0.999
EMA	1.34	2x4	0.3	1	72	288	0.998
EMA	1.34	2x4	0.3	1	80	320	0.999
EMA	1.38	2x2	0.3	1	12	24	0.023
EMA	1.38	2x2	0.3	1	16	32	0.044
EMA	1.38	2x2	0.3	1	24	48	0.206
EMA	1.38	2x2	0.3	1	28	56	0.32
EMA	1.38	2x2	0.3	1	32	64	0.419
EMA	1.38	2x2	0.3	1	36	72	0.5
EMA	1.38	2x2	0.3	1	40	80	0.587
EMA	1.38	2x2	0.3	1	44	88	0.623
EMA	1.38	2x2	0.3	1	60	120	0.816
EMA	1.38	2x2	0.3	1	72	144	0.905
EMA	1.38	2x2	0.3	1	80	160	0.937
EMA	1.38	2x2	0.3	1	88	176	0.967
EMA	1.38	2x2	0.3	1	100	200	0.98
EMA	1.38	2x2	0.3	1	108	216	0.984
EMA	1.38	2x2	0.3	1	120	240	0.991
EMA	1.38	2x2	0.3	1	132	264	0.997
EMA	1.38	2x2	0.3	1	144	288	0.996
EMA	1.38	2x2	0.3	1	160	320	0.998
EMA	1.38	3x3	0.3	1	9	27	0.018
EMA	1.38	3x3	0.3	1	12	36	0.084
EMA	1.38	3x3	0.3	1	15	45	0.2
EMA	1.38	3x3	0.3	1	18	54	0.293
EMA	1.38	3x3	0.3	1	21	63	0.418
EMA	1.38	3x3	0.3	1	24	72	0.48

EMA	1.38	3x3	0.3	1	27	81	0.604
EMA	1.38	3x3	0.3	1	30	90	0.661
EMA	1.38	3x3	0.3	1	39	117	0.772
EMA	1.38	3x3	0.3	1	48	144	0.897
EMA	1.38	3x3	0.3	1	54	162	0.904
EMA	1.38	3x3	0.3	1	60	180	0.953
EMA	1.38	3x3	0.3	1	66	198	0.966
EMA	1.38	3x3	0.3	1	75	225	0.982
EMA	1.38	3x3	0.3	1	81	243	0.989
EMA	1.38	3x3	0.3	1	90	270	0.989
EMA	1.38	3x3	0.3	1	96	288	0.992
EMA	1.38	3x3	0.3	1	105	315	0.991
EMA	1.38	2x4	0.3	1	6	24	0.023
EMA	1.38	2x4	0.3	1	8	32	0.078
EMA	1.38	2x4	0.3	1	12	48	0.247
EMA	1.38	2x4	0.3	1	14	56	0.394
EMA	1.38	2x4	0.3	1	16	64	0.456
EMA	1.38	2x4	0.3	1	18	72	0.546
EMA	1.38	2x4	0.3	1	20	80	0.613
EMA	1.38	2x4	0.3	1	22	88	0.663
EMA	1.38	2x4	0.3	1	30	120	0.823
EMA	1.38	2x4	0.3	1	36	144	0.892
EMA	1.38	2x4	0.3	1	40	160	0.929
EMA	1.38	2x4	0.3	1	44	176	0.951
EMA	1.38	2x4	0.3	1	50	200	0.967
EMA	1.38	2x4	0.3	1	54	216	0.98
EMA	1.38	2x4	0.3	1	60	240	0.981
EMA	1.38	2x4	0.3	1	66	264	0.996
EMA	1.38	2x4	0.3	1	72	288	0.995
EMA	1.38	2x4	0.3	1	80	320	0.997
FDA	1.34	2x2	0.3	1	12	24	0.018
FDA	1.34	2x2	0.3	1	16	32	0.044
FDA	1.34	2x2	0.3	1	24	48	0.228
FDA	1.34	2x2	0.3	1	28	56	0.338
FDA	1.34	2x2	0.3	1	32	64	0.445
FDA	1.34	2x2	0.3	1	36	72	0.53
FDA	1.34	2x2	0.3	1	40	80	0.61
FDA	1.34	2x2	0.3	1	44	88	0.662
FDA	1.34	2x2	0.3	1	60	120	0.834
FDA	1.34	2x2	0.3	1	72	144	0.921
FDA	1.34	2x2	0.3	1	80	160	0.948

FDA	1.34	2x2	0.3	1	88	176	0.978
FDA	1.34	2x2	0.3	1	100	200	0.987
FDA	1.34	2x2	0.3	1	108	216	0.992
FDA	1.34	2x2	0.3	1	120	240	0.995
FDA	1.34	2x2	0.3	1	132	264	0.999
FDA	1.34	2x2	0.3	1	144	288	0.999
FDA	1.34	2x2	0.3	1	160	320	0.999
FDA	1.34	3x3	0.3	1	9	27	0.07
FDA	1.34	3x3	0.3	1	12	36	0.145
FDA	1.34	3x3	0.3	1	15	45	0.276
FDA	1.34	3x3	0.3	1	18	54	0.389
FDA	1.34	3x3	0.3	1	21	63	0.494
FDA	1.34	3x3	0.3	1	24	72	0.579
FDA	1.34	3x3	0.3	1	27	81	0.677
FDA	1.34	3x3	0.3	1	30	90	0.749
FDA	1.34	3x3	0.3	1	39	117	0.832
FDA	1.34	3x3	0.3	1	48	144	0.937
FDA	1.34	3x3	0.3	1	54	162	0.943
FDA	1.34	3x3	0.3	1	60	180	0.977
FDA	1.34	3x3	0.3	1	66	198	0.985
FDA	1.34	3x3	0.3	1	75	225	0.992
FDA	1.34	3x3	0.3	1	81	243	0.995
FDA	1.34	3x3	0.3	1	90	270	0.995
FDA	1.34	3x3	0.3	1	96	288	0.999
FDA	1.34	3x3	0.3	1	105	315	0.998
FDA	1.34	2x4	0.3	1	6	24	0.041
FDA	1.34	2x4	0.3	1	8	32	0.058
FDA	1.34	2x4	0.3	1	12	48	0.15
FDA	1.34	2x4	0.3	1	14	56	0.234
FDA	1.34	2x4	0.3	1	16	64	0.308
FDA	1.34	2x4	0.3	1	18	72	0.399
FDA	1.34	2x4	0.3	1	20	80	0.471
FDA	1.34	2x4	0.3	1	22	88	0.557
FDA	1.34	2x4	0.3	1	30	120	0.764
FDA	1.34	2x4	0.3	1	36	144	0.855
FDA	1.34	2x4	0.3	1	40	160	0.89
FDA	1.34	2x4	0.3	1	44	176	0.93
FDA	1.34	2x4	0.3	1	50	200	0.949
FDA	1.34	2x4	0.3	1	54	216	0.965
FDA	1.34	2x4	0.3	1	60	240	0.982
FDA	1.34	2x4	0.3	1	66	264	0.992

FDA	1.34	2x4	0.3	1	72	288	0.993
FDA	1.34	2x4	0.3	1	80	320	0.996
FDA	1.38	2x2	0.3	1	12	24	0.023
FDA	1.38	2x2	0.3	1	16	32	0.044
FDA	1.38	2x2	0.3	1	24	48	0.206
FDA	1.38	2x2	0.3	1	28	56	0.32
FDA	1.38	2x2	0.3	1	32	64	0.419
FDA	1.38	2x2	0.3	1	36	72	0.5
FDA	1.38	2x2	0.3	1	40	80	0.587
FDA	1.38	2x2	0.3	1	44	88	0.623
FDA	1.38	2x2	0.3	1	60	120	0.816
FDA	1.38	2x2	0.3	1	72	144	0.905
FDA	1.38	2x2	0.3	1	80	160	0.937
FDA	1.38	2x2	0.3	1	88	176	0.967
FDA	1.38	2x2	0.3	1	100	200	0.98
FDA	1.38	2x2	0.3	1	108	216	0.984
FDA	1.38	2x2	0.3	1	120	240	0.991
FDA	1.38	2x2	0.3	1	132	264	0.997
FDA	1.38	2x2	0.3	1	144	288	0.996
FDA	1.38	2x2	0.3	1	160	320	0.998
FDA	1.38	3x3	0.3	1	9	27	0.073
FDA	1.38	3x3	0.3	1	12	36	0.142
FDA	1.38	3x3	0.3	1	15	45	0.27
FDA	1.38	3x3	0.3	1	18	54	0.365
FDA	1.38	3x3	0.3	1	21	63	0.483
FDA	1.38	3x3	0.3	1	24	72	0.545
FDA	1.38	3x3	0.3	1	27	81	0.663
FDA	1.38	3x3	0.3	1	30	90	0.723
FDA	1.38	3x3	0.3	1	39	117	0.813
FDA	1.38	3x3	0.3	1	48	144	0.921
FDA	1.38	3x3	0.3	1	54	162	0.929
FDA	1.38	3x3	0.3	1	60	180	0.962
FDA	1.38	3x3	0.3	1	66	198	0.977
FDA	1.38	3x3	0.3	1	75	225	0.989
FDA	1.38	3x3	0.3	1	81	243	0.992
FDA	1.38	3x3	0.3	1	90	270	0.993
FDA	1.38	3x3	0.3	1	96	288	0.996
FDA	1.38	3x3	0.3	1	105	315	0.993
FDA	1.38	2x4	0.3	1	6	24	0.039
FDA	1.38	2x4	0.3	1	8	32	0.058
FDA	1.38	2x4	0.3	1	12	48	0.137

FDA	1.38	2x4	0.3	1	14	56	0.24
FDA	1.38	2x4	0.3	1	16	64	0.301
FDA	1.38	2x4	0.3	1	18	72	0.394
FDA	1.38	2x4	0.3	1	20	80	0.455
FDA	1.38	2x4	0.3	1	22	88	0.532
FDA	1.38	2x4	0.3	1	30	120	0.74
FDA	1.38	2x4	0.3	1	36	144	0.831
FDA	1.38	2x4	0.3	1	40	160	0.872
FDA	1.38	2x4	0.3	1	44	176	0.914
FDA	1.38	2x4	0.3	1	50	200	0.937
FDA	1.38	2x4	0.3	1	54	216	0.956
FDA	1.38	2x4	0.3	1	60	240	0.972
FDA	1.38	2x4	0.3	1	66	264	0.985
FDA	1.38	2x4	0.3	1	72	288	0.992
FDA	1.38	2x4	0.3	1	80	320	0.994
