# Supplementary material for: Model-Based Approach for Designing an Efficient Bioequivalence Study for Highly Variable Drugs
Source: Pharmaceuticals (Basel). 2021 Oct 28;14(11):1101. doi: 10.3390/ph14111101 (PMC8624447; doi:10.3390/ph14111101)
Supplement: Supplementary file 1 [file pharmaceuticals-14-01101-s001.zip › [Supplementary File S4] Dropout and Error.pdf]

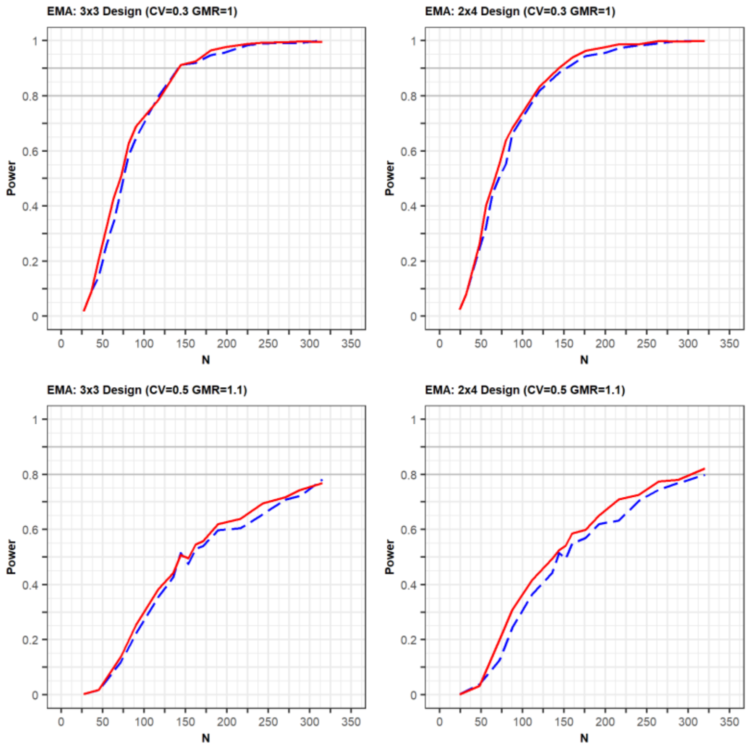


**Figure S1.** Comparison of power between simulations with dropout (blue long-dashed line) and without dropouts (red solid line): The power curves as per the EMA guideline are represented.

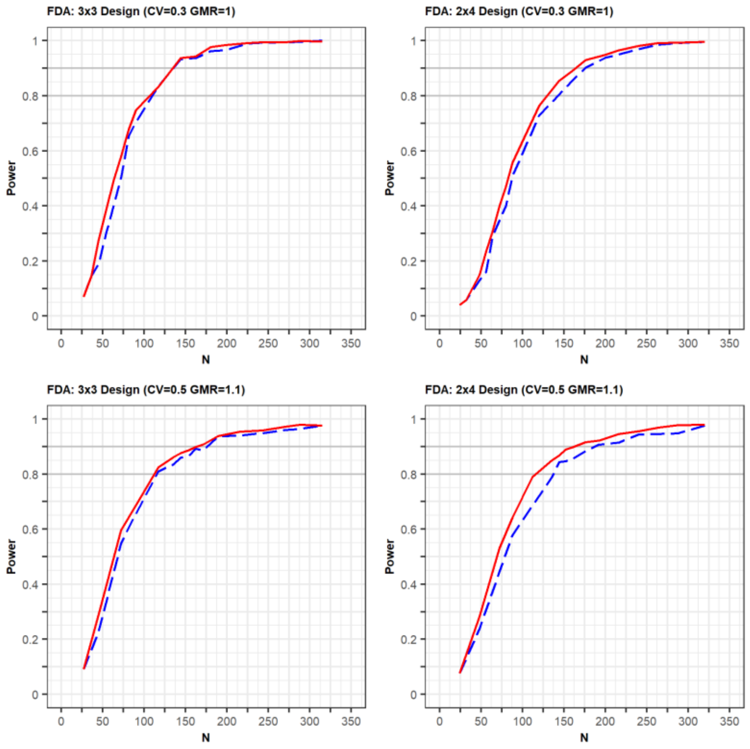


**Figure S2.** Comparison of power between simulations with dropout (blue long-dashed line) and without dropouts (red solid line): The power curves as per the FDA guideline are represented.

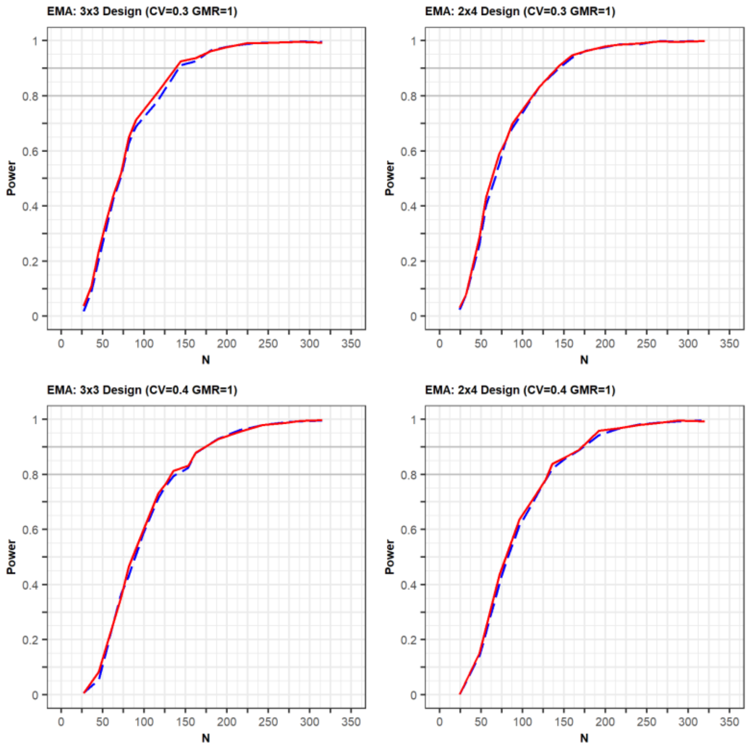


**Figure S3.** Comparison of power between simulations with errors (blue long-dashed line) and without errors (red solid line): The power curves as per the EMA guideline are represented.

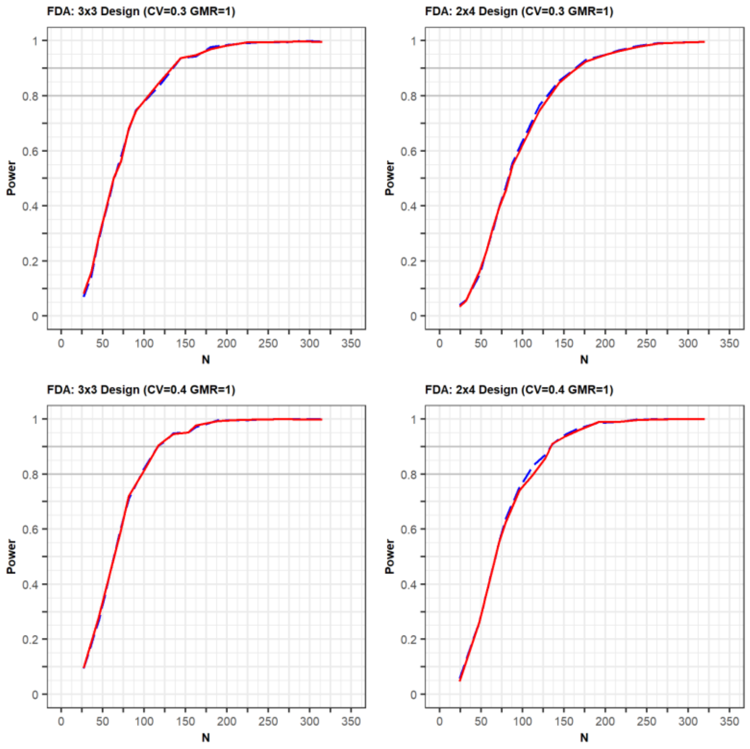


**Figure S4.** Comparison of power between simulations with errors (blue long-dashed line) and without errors (red solid line): The power curves as per the FDA guideline are represented.

**Table S6.** Comparison of power between simulations with and without dropouts: The simulation results are summarized in Figure S1 and Figure S2.

| Agency | Dropout | Design | CV | GMR | Total number of subject | Total number of hospitalization (N) | Power |
|---|---|---|---|---|---|---|---|
| EMA | No | 2x2 | 0.3 | 1 | 12 | 24 | 0.018 |
| EMA | No | 2x2 | 0.3 | 1 | 16 | 32 | 0.044 |
| EMA | No | 2x2 | 0.3 | 1 | 24 | 48 | 0.228 |
| EMA | No | 2x2 | 0.3 | 1 | 28 | 56 | 0.338 |
| EMA | No | 2x2 | 0.3 | 1 | 32 | 64 | 0.445 |
| EMA | No | 2x2 | 0.3 | 1 | 36 | 72 | 0.53 |
| EMA | No | 2x2 | 0.3 | 1 | 40 | 80 | 0.61 |
| EMA | No | 2x2 | 0.3 | 1 | 44 | 88 | 0.662 |
| EMA | No | 2x2 | 0.3 | 1 | 60 | 120 | 0.834 |
| EMA | No | 2x2 | 0.3 | 1 | 72 | 144 | 0.921 |
| EMA | No | 2x2 | 0.3 | 1 | 80 | 160 | 0.948 |
| EMA | No | 2x2 | 0.3 | 1 | 88 | 176 | 0.978 |
| EMA | No | 2x2 | 0.3 | 1 | 100 | 200 | 0.987 |
| EMA | No | 2x2 | 0.3 | 1 | 108 | 216 | 0.992 |
| EMA | No | 2x2 | 0.3 | 1 | 120 | 240 | 0.995 |
| EMA | No | 2x2 | 0.3 | 1 | 132 | 264 | 0.999 |
| EMA | No | 2x2 | 0.3 | 1 | 144 | 288 | 0.999 |
| EMA | No | 2x2 | 0.3 | 1 | 160 | 320 | 0.999 |
| EMA | No | 2x2 | 0.5 | 1.1 | 12 | 24 | 0.002 |
| EMA | No | 2x2 | 0.5 | 1.1 | 24 | 48 | 0.008 |
| EMA | No | 2x2 | 0.5 | 1.1 | 36 | 72 | 0.11 |
| EMA | No | 2x2 | 0.5 | 1.1 | 44 | 88 | 0.202 |
| EMA | No | 2x2 | 0.5 | 1.1 | 56 | 112 | 0.298 |
| EMA | No | 2x2 | 0.5 | 1.1 | 68 | 136 | 0.376 |
| EMA | No | 2x2 | 0.5 | 1.1 | 72 | 144 | 0.414 |
| EMA | No | 2x2 | 0.5 | 1.1 | 76 | 152 | 0.437 |
| EMA | No | 2x2 | 0.5 | 1.1 | 80 | 160 | 0.45 |
| EMA | No | 2x2 | 0.5 | 1.1 | 88 | 176 | 0.505 |
| EMA | No | 2x2 | 0.5 | 1.1 | 96 | 192 | 0.525 |
| EMA | No | 2x2 | 0.5 | 1.1 | 108 | 216 | 0.57 |
| EMA | No | 2x2 | 0.5 | 1.1 | 120 | 240 | 0.598 |
| EMA | No | 2x2 | 0.5 | 1.1 | 132 | 264 | 0.653 |
| EMA | No | 2x2 | 0.5 | 1.1 | 144 | 288 | 0.718 |
| EMA | No | 2x2 | 0.5 | 1.1 | 160 | 320 | 0.739 |
| EMA | No | 3x3 | 0.3 | 1 | 9 | 27 | 0.018 |
| EMA | No | 3x3 | 0.3 | 1 | 12 | 36 | 0.089 |
| EMA | No | 3x3 | 0.3 | 1 | 15 | 45 | 0.206 |

| | | | | | | | |
|---|---|---|---|---|---|---|---|
| EMA | No | 3x3 | 0.3 | 1 | 18 | 54 | 0.321 |
| EMA | No | 3x3 | 0.3 | 1 | 21 | 63 | 0.43 |
| EMA | No | 3x3 | 0.3 | 1 | 24 | 72 | 0.507 |
| EMA | No | 3x3 | 0.3 | 1 | 27 | 81 | 0.628 |
| EMA | No | 3x3 | 0.3 | 1 | 30 | 90 | 0.689 |
| EMA | No | 3x3 | 0.3 | 1 | 39 | 117 | 0.785 |
| EMA | No | 3x3 | 0.3 | 1 | 48 | 144 | 0.912 |
| EMA | No | 3x3 | 0.3 | 1 | 54 | 162 | 0.926 |
| EMA | No | 3x3 | 0.3 | 1 | 60 | 180 | 0.966 |
| EMA | No | 3x3 | 0.3 | 1 | 66 | 198 | 0.977 |
| EMA | No | 3x3 | 0.3 | 1 | 75 | 225 | 0.989 |
| EMA | No | 3x3 | 0.3 | 1 | 81 | 243 | 0.994 |
| EMA | No | 3x3 | 0.3 | 1 | 90 | 270 | 0.995 |
| EMA | No | 3x3 | 0.3 | 1 | 96 | 288 | 0.998 |
| EMA | No | 3x3 | 0.3 | 1 | 105 | 315 | 0.997 |
| EMA | No | 3x3 | 0.5 | 1.1 | 9 | 27 | 0.003 |
| EMA | No | 3x3 | 0.5 | 1.1 | 15 | 45 | 0.017 |
| EMA | No | 3x3 | 0.5 | 1.1 | 24 | 72 | 0.139 |
| EMA | No | 3x3 | 0.5 | 1.1 | 30 | 90 | 0.253 |
| EMA | No | 3x3 | 0.5 | 1.1 | 39 | 117 | 0.384 |
| EMA | No | 3x3 | 0.5 | 1.1 | 45 | 135 | 0.443 |
| EMA | No | 3x3 | 0.5 | 1.1 | 48 | 144 | 0.508 |
| EMA | No | 3x3 | 0.5 | 1.1 | 51 | 153 | 0.495 |
| EMA | No | 3x3 | 0.5 | 1.1 | 54 | 162 | 0.545 |
| EMA | No | 3x3 | 0.5 | 1.1 | 57 | 171 | 0.558 |
| EMA | No | 3x3 | 0.5 | 1.1 | 63 | 189 | 0.619 |
| EMA | No | 3x3 | 0.5 | 1.1 | 72 | 216 | 0.639 |
| EMA | No | 3x3 | 0.5 | 1.1 | 81 | 243 | 0.694 |
| EMA | No | 3x3 | 0.5 | 1.1 | 90 | 270 | 0.717 |
| EMA | No | 3x3 | 0.5 | 1.1 | 96 | 288 | 0.744 |
| EMA | No | 3x3 | 0.5 | 1.1 | 105 | 315 | 0.768 |
| EMA | No | 2x4 | 0.3 | 1 | 6 | 24 | 0.024 |
| EMA | No | 2x4 | 0.3 | 1 | 8 | 32 | 0.081 |
| EMA | No | 2x4 | 0.3 | 1 | 12 | 48 | 0.263 |
| EMA | No | 2x4 | 0.3 | 1 | 14 | 56 | 0.402 |
| EMA | No | 2x4 | 0.3 | 1 | 16 | 64 | 0.473 |
| EMA | No | 2x4 | 0.3 | 1 | 18 | 72 | 0.554 |
| EMA | No | 2x4 | 0.3 | 1 | 20 | 80 | 0.64 |
| EMA | No | 2x4 | 0.3 | 1 | 22 | 88 | 0.684 |
| EMA | No | 2x4 | 0.3 | 1 | 30 | 120 | 0.833 |
| EMA | No | 2x4 | 0.3 | 1 | 36 | 144 | 0.902 |

| | | | | | | | |
|---|---|---|---|---|---|---|---|
| EMA | No | 2x4 | 0.3 | 1 | 40 | 160 | 0.939 |
| EMA | No | 2x4 | 0.3 | 1 | 44 | 176 | 0.964 |
| EMA | No | 2x4 | 0.3 | 1 | 50 | 200 | 0.977 |
| EMA | No | 2x4 | 0.3 | 1 | 54 | 216 | 0.988 |
| EMA | No | 2x4 | 0.3 | 1 | 60 | 240 | 0.987 |
| EMA | No | 2x4 | 0.3 | 1 | 66 | 264 | 0.999 |
| EMA | No | 2x4 | 0.3 | 1 | 72 | 288 | 0.998 |
| EMA | No | 2x4 | 0.3 | 1 | 80 | 320 | 0.999 |
| EMA | No | 2x4 | 0.5 | 1.1 | 6 | 24 | 0.001 |
| EMA | No | 2x4 | 0.5 | 1.1 | 12 | 48 | 0.033 |
| EMA | No | 2x4 | 0.5 | 1.1 | 18 | 72 | 0.199 |
| EMA | No | 2x4 | 0.5 | 1.1 | 22 | 88 | 0.309 |
| EMA | No | 2x4 | 0.5 | 1.1 | 28 | 112 | 0.418 |
| EMA | No | 2x4 | 0.5 | 1.1 | 34 | 136 | 0.494 |
| EMA | No | 2x4 | 0.5 | 1.1 | 36 | 144 | 0.525 |
| EMA | No | 2x4 | 0.5 | 1.1 | 38 | 152 | 0.541 |
| EMA | No | 2x4 | 0.5 | 1.1 | 40 | 160 | 0.585 |
| EMA | No | 2x4 | 0.5 | 1.1 | 44 | 176 | 0.599 |
| EMA | No | 2x4 | 0.5 | 1.1 | 48 | 192 | 0.65 |
| EMA | No | 2x4 | 0.5 | 1.1 | 54 | 216 | 0.71 |
| EMA | No | 2x4 | 0.5 | 1.1 | 60 | 240 | 0.726 |
| EMA | No | 2x4 | 0.5 | 1.1 | 66 | 264 | 0.774 |
| EMA | No | 2x4 | 0.5 | 1.1 | 72 | 288 | 0.78 |
| EMA | No | 2x4 | 0.5 | 1.1 | 80 | 320 | 0.822 |
| EMA | Yes | 2x2 | 0.3 | 1 | 12 | 24 | 0.018 |
| EMA | Yes | 2x2 | 0.3 | 1 | 16 | 32 | 0.044 |
| EMA | Yes | 2x2 | 0.3 | 1 | 24 | 48 | 0.162 |
| EMA | Yes | 2x2 | 0.3 | 1 | 28 | 56 | 0.272 |
| EMA | Yes | 2x2 | 0.3 | 1 | 32 | 64 | 0.44 |
| EMA | Yes | 2x2 | 0.3 | 1 | 36 | 72 | 0.467 |
| EMA | Yes | 2x2 | 0.3 | 1 | 40 | 80 | 0.565 |
| EMA | Yes | 2x2 | 0.3 | 1 | 44 | 88 | 0.65 |
| EMA | Yes | 2x2 | 0.3 | 1 | 60 | 120 | 0.844 |
| EMA | Yes | 2x2 | 0.3 | 1 | 72 | 144 | 0.902 |
| EMA | Yes | 2x2 | 0.3 | 1 | 80 | 160 | 0.947 |
| EMA | Yes | 2x2 | 0.3 | 1 | 88 | 176 | 0.967 |
| EMA | Yes | 2x2 | 0.3 | 1 | 100 | 200 | 0.978 |
| EMA | Yes | 2x2 | 0.3 | 1 | 108 | 216 | 0.978 |
| EMA | Yes | 2x2 | 0.3 | 1 | 120 | 240 | 0.996 |
| EMA | Yes | 2x2 | 0.3 | 1 | 132 | 264 | 0.998 |
| EMA | Yes | 2x2 | 0.3 | 1 | 144 | 288 | 0.999 |

| | | | | | | | |
|---|---|---|---|---|---|---|---|
| EMA | Yes | 2x2 | 0.3 | 1 | 160 | 320 | 0.996 |
| EMA | Yes | 2x2 | 0.5 | 1.1 | 12 | 24 | 0.002 |
| EMA | Yes | 2x2 | 0.5 | 1.1 | 24 | 48 | 0.014 |
| EMA | Yes | 2x2 | 0.5 | 1.1 | 36 | 72 | 0.076 |
| EMA | Yes | 2x2 | 0.5 | 1.1 | 44 | 88 | 0.18 |
| EMA | Yes | 2x2 | 0.5 | 1.1 | 56 | 112 | 0.279 |
| EMA | Yes | 2x2 | 0.5 | 1.1 | 68 | 136 | 0.347 |
| EMA | Yes | 2x2 | 0.5 | 1.1 | 72 | 144 | 0.407 |
| EMA | Yes | 2x2 | 0.5 | 1.1 | 76 | 152 | 0.414 |
| EMA | Yes | 2x2 | 0.5 | 1.1 | 80 | 160 | 0.44 |
| EMA | Yes | 2x2 | 0.5 | 1.1 | 88 | 176 | 0.463 |
| EMA | Yes | 2x2 | 0.5 | 1.1 | 96 | 192 | 0.482 |
| EMA | Yes | 2x2 | 0.5 | 1.1 | 108 | 216 | 0.57 |
| EMA | Yes | 2x2 | 0.5 | 1.1 | 120 | 240 | 0.6 |
| EMA | Yes | 2x2 | 0.5 | 1.1 | 132 | 264 | 0.659 |
| EMA | Yes | 2x2 | 0.5 | 1.1 | 144 | 288 | 0.694 |
| EMA | Yes | 2x2 | 0.5 | 1.1 | 160 | 320 | 0.712 |
| EMA | Yes | 3x3 | 0.3 | 1 | 9 | 27 | 0.018 |
| EMA | Yes | 3x3 | 0.3 | 1 | 12 | 36 | 0.089 |
| EMA | Yes | 3x3 | 0.3 | 1 | 15 | 45 | 0.144 |
| EMA | Yes | 3x3 | 0.3 | 1 | 18 | 54 | 0.257 |
| EMA | Yes | 3x3 | 0.3 | 1 | 21 | 63 | 0.341 |
| EMA | Yes | 3x3 | 0.3 | 1 | 24 | 72 | 0.459 |
| EMA | Yes | 3x3 | 0.3 | 1 | 27 | 81 | 0.584 |
| EMA | Yes | 3x3 | 0.3 | 1 | 30 | 90 | 0.646 |
| EMA | Yes | 3x3 | 0.3 | 1 | 39 | 117 | 0.799 |
| EMA | Yes | 3x3 | 0.3 | 1 | 48 | 144 | 0.912 |
| EMA | Yes | 3x3 | 0.3 | 1 | 54 | 162 | 0.919 |
| EMA | Yes | 3x3 | 0.3 | 1 | 60 | 180 | 0.948 |
| EMA | Yes | 3x3 | 0.3 | 1 | 66 | 198 | 0.958 |
| EMA | Yes | 3x3 | 0.3 | 1 | 75 | 225 | 0.985 |
| EMA | Yes | 3x3 | 0.3 | 1 | 81 | 243 | 0.99 |
| EMA | Yes | 3x3 | 0.3 | 1 | 90 | 270 | 0.992 |
| EMA | Yes | 3x3 | 0.3 | 1 | 96 | 288 | 0.992 |
| EMA | Yes | 3x3 | 0.3 | 1 | 105 | 315 | 1 |
| EMA | Yes | 3x3 | 0.5 | 1.1 | 9 | 27 | 0.003 |
| EMA | Yes | 3x3 | 0.5 | 1.1 | 15 | 45 | 0.016 |
| EMA | Yes | 3x3 | 0.5 | 1.1 | 24 | 72 | 0.12 |
| EMA | Yes | 3x3 | 0.5 | 1.1 | 30 | 90 | 0.222 |
| EMA | Yes | 3x3 | 0.5 | 1.1 | 39 | 117 | 0.356 |
| EMA | Yes | 3x3 | 0.5 | 1.1 | 45 | 135 | 0.428 |

| | | | | | | | |
|---|---|---|---|---|---|---|---|
| EMA | Yes | 3x3 | 0.5 | 1.1 | 48 | 144 | 0.514 |
| EMA | Yes | 3x3 | 0.5 | 1.1 | 51 | 153 | 0.475 |
| EMA | Yes | 3x3 | 0.5 | 1.1 | 54 | 162 | 0.53 |
| EMA | Yes | 3x3 | 0.5 | 1.1 | 57 | 171 | 0.54 |
| EMA | Yes | 3x3 | 0.5 | 1.1 | 63 | 189 | 0.597 |
| EMA | Yes | 3x3 | 0.5 | 1.1 | 72 | 216 | 0.604 |
| EMA | Yes | 3x3 | 0.5 | 1.1 | 81 | 243 | 0.655 |
| EMA | Yes | 3x3 | 0.5 | 1.1 | 90 | 270 | 0.708 |
| EMA | Yes | 3x3 | 0.5 | 1.1 | 96 | 288 | 0.721 |
| EMA | Yes | 3x3 | 0.5 | 1.1 | 105 | 315 | 0.781 |
| EMA | Yes | 2x4 | 0.3 | 1 | 6 | 24 | 0.024 |
| EMA | Yes | 2x4 | 0.3 | 1 | 8 | 32 | 0.081 |
| EMA | Yes | 2x4 | 0.3 | 1 | 12 | 48 | 0.247 |
| EMA | Yes | 2x4 | 0.3 | 1 | 14 | 56 | 0.324 |
| EMA | Yes | 2x4 | 0.3 | 1 | 16 | 64 | 0.444 |
| EMA | Yes | 2x4 | 0.3 | 1 | 18 | 72 | 0.506 |
| EMA | Yes | 2x4 | 0.3 | 1 | 20 | 80 | 0.552 |
| EMA | Yes | 2x4 | 0.3 | 1 | 22 | 88 | 0.664 |
| EMA | Yes | 2x4 | 0.3 | 1 | 30 | 120 | 0.818 |
| EMA | Yes | 2x4 | 0.3 | 1 | 36 | 144 | 0.884 |
| EMA | Yes | 2x4 | 0.3 | 1 | 40 | 160 | 0.913 |
| EMA | Yes | 2x4 | 0.3 | 1 | 44 | 176 | 0.944 |
| EMA | Yes | 2x4 | 0.3 | 1 | 50 | 200 | 0.956 |
| EMA | Yes | 2x4 | 0.3 | 1 | 54 | 216 | 0.973 |
| EMA | Yes | 2x4 | 0.3 | 1 | 60 | 240 | 0.983 |
| EMA | Yes | 2x4 | 0.3 | 1 | 66 | 264 | 0.99 |
| EMA | Yes | 2x4 | 0.3 | 1 | 72 | 288 | 0.999 |
| EMA | Yes | 2x4 | 0.3 | 1 | 80 | 320 | 0.999 |
| EMA | Yes | 2x4 | 0.5 | 1.1 | 6 | 24 | 0.001 |
| EMA | Yes | 2x4 | 0.5 | 1.1 | 12 | 48 | 0.038 |
| EMA | Yes | 2x4 | 0.5 | 1.1 | 18 | 72 | 0.126 |
| EMA | Yes | 2x4 | 0.5 | 1.1 | 22 | 88 | 0.245 |
| EMA | Yes | 2x4 | 0.5 | 1.1 | 28 | 112 | 0.366 |
| EMA | Yes | 2x4 | 0.5 | 1.1 | 34 | 136 | 0.442 |
| EMA | Yes | 2x4 | 0.5 | 1.1 | 36 | 144 | 0.514 |
| EMA | Yes | 2x4 | 0.5 | 1.1 | 38 | 152 | 0.494 |
| EMA | Yes | 2x4 | 0.5 | 1.1 | 40 | 160 | 0.548 |
| EMA | Yes | 2x4 | 0.5 | 1.1 | 44 | 176 | 0.569 |
| EMA | Yes | 2x4 | 0.5 | 1.1 | 48 | 192 | 0.619 |
| EMA | Yes | 2x4 | 0.5 | 1.1 | 54 | 216 | 0.633 |
| EMA | Yes | 2x4 | 0.5 | 1.1 | 60 | 240 | 0.704 |

| | | | | | | | |
|---|---|---|---|---|---|---|---|
| EMA | Yes | 2x4 | 0.5 | 1.1 | 66 | 264 | 0.745 |
| EMA | Yes | 2x4 | 0.5 | 1.1 | 72 | 288 | 0.768 |
| EMA | Yes | 2x4 | 0.5 | 1.1 | 80 | 320 | 0.798 |
| FDA | No | 2x2 | 0.3 | 1 | 12 | 24 | 0.018 |
| FDA | No | 2x2 | 0.3 | 1 | 16 | 32 | 0.044 |
| FDA | No | 2x2 | 0.3 | 1 | 24 | 48 | 0.228 |
| FDA | No | 2x2 | 0.3 | 1 | 28 | 56 | 0.338 |
| FDA | No | 2x2 | 0.3 | 1 | 32 | 64 | 0.445 |
| FDA | No | 2x2 | 0.3 | 1 | 36 | 72 | 0.53 |
| FDA | No | 2x2 | 0.3 | 1 | 40 | 80 | 0.61 |
| FDA | No | 2x2 | 0.3 | 1 | 44 | 88 | 0.662 |
| FDA | No | 2x2 | 0.3 | 1 | 60 | 120 | 0.834 |
| FDA | No | 2x2 | 0.3 | 1 | 72 | 144 | 0.921 |
| FDA | No | 2x2 | 0.3 | 1 | 80 | 160 | 0.948 |
| FDA | No | 2x2 | 0.3 | 1 | 88 | 176 | 0.978 |
| FDA | No | 2x2 | 0.3 | 1 | 100 | 200 | 0.987 |
| FDA | No | 2x2 | 0.3 | 1 | 108 | 216 | 0.992 |
| FDA | No | 2x2 | 0.3 | 1 | 120 | 240 | 0.995 |
| FDA | No | 2x2 | 0.3 | 1 | 132 | 264 | 0.999 |
| FDA | No | 2x2 | 0.3 | 1 | 144 | 288 | 0.999 |
| FDA | No | 2x2 | 0.3 | 1 | 160 | 320 | 0.999 |
| FDA | No | 2x2 | 0.5 | 1.1 | 12 | 24 | 0.002 |
| FDA | No | 2x2 | 0.5 | 1.1 | 24 | 48 | 0.008 |
| FDA | No | 2x2 | 0.5 | 1.1 | 36 | 72 | 0.11 |
| FDA | No | 2x2 | 0.5 | 1.1 | 44 | 88 | 0.202 |
| FDA | No | 2x2 | 0.5 | 1.1 | 56 | 112 | 0.298 |
| FDA | No | 2x2 | 0.5 | 1.1 | 68 | 136 | 0.376 |
| FDA | No | 2x2 | 0.5 | 1.1 | 72 | 144 | 0.414 |
| FDA | No | 2x2 | 0.5 | 1.1 | 76 | 152 | 0.437 |
| FDA | No | 2x2 | 0.5 | 1.1 | 80 | 160 | 0.45 |
| FDA | No | 2x2 | 0.5 | 1.1 | 88 | 176 | 0.505 |
| FDA | No | 2x2 | 0.5 | 1.1 | 96 | 192 | 0.525 |
| FDA | No | 2x2 | 0.5 | 1.1 | 108 | 216 | 0.57 |
| FDA | No | 2x2 | 0.5 | 1.1 | 120 | 240 | 0.598 |
| FDA | No | 2x2 | 0.5 | 1.1 | 132 | 264 | 0.653 |
| FDA | No | 2x2 | 0.5 | 1.1 | 144 | 288 | 0.718 |
| FDA | No | 2x2 | 0.5 | 1.1 | 160 | 320 | 0.739 |
| FDA | No | 3x3 | 0.3 | 1 | 9 | 27 | 0.07 |
| FDA | No | 3x3 | 0.3 | 1 | 12 | 36 | 0.145 |
| FDA | No | 3x3 | 0.3 | 1 | 15 | 45 | 0.276 |
| FDA | No | 3x3 | 0.3 | 1 | 18 | 54 | 0.389 |

| | | | | | | | |
|---|---|---|---|---|---|---|---|
| FDA | No | 3x3 | 0.3 | 1 | 21 | 63 | 0.494 |
| FDA | No | 3x3 | 0.3 | 1 | 24 | 72 | 0.579 |
| FDA | No | 3x3 | 0.3 | 1 | 27 | 81 | 0.677 |
| FDA | No | 3x3 | 0.3 | 1 | 30 | 90 | 0.749 |
| FDA | No | 3x3 | 0.3 | 1 | 39 | 117 | 0.832 |
| FDA | No | 3x3 | 0.3 | 1 | 48 | 144 | 0.937 |
| FDA | No | 3x3 | 0.3 | 1 | 54 | 162 | 0.943 |
| FDA | No | 3x3 | 0.3 | 1 | 60 | 180 | 0.977 |
| FDA | No | 3x3 | 0.3 | 1 | 66 | 198 | 0.985 |
| FDA | No | 3x3 | 0.3 | 1 | 75 | 225 | 0.992 |
| FDA | No | 3x3 | 0.3 | 1 | 81 | 243 | 0.995 |
| FDA | No | 3x3 | 0.3 | 1 | 90 | 270 | 0.995 |
| FDA | No | 3x3 | 0.3 | 1 | 96 | 288 | 0.999 |
| FDA | No | 3x3 | 0.3 | 1 | 105 | 315 | 0.998 |
| FDA | No | 3x3 | 0.5 | 1.1 | 9 | 27 | 0.091 |
| FDA | No | 3x3 | 0.5 | 1.1 | 15 | 45 | 0.291 |
| FDA | No | 3x3 | 0.5 | 1.1 | 24 | 72 | 0.596 |
| FDA | No | 3x3 | 0.5 | 1.1 | 30 | 90 | 0.687 |
| FDA | No | 3x3 | 0.5 | 1.1 | 39 | 117 | 0.824 |
| FDA | No | 3x3 | 0.5 | 1.1 | 45 | 135 | 0.861 |
| FDA | No | 3x3 | 0.5 | 1.1 | 48 | 144 | 0.876 |
| FDA | No | 3x3 | 0.5 | 1.1 | 51 | 153 | 0.886 |
| FDA | No | 3x3 | 0.5 | 1.1 | 54 | 162 | 0.898 |
| FDA | No | 3x3 | 0.5 | 1.1 | 57 | 171 | 0.907 |
| FDA | No | 3x3 | 0.5 | 1.1 | 63 | 189 | 0.938 |
| FDA | No | 3x3 | 0.5 | 1.1 | 72 | 216 | 0.954 |
| FDA | No | 3x3 | 0.5 | 1.1 | 81 | 243 | 0.958 |
| FDA | No | 3x3 | 0.5 | 1.1 | 90 | 270 | 0.972 |
| FDA | No | 3x3 | 0.5 | 1.1 | 96 | 288 | 0.979 |
| FDA | No | 3x3 | 0.5 | 1.1 | 105 | 315 | 0.976 |
| FDA | No | 2x4 | 0.3 | 1 | 6 | 24 | 0.041 |
| FDA | No | 2x4 | 0.3 | 1 | 8 | 32 | 0.058 |
| FDA | No | 2x4 | 0.3 | 1 | 12 | 48 | 0.15 |
| FDA | No | 2x4 | 0.3 | 1 | 14 | 56 | 0.234 |
| FDA | No | 2x4 | 0.3 | 1 | 16 | 64 | 0.308 |
| FDA | No | 2x4 | 0.3 | 1 | 18 | 72 | 0.399 |
| FDA | No | 2x4 | 0.3 | 1 | 20 | 80 | 0.471 |
| FDA | No | 2x4 | 0.3 | 1 | 22 | 88 | 0.557 |
| FDA | No | 2x4 | 0.3 | 1 | 30 | 120 | 0.764 |
| FDA | No | 2x4 | 0.3 | 1 | 36 | 144 | 0.855 |
| FDA | No | 2x4 | 0.3 | 1 | 40 | 160 | 0.89 |

| | | | | | | | |
|---|---|---|---|---|---|---|---|
| FDA | No | 2x4 | 0.3 | 1 | 44 | 176 | 0.93 |
| FDA | No | 2x4 | 0.3 | 1 | 50 | 200 | 0.949 |
| FDA | No | 2x4 | 0.3 | 1 | 54 | 216 | 0.965 |
| FDA | No | 2x4 | 0.3 | 1 | 60 | 240 | 0.982 |
| FDA | No | 2x4 | 0.3 | 1 | 66 | 264 | 0.992 |
| FDA | No | 2x4 | 0.3 | 1 | 72 | 288 | 0.993 |
| FDA | No | 2x4 | 0.3 | 1 | 80 | 320 | 0.996 |
| FDA | No | 2x4 | 0.5 | 1.1 | 6 | 24 | 0.077 |
| FDA | No | 2x4 | 0.5 | 1.1 | 12 | 48 | 0.283 |
| FDA | No | 2x4 | 0.5 | 1.1 | 18 | 72 | 0.531 |
| FDA | No | 2x4 | 0.5 | 1.1 | 22 | 88 | 0.641 |
| FDA | No | 2x4 | 0.5 | 1.1 | 28 | 112 | 0.789 |
| FDA | No | 2x4 | 0.5 | 1.1 | 34 | 136 | 0.851 |
| FDA | No | 2x4 | 0.5 | 1.1 | 36 | 144 | 0.867 |
| FDA | No | 2x4 | 0.5 | 1.1 | 38 | 152 | 0.889 |
| FDA | No | 2x4 | 0.5 | 1.1 | 40 | 160 | 0.896 |
| FDA | No | 2x4 | 0.5 | 1.1 | 44 | 176 | 0.916 |
| FDA | No | 2x4 | 0.5 | 1.1 | 48 | 192 | 0.922 |
| FDA | No | 2x4 | 0.5 | 1.1 | 54 | 216 | 0.946 |
| FDA | No | 2x4 | 0.5 | 1.1 | 60 | 240 | 0.956 |
| FDA | No | 2x4 | 0.5 | 1.1 | 66 | 264 | 0.969 |
| FDA | No | 2x4 | 0.5 | 1.1 | 72 | 288 | 0.978 |
| FDA | No | 2x4 | 0.5 | 1.1 | 80 | 320 | 0.979 |
| FDA | Yes | 2x2 | 0.3 | 1 | 12 | 24 | 0.018 |
| FDA | Yes | 2x2 | 0.3 | 1 | 16 | 32 | 0.044 |
| FDA | Yes | 2x2 | 0.3 | 1 | 24 | 48 | 0.162 |
| FDA | Yes | 2x2 | 0.3 | 1 | 28 | 56 | 0.272 |
| FDA | Yes | 2x2 | 0.3 | 1 | 32 | 64 | 0.44 |
| FDA | Yes | 2x2 | 0.3 | 1 | 36 | 72 | 0.467 |
| FDA | Yes | 2x2 | 0.3 | 1 | 40 | 80 | 0.565 |
| FDA | Yes | 2x2 | 0.3 | 1 | 44 | 88 | 0.65 |
| FDA | Yes | 2x2 | 0.3 | 1 | 60 | 120 | 0.844 |
| FDA | Yes | 2x2 | 0.3 | 1 | 72 | 144 | 0.902 |
| FDA | Yes | 2x2 | 0.3 | 1 | 80 | 160 | 0.947 |
| FDA | Yes | 2x2 | 0.3 | 1 | 88 | 176 | 0.967 |
| FDA | Yes | 2x2 | 0.3 | 1 | 100 | 200 | 0.978 |
| FDA | Yes | 2x2 | 0.3 | 1 | 108 | 216 | 0.978 |
| FDA | Yes | 2x2 | 0.3 | 1 | 120 | 240 | 0.996 |
| FDA | Yes | 2x2 | 0.3 | 1 | 132 | 264 | 0.998 |
| FDA | Yes | 2x2 | 0.3 | 1 | 144 | 288 | 0.999 |
| FDA | Yes | 2x2 | 0.3 | 1 | 160 | 320 | 0.996 |

| | | | | | | | |
|---|---|---|---|---|---|---|---|
| FDA | Yes | 2x2 | 0.5 | 1.1 | 12 | 24 | 0.002 |
| FDA | Yes | 2x2 | 0.5 | 1.1 | 24 | 48 | 0.014 |
| FDA | Yes | 2x2 | 0.5 | 1.1 | 36 | 72 | 0.076 |
| FDA | Yes | 2x2 | 0.5 | 1.1 | 44 | 88 | 0.18 |
| FDA | Yes | 2x2 | 0.5 | 1.1 | 56 | 112 | 0.279 |
| FDA | Yes | 2x2 | 0.5 | 1.1 | 68 | 136 | 0.347 |
| FDA | Yes | 2x2 | 0.5 | 1.1 | 72 | 144 | 0.407 |
| FDA | Yes | 2x2 | 0.5 | 1.1 | 76 | 152 | 0.414 |
| FDA | Yes | 2x2 | 0.5 | 1.1 | 80 | 160 | 0.44 |
| FDA | Yes | 2x2 | 0.5 | 1.1 | 88 | 176 | 0.463 |
| FDA | Yes | 2x2 | 0.5 | 1.1 | 96 | 192 | 0.482 |
| FDA | Yes | 2x2 | 0.5 | 1.1 | 108 | 216 | 0.57 |
| FDA | Yes | 2x2 | 0.5 | 1.1 | 120 | 240 | 0.6 |
| FDA | Yes | 2x2 | 0.5 | 1.1 | 132 | 264 | 0.659 |
| FDA | Yes | 2x2 | 0.5 | 1.1 | 144 | 288 | 0.694 |
| FDA | Yes | 2x2 | 0.5 | 1.1 | 160 | 320 | 0.712 |
| FDA | Yes | 3x3 | 0.3 | 1 | 9 | 27 | 0.07 |
| FDA | Yes | 3x3 | 0.3 | 1 | 12 | 36 | 0.145 |
| FDA | Yes | 3x3 | 0.3 | 1 | 15 | 45 | 0.19 |
| FDA | Yes | 3x3 | 0.3 | 1 | 18 | 54 | 0.304 |
| FDA | Yes | 3x3 | 0.3 | 1 | 21 | 63 | 0.403 |
| FDA | Yes | 3x3 | 0.3 | 1 | 24 | 72 | 0.5 |
| FDA | Yes | 3x3 | 0.3 | 1 | 27 | 81 | 0.652 |
| FDA | Yes | 3x3 | 0.3 | 1 | 30 | 90 | 0.705 |
| FDA | Yes | 3x3 | 0.3 | 1 | 39 | 117 | 0.834 |
| FDA | Yes | 3x3 | 0.3 | 1 | 48 | 144 | 0.933 |
| FDA | Yes | 3x3 | 0.3 | 1 | 54 | 162 | 0.938 |
| FDA | Yes | 3x3 | 0.3 | 1 | 60 | 180 | 0.962 |
| FDA | Yes | 3x3 | 0.3 | 1 | 66 | 198 | 0.965 |
| FDA | Yes | 3x3 | 0.3 | 1 | 75 | 225 | 0.99 |
| FDA | Yes | 3x3 | 0.3 | 1 | 81 | 243 | 0.993 |
| FDA | Yes | 3x3 | 0.3 | 1 | 90 | 270 | 0.995 |
| FDA | Yes | 3x3 | 0.3 | 1 | 96 | 288 | 0.996 |
| FDA | Yes | 3x3 | 0.3 | 1 | 105 | 315 | 1 |
| FDA | Yes | 3x3 | 0.5 | 1.1 | 9 | 27 | 0.091 |
| FDA | Yes | 3x3 | 0.5 | 1.1 | 15 | 45 | 0.229 |
| FDA | Yes | 3x3 | 0.5 | 1.1 | 24 | 72 | 0.547 |
| FDA | Yes | 3x3 | 0.5 | 1.1 | 30 | 90 | 0.656 |
| FDA | Yes | 3x3 | 0.5 | 1.1 | 39 | 117 | 0.809 |
| FDA | Yes | 3x3 | 0.5 | 1.1 | 45 | 135 | 0.835 |
| FDA | Yes | 3x3 | 0.5 | 1.1 | 48 | 144 | 0.86 |

| | | | | | | | |
|---|---|---|---|---|---|---|---|
| FDA | Yes | 3x3 | 0.5 | 1.1 | 51 | 153 | 0.865 |
| FDA | Yes | 3x3 | 0.5 | 1.1 | 54 | 162 | 0.892 |
| FDA | Yes | 3x3 | 0.5 | 1.1 | 57 | 171 | 0.885 |
| FDA | Yes | 3x3 | 0.5 | 1.1 | 63 | 189 | 0.937 |
| FDA | Yes | 3x3 | 0.5 | 1.1 | 72 | 216 | 0.941 |
| FDA | Yes | 3x3 | 0.5 | 1.1 | 81 | 243 | 0.948 |
| FDA | Yes | 3x3 | 0.5 | 1.1 | 90 | 270 | 0.96 |
| FDA | Yes | 3x3 | 0.5 | 1.1 | 96 | 288 | 0.964 |
| FDA | Yes | 3x3 | 0.5 | 1.1 | 105 | 315 | 0.977 |
| FDA | Yes | 2x4 | 0.3 | 1 | 6 | 24 | 0.041 |
| FDA | Yes | 2x4 | 0.3 | 1 | 8 | 32 | 0.058 |
| FDA | Yes | 2x4 | 0.3 | 1 | 12 | 48 | 0.131 |
| FDA | Yes | 2x4 | 0.3 | 1 | 14 | 56 | 0.16 |
| FDA | Yes | 2x4 | 0.3 | 1 | 16 | 64 | 0.288 |
| FDA | Yes | 2x4 | 0.3 | 1 | 18 | 72 | 0.347 |
| FDA | Yes | 2x4 | 0.3 | 1 | 20 | 80 | 0.4 |
| FDA | Yes | 2x4 | 0.3 | 1 | 22 | 88 | 0.508 |
| FDA | Yes | 2x4 | 0.3 | 1 | 30 | 120 | 0.73 |
| FDA | Yes | 2x4 | 0.3 | 1 | 36 | 144 | 0.804 |
| FDA | Yes | 2x4 | 0.3 | 1 | 40 | 160 | 0.855 |
| FDA | Yes | 2x4 | 0.3 | 1 | 44 | 176 | 0.902 |
| FDA | Yes | 2x4 | 0.3 | 1 | 50 | 200 | 0.939 |
| FDA | Yes | 2x4 | 0.3 | 1 | 54 | 216 | 0.951 |
| FDA | Yes | 2x4 | 0.3 | 1 | 60 | 240 | 0.97 |
| FDA | Yes | 2x4 | 0.3 | 1 | 66 | 264 | 0.986 |
| FDA | Yes | 2x4 | 0.3 | 1 | 72 | 288 | 0.992 |
| FDA | Yes | 2x4 | 0.3 | 1 | 80 | 320 | 0.997 |
| FDA | Yes | 2x4 | 0.5 | 1.1 | 6 | 24 | 0.077 |
| FDA | Yes | 2x4 | 0.5 | 1.1 | 12 | 48 | 0.239 |
| FDA | Yes | 2x4 | 0.5 | 1.1 | 18 | 72 | 0.445 |
| FDA | Yes | 2x4 | 0.5 | 1.1 | 22 | 88 | 0.578 |
| FDA | Yes | 2x4 | 0.5 | 1.1 | 28 | 112 | 0.686 |
| FDA | Yes | 2x4 | 0.5 | 1.1 | 34 | 136 | 0.791 |
| FDA | Yes | 2x4 | 0.5 | 1.1 | 36 | 144 | 0.844 |
| FDA | Yes | 2x4 | 0.5 | 1.1 | 38 | 152 | 0.847 |
| FDA | Yes | 2x4 | 0.5 | 1.1 | 40 | 160 | 0.854 |
| FDA | Yes | 2x4 | 0.5 | 1.1 | 44 | 176 | 0.884 |
| FDA | Yes | 2x4 | 0.5 | 1.1 | 48 | 192 | 0.907 |
| FDA | Yes | 2x4 | 0.5 | 1.1 | 54 | 216 | 0.915 |
| FDA | Yes | 2x4 | 0.5 | 1.1 | 60 | 240 | 0.944 |
| FDA | Yes | 2x4 | 0.5 | 1.1 | 66 | 264 | 0.945 |

| | | | | | | | |
|---|---|---|---|---|---|---|---|
| FDA | Yes | 2x4 | 0.5 | 1.1 | 72 | 288 | 0.949 |
| FDA | Yes | 2x4 | 0.5 | 1.1 | 80 | 320 | 0.976 |

**Table S7.** Comparison of power between simulations with and without errors: The simulation results are summarized in Figure S3 and Figure S4.

| Agency | Error | Design | CV | GMR | Total number of subject | Total number of hospitalization (N) | Power |
|---|---|---|---|---|---|---|---|
| EMA | Yes | 2x2 | 0.3 | 1 | 12 | 24 | 0.018 |
| EMA | Yes | 2x2 | 0.3 | 1 | 16 | 32 | 0.044 |
| EMA | Yes | 2x2 | 0.3 | 1 | 24 | 48 | 0.228 |
| EMA | Yes | 2x2 | 0.3 | 1 | 28 | 56 | 0.338 |
| EMA | Yes | 2x2 | 0.3 | 1 | 32 | 64 | 0.445 |
| EMA | Yes | 2x2 | 0.3 | 1 | 36 | 72 | 0.53 |
| EMA | Yes | 2x2 | 0.3 | 1 | 40 | 80 | 0.61 |
| EMA | Yes | 2x2 | 0.3 | 1 | 44 | 88 | 0.662 |
| EMA | Yes | 2x2 | 0.3 | 1 | 60 | 120 | 0.834 |
| EMA | Yes | 2x2 | 0.3 | 1 | 72 | 144 | 0.921 |
| EMA | Yes | 2x2 | 0.3 | 1 | 80 | 160 | 0.948 |
| EMA | Yes | 2x2 | 0.3 | 1 | 88 | 176 | 0.978 |
| EMA | Yes | 2x2 | 0.3 | 1 | 100 | 200 | 0.987 |
| EMA | Yes | 2x2 | 0.3 | 1 | 108 | 216 | 0.992 |
| EMA | Yes | 2x2 | 0.3 | 1 | 120 | 240 | 0.995 |
| EMA | Yes | 2x2 | 0.3 | 1 | 132 | 264 | 0.999 |
| EMA | Yes | 2x2 | 0.3 | 1 | 144 | 288 | 0.999 |
| EMA | Yes | 2x2 | 0.3 | 1 | 160 | 320 | 0.999 |
| EMA | Yes | 2x2 | 0.4 | 1 | 12 | 24 | 0.007 |
| EMA | Yes | 2x2 | 0.4 | 1 | 24 | 48 | 0.07 |
| EMA | Yes | 2x2 | 0.4 | 1 | 36 | 72 | 0.29 |
| EMA | Yes | 2x2 | 0.4 | 1 | 40 | 80 | 0.385 |
| EMA | Yes | 2x2 | 0.4 | 1 | 48 | 96 | 0.521 |
| EMA | Yes | 2x2 | 0.4 | 1 | 56 | 112 | 0.65 |
| EMA | Yes | 2x2 | 0.4 | 1 | 64 | 128 | 0.737 |
| EMA | Yes | 2x2 | 0.4 | 1 | 68 | 136 | 0.787 |
| EMA | Yes | 2x2 | 0.4 | 1 | 76 | 152 | 0.809 |
| EMA | Yes | 2x2 | 0.4 | 1 | 84 | 168 | 0.866 |
| EMA | Yes | 2x2 | 0.4 | 1 | 96 | 192 | 0.924 |
| EMA | Yes | 2x2 | 0.4 | 1 | 108 | 216 | 0.944 |
| EMA | Yes | 2x2 | 0.4 | 1 | 120 | 240 | 0.971 |
| EMA | Yes | 2x2 | 0.4 | 1 | 132 | 264 | 0.978 |
| EMA | Yes | 2x2 | 0.4 | 1 | 144 | 288 | 0.985 |
| EMA | Yes | 2x2 | 0.4 | 1 | 160 | 320 | 0.994 |
| EMA | Yes | 3x3 | 0.3 | 1 | 9 | 27 | 0.018 |
| EMA | Yes | 3x3 | 0.3 | 1 | 12 | 36 | 0.089 |
| EMA | Yes | 3x3 | 0.3 | 1 | 15 | 45 | 0.206 |

| | | | | | | | |
|---|---|---|---|---|---|---|---|
| EMA | Yes | 3x3 | 0.3 | 1 | 18 | 54 | 0.321 |
| EMA | Yes | 3x3 | 0.3 | 1 | 21 | 63 | 0.43 |
| EMA | Yes | 3x3 | 0.3 | 1 | 24 | 72 | 0.507 |
| EMA | Yes | 3x3 | 0.3 | 1 | 27 | 81 | 0.628 |
| EMA | Yes | 3x3 | 0.3 | 1 | 30 | 90 | 0.689 |
| EMA | Yes | 3x3 | 0.3 | 1 | 39 | 117 | 0.785 |
| EMA | Yes | 3x3 | 0.3 | 1 | 48 | 144 | 0.912 |
| EMA | Yes | 3x3 | 0.3 | 1 | 54 | 162 | 0.926 |
| EMA | Yes | 3x3 | 0.3 | 1 | 60 | 180 | 0.966 |
| EMA | Yes | 3x3 | 0.3 | 1 | 66 | 198 | 0.977 |
| EMA | Yes | 3x3 | 0.3 | 1 | 75 | 225 | 0.989 |
| EMA | Yes | 3x3 | 0.3 | 1 | 81 | 243 | 0.994 |
| EMA | Yes | 3x3 | 0.3 | 1 | 90 | 270 | 0.995 |
| EMA | Yes | 3x3 | 0.3 | 1 | 96 | 288 | 0.998 |
| EMA | Yes | 3x3 | 0.3 | 1 | 105 | 315 | 0.997 |
| EMA | Yes | 3x3 | 0.4 | 1 | 9 | 27 | 0.007 |
| EMA | Yes | 3x3 | 0.4 | 1 | 15 | 45 | 0.055 |
| EMA | Yes | 3x3 | 0.4 | 1 | 24 | 72 | 0.364 |
| EMA | Yes | 3x3 | 0.4 | 1 | 27 | 81 | 0.431 |
| EMA | Yes | 3x3 | 0.4 | 1 | 33 | 99 | 0.586 |
| EMA | Yes | 3x3 | 0.4 | 1 | 39 | 117 | 0.714 |
| EMA | Yes | 3x3 | 0.4 | 1 | 42 | 126 | 0.756 |
| EMA | Yes | 3x3 | 0.4 | 1 | 45 | 135 | 0.793 |
| EMA | Yes | 3x3 | 0.4 | 1 | 51 | 153 | 0.825 |
| EMA | Yes | 3x3 | 0.4 | 1 | 54 | 162 | 0.877 |
| EMA | Yes | 3x3 | 0.4 | 1 | 63 | 189 | 0.929 |
| EMA | Yes | 3x3 | 0.4 | 1 | 72 | 216 | 0.961 |
| EMA | Yes | 3x3 | 0.4 | 1 | 81 | 243 | 0.979 |
| EMA | Yes | 3x3 | 0.4 | 1 | 90 | 270 | 0.989 |
| EMA | Yes | 3x3 | 0.4 | 1 | 96 | 288 | 0.992 |
| EMA | Yes | 3x3 | 0.4 | 1 | 105 | 315 | 0.996 |
| EMA | Yes | 2x4 | 0.3 | 1 | 6 | 24 | 0.024 |
| EMA | Yes | 2x4 | 0.3 | 1 | 8 | 32 | 0.081 |
| EMA | Yes | 2x4 | 0.3 | 1 | 12 | 48 | 0.263 |
| EMA | Yes | 2x4 | 0.3 | 1 | 14 | 56 | 0.402 |
| EMA | Yes | 2x4 | 0.3 | 1 | 16 | 64 | 0.473 |
| EMA | Yes | 2x4 | 0.3 | 1 | 18 | 72 | 0.554 |
| EMA | Yes | 2x4 | 0.3 | 1 | 20 | 80 | 0.64 |
| EMA | Yes | 2x4 | 0.3 | 1 | 22 | 88 | 0.684 |
| EMA | Yes | 2x4 | 0.3 | 1 | 30 | 120 | 0.833 |
| EMA | Yes | 2x4 | 0.3 | 1 | 36 | 144 | 0.902 |

| | | | | | | | |
|---|---|---|---|---|---|---|---|
| EMA | Yes | 2x4 | 0.3 | 1 | 40 | 160 | 0.939 |
| EMA | Yes | 2x4 | 0.3 | 1 | 44 | 176 | 0.964 |
| EMA | Yes | 2x4 | 0.3 | 1 | 50 | 200 | 0.977 |
| EMA | Yes | 2x4 | 0.3 | 1 | 54 | 216 | 0.988 |
| EMA | Yes | 2x4 | 0.3 | 1 | 60 | 240 | 0.987 |
| EMA | Yes | 2x4 | 0.3 | 1 | 66 | 264 | 0.999 |
| EMA | Yes | 2x4 | 0.3 | 1 | 72 | 288 | 0.998 |
| EMA | Yes | 2x4 | 0.3 | 1 | 80 | 320 | 0.999 |
| EMA | Yes | 2x4 | 0.4 | 1 | 6 | 24 | 0.002 |
| EMA | Yes | 2x4 | 0.4 | 1 | 12 | 48 | 0.141 |
| EMA | Yes | 2x4 | 0.4 | 1 | 18 | 72 | 0.403 |
| EMA | Yes | 2x4 | 0.4 | 1 | 20 | 80 | 0.483 |
| EMA | Yes | 2x4 | 0.4 | 1 | 24 | 96 | 0.613 |
| EMA | Yes | 2x4 | 0.4 | 1 | 28 | 112 | 0.694 |
| EMA | Yes | 2x4 | 0.4 | 1 | 32 | 128 | 0.78 |
| EMA | Yes | 2x4 | 0.4 | 1 | 34 | 136 | 0.82 |
| EMA | Yes | 2x4 | 0.4 | 1 | 38 | 152 | 0.858 |
| EMA | Yes | 2x4 | 0.4 | 1 | 42 | 168 | 0.888 |
| EMA | Yes | 2x4 | 0.4 | 1 | 48 | 192 | 0.943 |
| EMA | Yes | 2x4 | 0.4 | 1 | 54 | 216 | 0.966 |
| EMA | Yes | 2x4 | 0.4 | 1 | 60 | 240 | 0.981 |
| EMA | Yes | 2x4 | 0.4 | 1 | 66 | 264 | 0.988 |
| EMA | Yes | 2x4 | 0.4 | 1 | 72 | 288 | 0.993 |
| EMA | Yes | 2x4 | 0.4 | 1 | 80 | 320 | 0.996 |
| EMA | No | 2x2 | 0.3 | 1 | 12 | 24 | 0.028 |
| EMA | No | 2x2 | 0.3 | 1 | 16 | 32 | 0.067 |
| EMA | No | 2x2 | 0.3 | 1 | 24 | 48 | 0.259 |
| EMA | No | 2x2 | 0.3 | 1 | 28 | 56 | 0.383 |
| EMA | No | 2x2 | 0.3 | 1 | 32 | 64 | 0.498 |
| EMA | No | 2x2 | 0.3 | 1 | 36 | 72 | 0.58 |
| EMA | No | 2x2 | 0.3 | 1 | 40 | 80 | 0.641 |
| EMA | No | 2x2 | 0.3 | 1 | 44 | 88 | 0.693 |
| EMA | No | 2x2 | 0.3 | 1 | 60 | 120 | 0.876 |
| EMA | No | 2x2 | 0.3 | 1 | 72 | 144 | 0.935 |
| EMA | No | 2x2 | 0.3 | 1 | 80 | 160 | 0.955 |
| EMA | No | 2x2 | 0.3 | 1 | 88 | 176 | 0.982 |
| EMA | No | 2x2 | 0.3 | 1 | 100 | 200 | 0.986 |
| EMA | No | 2x2 | 0.3 | 1 | 108 | 216 | 0.994 |
| EMA | No | 2x2 | 0.3 | 1 | 120 | 240 | 0.997 |
| EMA | No | 2x2 | 0.3 | 1 | 132 | 264 | 0.999 |
| EMA | No | 2x2 | 0.3 | 1 | 144 | 288 | 0.999 |

| | | | | | | | |
|---|---|---|---|---|---|---|---|
| EMA | No | 2x2 | 0.3 | 1 | 160 | 320 | 1 |
| EMA | No | 2x2 | 0.4 | 1 | 12 | 24 | 0.01 |
| EMA | No | 2x2 | 0.4 | 1 | 24 | 48 | 0.087 |
| EMA | No | 2x2 | 0.4 | 1 | 36 | 72 | 0.317 |
| EMA | No | 2x2 | 0.4 | 1 | 40 | 80 | 0.396 |
| EMA | No | 2x2 | 0.4 | 1 | 48 | 96 | 0.558 |
| EMA | No | 2x2 | 0.4 | 1 | 56 | 112 | 0.666 |
| EMA | No | 2x2 | 0.4 | 1 | 64 | 128 | 0.751 |
| EMA | No | 2x2 | 0.4 | 1 | 68 | 136 | 0.804 |
| EMA | No | 2x2 | 0.4 | 1 | 76 | 152 | 0.828 |
| EMA | No | 2x2 | 0.4 | 1 | 84 | 168 | 0.893 |
| EMA | No | 2x2 | 0.4 | 1 | 96 | 192 | 0.938 |
| EMA | No | 2x2 | 0.4 | 1 | 108 | 216 | 0.95 |
| EMA | No | 2x2 | 0.4 | 1 | 120 | 240 | 0.975 |
| EMA | No | 2x2 | 0.4 | 1 | 132 | 264 | 0.984 |
| EMA | No | 2x2 | 0.4 | 1 | 144 | 288 | 0.987 |
| EMA | No | 2x2 | 0.4 | 1 | 160 | 320 | 0.995 |
| EMA | No | 3x3 | 0.3 | 1 | 9 | 27 | 0.037 |
| EMA | No | 3x3 | 0.3 | 1 | 12 | 36 | 0.109 |
| EMA | No | 3x3 | 0.3 | 1 | 15 | 45 | 0.235 |
| EMA | No | 3x3 | 0.3 | 1 | 18 | 54 | 0.348 |
| EMA | No | 3x3 | 0.3 | 1 | 21 | 63 | 0.443 |
| EMA | No | 3x3 | 0.3 | 1 | 24 | 72 | 0.518 |
| EMA | No | 3x3 | 0.3 | 1 | 27 | 81 | 0.648 |
| EMA | No | 3x3 | 0.3 | 1 | 30 | 90 | 0.713 |
| EMA | No | 3x3 | 0.3 | 1 | 39 | 117 | 0.817 |
| EMA | No | 3x3 | 0.3 | 1 | 48 | 144 | 0.926 |
| EMA | No | 3x3 | 0.3 | 1 | 54 | 162 | 0.938 |
| EMA | No | 3x3 | 0.3 | 1 | 60 | 180 | 0.962 |
| EMA | No | 3x3 | 0.3 | 1 | 66 | 198 | 0.975 |
| EMA | No | 3x3 | 0.3 | 1 | 75 | 225 | 0.992 |
| EMA | No | 3x3 | 0.3 | 1 | 81 | 243 | 0.992 |
| EMA | No | 3x3 | 0.3 | 1 | 90 | 270 | 0.995 |
| EMA | No | 3x3 | 0.3 | 1 | 96 | 288 | 0.997 |
| EMA | No | 3x3 | 0.3 | 1 | 105 | 315 | 0.994 |
| EMA | No | 3x3 | 0.4 | 1 | 9 | 27 | 0.006 |
| EMA | No | 3x3 | 0.4 | 1 | 15 | 45 | 0.082 |
| EMA | No | 3x3 | 0.4 | 1 | 24 | 72 | 0.352 |
| EMA | No | 3x3 | 0.4 | 1 | 27 | 81 | 0.465 |
| EMA | No | 3x3 | 0.4 | 1 | 33 | 99 | 0.597 |
| EMA | No | 3x3 | 0.4 | 1 | 39 | 117 | 0.732 |

| | | | | | | | |
|---|---|---|---|---|---|---|---|
| EMA | No | 3x3 | 0.4 | 1 | 42 | 126 | 0.766 |
| EMA | No | 3x3 | 0.4 | 1 | 45 | 135 | 0.813 |
| EMA | No | 3x3 | 0.4 | 1 | 51 | 153 | 0.832 |
| EMA | No | 3x3 | 0.4 | 1 | 54 | 162 | 0.879 |
| EMA | No | 3x3 | 0.4 | 1 | 63 | 189 | 0.928 |
| EMA | No | 3x3 | 0.4 | 1 | 72 | 216 | 0.956 |
| EMA | No | 3x3 | 0.4 | 1 | 81 | 243 | 0.979 |
| EMA | No | 3x3 | 0.4 | 1 | 90 | 270 | 0.987 |
| EMA | No | 3x3 | 0.4 | 1 | 96 | 288 | 0.994 |
| EMA | No | 3x3 | 0.4 | 1 | 105 | 315 | 0.997 |
| EMA | No | 2x4 | 0.3 | 1 | 6 | 24 | 0.032 |
| EMA | No | 2x4 | 0.3 | 1 | 8 | 32 | 0.081 |
| EMA | No | 2x4 | 0.3 | 1 | 12 | 48 | 0.288 |
| EMA | No | 2x4 | 0.3 | 1 | 14 | 56 | 0.432 |
| EMA | No | 2x4 | 0.3 | 1 | 16 | 64 | 0.51 |
| EMA | No | 2x4 | 0.3 | 1 | 18 | 72 | 0.592 |
| EMA | No | 2x4 | 0.3 | 1 | 20 | 80 | 0.638 |
| EMA | No | 2x4 | 0.3 | 1 | 22 | 88 | 0.7 |
| EMA | No | 2x4 | 0.3 | 1 | 30 | 120 | 0.833 |
| EMA | No | 2x4 | 0.3 | 1 | 36 | 144 | 0.909 |
| EMA | No | 2x4 | 0.3 | 1 | 40 | 160 | 0.947 |
| EMA | No | 2x4 | 0.3 | 1 | 44 | 176 | 0.963 |
| EMA | No | 2x4 | 0.3 | 1 | 50 | 200 | 0.98 |
| EMA | No | 2x4 | 0.3 | 1 | 54 | 216 | 0.986 |
| EMA | No | 2x4 | 0.3 | 1 | 60 | 240 | 0.991 |
| EMA | No | 2x4 | 0.3 | 1 | 66 | 264 | 0.998 |
| EMA | No | 2x4 | 0.3 | 1 | 72 | 288 | 0.997 |
| EMA | No | 2x4 | 0.3 | 1 | 80 | 320 | 0.999 |
| EMA | No | 2x4 | 0.4 | 1 | 6 | 24 | 0.002 |
| EMA | No | 2x4 | 0.4 | 1 | 12 | 48 | 0.152 |
| EMA | No | 2x4 | 0.4 | 1 | 18 | 72 | 0.435 |
| EMA | No | 2x4 | 0.4 | 1 | 20 | 80 | 0.5 |
| EMA | No | 2x4 | 0.4 | 1 | 24 | 96 | 0.635 |
| EMA | No | 2x4 | 0.4 | 1 | 28 | 112 | 0.706 |
| EMA | No | 2x4 | 0.4 | 1 | 32 | 128 | 0.781 |
| EMA | No | 2x4 | 0.4 | 1 | 34 | 136 | 0.837 |
| EMA | No | 2x4 | 0.4 | 1 | 38 | 152 | 0.863 |
| EMA | No | 2x4 | 0.4 | 1 | 42 | 168 | 0.89 |
| EMA | No | 2x4 | 0.4 | 1 | 48 | 192 | 0.959 |
| EMA | No | 2x4 | 0.4 | 1 | 54 | 216 | 0.968 |
| EMA | No | 2x4 | 0.4 | 1 | 60 | 240 | 0.979 |

| | | | | | | | |
|---|---|---|---|---|---|---|---|
| EMA | No | 2x4 | 0.4 | 1 | 66 | 264 | 0.987 |
| EMA | No | 2x4 | 0.4 | 1 | 72 | 288 | 0.996 |
| EMA | No | 2x4 | 0.4 | 1 | 80 | 320 | 0.993 |
| FDA | Yes | 2x2 | 0.3 | 1 | 12 | 24 | 0.018 |
| FDA | Yes | 2x2 | 0.3 | 1 | 16 | 32 | 0.044 |
| FDA | Yes | 2x2 | 0.3 | 1 | 24 | 48 | 0.228 |
| FDA | Yes | 2x2 | 0.3 | 1 | 28 | 56 | 0.338 |
| FDA | Yes | 2x2 | 0.3 | 1 | 32 | 64 | 0.445 |
| FDA | Yes | 2x2 | 0.3 | 1 | 36 | 72 | 0.53 |
| FDA | Yes | 2x2 | 0.3 | 1 | 40 | 80 | 0.61 |
| FDA | Yes | 2x2 | 0.3 | 1 | 44 | 88 | 0.662 |
| FDA | Yes | 2x2 | 0.3 | 1 | 60 | 120 | 0.834 |
| FDA | Yes | 2x2 | 0.3 | 1 | 72 | 144 | 0.921 |
| FDA | Yes | 2x2 | 0.3 | 1 | 80 | 160 | 0.948 |
| FDA | Yes | 2x2 | 0.3 | 1 | 88 | 176 | 0.978 |
| FDA | Yes | 2x2 | 0.3 | 1 | 100 | 200 | 0.987 |
| FDA | Yes | 2x2 | 0.3 | 1 | 108 | 216 | 0.992 |
| FDA | Yes | 2x2 | 0.3 | 1 | 120 | 240 | 0.995 |
| FDA | Yes | 2x2 | 0.3 | 1 | 132 | 264 | 0.999 |
| FDA | Yes | 2x2 | 0.3 | 1 | 144 | 288 | 0.999 |
| FDA | Yes | 2x2 | 0.3 | 1 | 160 | 320 | 0.999 |
| FDA | Yes | 2x2 | 0.4 | 1 | 12 | 24 | 0.007 |
| FDA | Yes | 2x2 | 0.4 | 1 | 24 | 48 | 0.07 |
| FDA | Yes | 2x2 | 0.4 | 1 | 36 | 72 | 0.29 |
| FDA | Yes | 2x2 | 0.4 | 1 | 40 | 80 | 0.385 |
| FDA | Yes | 2x2 | 0.4 | 1 | 48 | 96 | 0.521 |
| FDA | Yes | 2x2 | 0.4 | 1 | 56 | 112 | 0.65 |
| FDA | Yes | 2x2 | 0.4 | 1 | 64 | 128 | 0.737 |
| FDA | Yes | 2x2 | 0.4 | 1 | 68 | 136 | 0.787 |
| FDA | Yes | 2x2 | 0.4 | 1 | 76 | 152 | 0.809 |
| FDA | Yes | 2x2 | 0.4 | 1 | 84 | 168 | 0.866 |
| FDA | Yes | 2x2 | 0.4 | 1 | 96 | 192 | 0.924 |
| FDA | Yes | 2x2 | 0.4 | 1 | 108 | 216 | 0.944 |
| FDA | Yes | 2x2 | 0.4 | 1 | 120 | 240 | 0.971 |
| FDA | Yes | 2x2 | 0.4 | 1 | 132 | 264 | 0.978 |
| FDA | Yes | 2x2 | 0.4 | 1 | 144 | 288 | 0.985 |
| FDA | Yes | 2x2 | 0.4 | 1 | 160 | 320 | 0.994 |
| FDA | Yes | 3x3 | 0.3 | 1 | 9 | 27 | 0.07 |
| FDA | Yes | 3x3 | 0.3 | 1 | 12 | 36 | 0.145 |
| FDA | Yes | 3x3 | 0.3 | 1 | 15 | 45 | 0.276 |
| FDA | Yes | 3x3 | 0.3 | 1 | 18 | 54 | 0.389 |

| | | | | | | | |
|---|---|---|---|---|---|---|---|
| FDA | Yes | 3x3 | 0.3 | 1 | 21 | 63 | 0.494 |
| FDA | Yes | 3x3 | 0.3 | 1 | 24 | 72 | 0.579 |
| FDA | Yes | 3x3 | 0.3 | 1 | 27 | 81 | 0.677 |
| FDA | Yes | 3x3 | 0.3 | 1 | 30 | 90 | 0.749 |
| FDA | Yes | 3x3 | 0.3 | 1 | 39 | 117 | 0.832 |
| FDA | Yes | 3x3 | 0.3 | 1 | 48 | 144 | 0.937 |
| FDA | Yes | 3x3 | 0.3 | 1 | 54 | 162 | 0.943 |
| FDA | Yes | 3x3 | 0.3 | 1 | 60 | 180 | 0.977 |
| FDA | Yes | 3x3 | 0.3 | 1 | 66 | 198 | 0.985 |
| FDA | Yes | 3x3 | 0.3 | 1 | 75 | 225 | 0.992 |
| FDA | Yes | 3x3 | 0.3 | 1 | 81 | 243 | 0.995 |
| FDA | Yes | 3x3 | 0.3 | 1 | 90 | 270 | 0.995 |
| FDA | Yes | 3x3 | 0.3 | 1 | 96 | 288 | 0.999 |
| FDA | Yes | 3x3 | 0.3 | 1 | 105 | 315 | 0.998 |
| FDA | Yes | 3x3 | 0.4 | 1 | 9 | 27 | 0.094 |
| FDA | Yes | 3x3 | 0.4 | 1 | 15 | 45 | 0.265 |
| FDA | Yes | 3x3 | 0.4 | 1 | 24 | 72 | 0.605 |
| FDA | Yes | 3x3 | 0.4 | 1 | 27 | 81 | 0.708 |
| FDA | Yes | 3x3 | 0.4 | 1 | 33 | 99 | 0.816 |
| FDA | Yes | 3x3 | 0.4 | 1 | 39 | 117 | 0.902 |
| FDA | Yes | 3x3 | 0.4 | 1 | 42 | 126 | 0.92 |
| FDA | Yes | 3x3 | 0.4 | 1 | 45 | 135 | 0.949 |
| FDA | Yes | 3x3 | 0.4 | 1 | 51 | 153 | 0.951 |
| FDA | Yes | 3x3 | 0.4 | 1 | 54 | 162 | 0.97 |
| FDA | Yes | 3x3 | 0.4 | 1 | 63 | 189 | 0.996 |
| FDA | Yes | 3x3 | 0.4 | 1 | 72 | 216 | 0.996 |
| FDA | Yes | 3x3 | 0.4 | 1 | 81 | 243 | 0.999 |
| FDA | Yes | 3x3 | 0.4 | 1 | 90 | 270 | 1 |
| FDA | Yes | 3x3 | 0.4 | 1 | 96 | 288 | 1 |
| FDA | Yes | 3x3 | 0.4 | 1 | 105 | 315 | 1 |
| FDA | Yes | 2x4 | 0.3 | 1 | 6 | 24 | 0.041 |
| FDA | Yes | 2x4 | 0.3 | 1 | 8 | 32 | 0.058 |
| FDA | Yes | 2x4 | 0.3 | 1 | 12 | 48 | 0.15 |
| FDA | Yes | 2x4 | 0.3 | 1 | 14 | 56 | 0.234 |
| FDA | Yes | 2x4 | 0.3 | 1 | 16 | 64 | 0.308 |
| FDA | Yes | 2x4 | 0.3 | 1 | 18 | 72 | 0.399 |
| FDA | Yes | 2x4 | 0.3 | 1 | 20 | 80 | 0.471 |
| FDA | Yes | 2x4 | 0.3 | 1 | 22 | 88 | 0.557 |
| FDA | Yes | 2x4 | 0.3 | 1 | 30 | 120 | 0.764 |
| FDA | Yes | 2x4 | 0.3 | 1 | 36 | 144 | 0.855 |
| FDA | Yes | 2x4 | 0.3 | 1 | 40 | 160 | 0.89 |

| | | | | | | | |
|---|---|---|---|---|---|---|---|
| FDA | Yes | 2x4 | 0.3 | 1 | 44 | 176 | 0.93 |
| FDA | Yes | 2x4 | 0.3 | 1 | 50 | 200 | 0.949 |
| FDA | Yes | 2x4 | 0.3 | 1 | 54 | 216 | 0.965 |
| FDA | Yes | 2x4 | 0.3 | 1 | 60 | 240 | 0.982 |
| FDA | Yes | 2x4 | 0.3 | 1 | 66 | 264 | 0.992 |
| FDA | Yes | 2x4 | 0.3 | 1 | 72 | 288 | 0.993 |
| FDA | Yes | 2x4 | 0.3 | 1 | 80 | 320 | 0.996 |
| FDA | Yes | 2x4 | 0.4 | 1 | 6 | 24 | 0.058 |
| FDA | Yes | 2x4 | 0.4 | 1 | 12 | 48 | 0.265 |
| FDA | Yes | 2x4 | 0.4 | 1 | 18 | 72 | 0.557 |
| FDA | Yes | 2x4 | 0.4 | 1 | 20 | 80 | 0.638 |
| FDA | Yes | 2x4 | 0.4 | 1 | 24 | 96 | 0.75 |
| FDA | Yes | 2x4 | 0.4 | 1 | 28 | 112 | 0.827 |
| FDA | Yes | 2x4 | 0.4 | 1 | 32 | 128 | 0.871 |
| FDA | Yes | 2x4 | 0.4 | 1 | 34 | 136 | 0.909 |
| FDA | Yes | 2x4 | 0.4 | 1 | 38 | 152 | 0.944 |
| FDA | Yes | 2x4 | 0.4 | 1 | 42 | 168 | 0.966 |
| FDA | Yes | 2x4 | 0.4 | 1 | 48 | 192 | 0.986 |
| FDA | Yes | 2x4 | 0.4 | 1 | 54 | 216 | 0.989 |
| FDA | Yes | 2x4 | 0.4 | 1 | 60 | 240 | 0.998 |
| FDA | Yes | 2x4 | 0.4 | 1 | 66 | 264 | 1 |
| FDA | Yes | 2x4 | 0.4 | 1 | 72 | 288 | 1 |
| FDA | Yes | 2x4 | 0.4 | 1 | 80 | 320 | 1 |
| FDA | No | 2x2 | 0.3 | 1 | 12 | 24 | 0.028 |
| FDA | No | 2x2 | 0.3 | 1 | 16 | 32 | 0.067 |
| FDA | No | 2x2 | 0.3 | 1 | 24 | 48 | 0.259 |
| FDA | No | 2x2 | 0.3 | 1 | 28 | 56 | 0.383 |
| FDA | No | 2x2 | 0.3 | 1 | 32 | 64 | 0.498 |
| FDA | No | 2x2 | 0.3 | 1 | 36 | 72 | 0.58 |
| FDA | No | 2x2 | 0.3 | 1 | 40 | 80 | 0.641 |
| FDA | No | 2x2 | 0.3 | 1 | 44 | 88 | 0.693 |
| FDA | No | 2x2 | 0.3 | 1 | 60 | 120 | 0.876 |
| FDA | No | 2x2 | 0.3 | 1 | 72 | 144 | 0.935 |
| FDA | No | 2x2 | 0.3 | 1 | 80 | 160 | 0.955 |
| FDA | No | 2x2 | 0.3 | 1 | 88 | 176 | 0.982 |
| FDA | No | 2x2 | 0.3 | 1 | 100 | 200 | 0.986 |
| FDA | No | 2x2 | 0.3 | 1 | 108 | 216 | 0.994 |
| FDA | No | 2x2 | 0.3 | 1 | 120 | 240 | 0.997 |
| FDA | No | 2x2 | 0.3 | 1 | 132 | 264 | 0.999 |
| FDA | No | 2x2 | 0.3 | 1 | 144 | 288 | 0.999 |
| FDA | No | 2x2 | 0.3 | 1 | 160 | 320 | 1 |

| | | | | | | | |
|---|---|---|---|---|---|---|---|
| FDA | No | 2x2 | 0.4 | 1 | 12 | 24 | 0.01 |
| FDA | No | 2x2 | 0.4 | 1 | 24 | 48 | 0.087 |
| FDA | No | 2x2 | 0.4 | 1 | 36 | 72 | 0.317 |
| FDA | No | 2x2 | 0.4 | 1 | 40 | 80 | 0.396 |
| FDA | No | 2x2 | 0.4 | 1 | 48 | 96 | 0.558 |
| FDA | No | 2x2 | 0.4 | 1 | 56 | 112 | 0.666 |
| FDA | No | 2x2 | 0.4 | 1 | 64 | 128 | 0.751 |
| FDA | No | 2x2 | 0.4 | 1 | 68 | 136 | 0.804 |
| FDA | No | 2x2 | 0.4 | 1 | 76 | 152 | 0.828 |
| FDA | No | 2x2 | 0.4 | 1 | 84 | 168 | 0.893 |
| FDA | No | 2x2 | 0.4 | 1 | 96 | 192 | 0.938 |
| FDA | No | 2x2 | 0.4 | 1 | 108 | 216 | 0.95 |
| FDA | No | 2x2 | 0.4 | 1 | 120 | 240 | 0.975 |
| FDA | No | 2x2 | 0.4 | 1 | 132 | 264 | 0.984 |
| FDA | No | 2x2 | 0.4 | 1 | 144 | 288 | 0.987 |
| FDA | No | 2x2 | 0.4 | 1 | 160 | 320 | 0.995 |
| FDA | No | 3x3 | 0.3 | 1 | 9 | 27 | 0.082 |
| FDA | No | 3x3 | 0.3 | 1 | 12 | 36 | 0.16 |
| FDA | No | 3x3 | 0.3 | 1 | 15 | 45 | 0.284 |
| FDA | No | 3x3 | 0.3 | 1 | 18 | 54 | 0.395 |
| FDA | No | 3x3 | 0.3 | 1 | 21 | 63 | 0.5 |
| FDA | No | 3x3 | 0.3 | 1 | 24 | 72 | 0.563 |
| FDA | No | 3x3 | 0.3 | 1 | 27 | 81 | 0.681 |
| FDA | No | 3x3 | 0.3 | 1 | 30 | 90 | 0.745 |
| FDA | No | 3x3 | 0.3 | 1 | 39 | 117 | 0.845 |
| FDA | No | 3x3 | 0.3 | 1 | 48 | 144 | 0.938 |
| FDA | No | 3x3 | 0.3 | 1 | 54 | 162 | 0.948 |
| FDA | No | 3x3 | 0.3 | 1 | 60 | 180 | 0.969 |
| FDA | No | 3x3 | 0.3 | 1 | 66 | 198 | 0.981 |
| FDA | No | 3x3 | 0.3 | 1 | 75 | 225 | 0.995 |
| FDA | No | 3x3 | 0.3 | 1 | 81 | 243 | 0.995 |
| FDA | No | 3x3 | 0.3 | 1 | 90 | 270 | 0.996 |
| FDA | No | 3x3 | 0.3 | 1 | 96 | 288 | 0.998 |
| FDA | No | 3x3 | 0.3 | 1 | 105 | 315 | 0.996 |
| FDA | No | 3x3 | 0.4 | 1 | 9 | 27 | 0.096 |
| FDA | No | 3x3 | 0.4 | 1 | 15 | 45 | 0.278 |
| FDA | No | 3x3 | 0.4 | 1 | 24 | 72 | 0.598 |
| FDA | No | 3x3 | 0.4 | 1 | 27 | 81 | 0.72 |
| FDA | No | 3x3 | 0.4 | 1 | 33 | 99 | 0.808 |
| FDA | No | 3x3 | 0.4 | 1 | 39 | 117 | 0.904 |
| FDA | No | 3x3 | 0.4 | 1 | 42 | 126 | 0.925 |

| | | | | | | | |
|---|---|---|---|---|---|---|---|
| FDA | No | 3x3 | 0.4 | 1 | 45 | 135 | 0.946 |
| FDA | No | 3x3 | 0.4 | 1 | 51 | 153 | 0.952 |
| FDA | No | 3x3 | 0.4 | 1 | 54 | 162 | 0.976 |
| FDA | No | 3x3 | 0.4 | 1 | 63 | 189 | 0.992 |
| FDA | No | 3x3 | 0.4 | 1 | 72 | 216 | 0.997 |
| FDA | No | 3x3 | 0.4 | 1 | 81 | 243 | 0.998 |
| FDA | No | 3x3 | 0.4 | 1 | 90 | 270 | 1 |
| FDA | No | 3x3 | 0.4 | 1 | 96 | 288 | 0.999 |
| FDA | No | 3x3 | 0.4 | 1 | 105 | 315 | 0.999 |
| FDA | No | 2x4 | 0.3 | 1 | 6 | 24 | 0.035 |
| FDA | No | 2x4 | 0.3 | 1 | 8 | 32 | 0.057 |
| FDA | No | 2x4 | 0.3 | 1 | 12 | 48 | 0.163 |
| FDA | No | 2x4 | 0.3 | 1 | 14 | 56 | 0.231 |
| FDA | No | 2x4 | 0.3 | 1 | 16 | 64 | 0.316 |
| FDA | No | 2x4 | 0.3 | 1 | 18 | 72 | 0.397 |
| FDA | No | 2x4 | 0.3 | 1 | 20 | 80 | 0.458 |
| FDA | No | 2x4 | 0.3 | 1 | 22 | 88 | 0.546 |
| FDA | No | 2x4 | 0.3 | 1 | 30 | 120 | 0.745 |
| FDA | No | 2x4 | 0.3 | 1 | 36 | 144 | 0.847 |
| FDA | No | 2x4 | 0.3 | 1 | 40 | 160 | 0.887 |
| FDA | No | 2x4 | 0.3 | 1 | 44 | 176 | 0.924 |
| FDA | No | 2x4 | 0.3 | 1 | 50 | 200 | 0.949 |
| FDA | No | 2x4 | 0.3 | 1 | 54 | 216 | 0.962 |
| FDA | No | 2x4 | 0.3 | 1 | 60 | 240 | 0.979 |
| FDA | No | 2x4 | 0.3 | 1 | 66 | 264 | 0.991 |
| FDA | No | 2x4 | 0.3 | 1 | 72 | 288 | 0.993 |
| FDA | No | 2x4 | 0.3 | 1 | 80 | 320 | 0.997 |
| FDA | No | 2x4 | 0.4 | 1 | 6 | 24 | 0.047 |
| FDA | No | 2x4 | 0.4 | 1 | 12 | 48 | 0.266 |
| FDA | No | 2x4 | 0.4 | 1 | 18 | 72 | 0.558 |
| FDA | No | 2x4 | 0.4 | 1 | 20 | 80 | 0.627 |
| FDA | No | 2x4 | 0.4 | 1 | 24 | 96 | 0.74 |
| FDA | No | 2x4 | 0.4 | 1 | 28 | 112 | 0.795 |
| FDA | No | 2x4 | 0.4 | 1 | 32 | 128 | 0.86 |
| FDA | No | 2x4 | 0.4 | 1 | 34 | 136 | 0.91 |
| FDA | No | 2x4 | 0.4 | 1 | 38 | 152 | 0.938 |
| FDA | No | 2x4 | 0.4 | 1 | 42 | 168 | 0.96 |
| FDA | No | 2x4 | 0.4 | 1 | 48 | 192 | 0.989 |
| FDA | No | 2x4 | 0.4 | 1 | 54 | 216 | 0.989 |
| FDA | No | 2x4 | 0.4 | 1 | 60 | 240 | 0.997 |
| FDA | No | 2x4 | 0.4 | 1 | 66 | 264 | 0.999 |

| | | | | | | | |
|---|---|---|---|---|---|---|---|
| FDA | No | 2x4 | 0.4 | 1 | 72 | 288 | 1 |
| FDA | No | 2x4 | 0.4 | 1 | 80 | 320 | 1 |
